# Comparative proteomic analyses of Asian cotton ovules with attached fibers in the early stages of fiber elongation process

**DOI:** 10.1186/s12953-016-0101-1

**Published:** 2016-09-08

**Authors:** Bing Zhang, Shao-Jun Du, Jue Hu, Di Miao, Jin-Yuan Liu

**Affiliations:** Laboratory of Plant Molecular Biology, Center for Plant Biology, School of Life Sciences, Tsinghua University, Beijing, 100084 People’s Republic of China

**Keywords:** Asian cotton, Ovule, Fiber elongation, Differentially displayed protein spots, Polyploidization

## Abstract

**Background:**

Plenty of proteomic studies were performed to characterize the allotetraploid upland cotton fiber elongation process, whereas little is known about the elongating diploid cotton fiber proteome.

**Methods:**

In this study, we used a two-dimensional electrophoresis-based comparative proteomic approach to profile dynamic proteomes of diploid Asian cotton ovules with attached fibers in the early stages of fiber elongation process. One-way ANOVA and Student-Newman-Keuls test were used to find the differentially displayed protein (DDP) spots.

**Results:**

A total of 55 protein spots were found having different abundance ranging from 1 to 9 days post-anthesis (DPA) in a two-day interval. These 55 DDP spots were all successfully identified using high-resolution mass spectrometric analyses. Gene ontology analyses revealed that proteoforms involved in energy/carbohydrate metabolism, redox homeostasis, and protein metabolism are the most abundant. In addition, orthologues of the 13 DDP spots were also found in differential proteome of allotetraploid elongating cotton fibers, suggesting their possible essential roles in fiber elongation process.

**Conclusions:**

Our results not only revealed the dynamic proteome change of diploid Asian cotton fiber and ovule during early stages of fiber elongation process but also provided valuable resource for future studies on the molecular mechanism how the polyploidization improves the trait of fiber length.

**Electronic supplementary material:**

The online version of this article (doi:10.1186/s12953-016-0101-1) contains supplementary material, which is available to authorized users.

## Background

As a major source of natural fiber in the world, cotton has been widely used for clothing, papermaking, and other purposes for thousands of years [1]. Apart from its economic value, cotton is also known as an excellent model system for studying cell differentiation, cell elongation and cellulose biosynthesis [2]. All cotton plants belong to the genus *Gossypium* in the family Malvaceae. Among the around 50 known *Gossypium* species, only four cultivated species, *G. arboreum*, *G. herbaceum*, *G. hirsutum*, and *G. barbadense*, can produce spinnable fibers. The first two cultivated species are diploids (AA) and the last two are allotetraploids (AADD) originating by a polyploidization event involving *G. herbaceum* and a diploid hairiness species resembling *G. raimondii* (DD) [3]. Interestingly, the fiber length of the two allotetraploids species are longer than the two diploids species. Considering that the progenitor DD genome species are fiber deficient, the long-fiber phenotype of allotetraploid cotton species must be formed in the polyploidization process through some unknown complex mechanism [4–6]. Elucidation of the molecular mechanism of fiber elongation in the diploid cotton and further comparison of the differences of fiber elongation between the diploid and allotetraploid cotton could help us understand how the polyploidization improves the traits of fiber length [7].

With the availability of high-quality EST sequences in public databases, high-throughput proteomic analyses of cotton fiber elongation process were successfully performed in advance of the cotton genome sequencing project. For example, a proteomic study of the *ligon* lintless mutant and wild-type upland cotton fibers identified 81 differentially displayed protein (DDP) spots at 14 days post-anthesis (DPA), suggesting that proteins involved in protein folding and stabilization are important for fiber elongation [8]. Another study that compared the proteomic profiles of wild-type and fuzzless-lintless mutant upland cotton (*G. hirsutum* cultivar Xu142) fibers at 10 DPA identified 104 DDP spots, providing evidence that pectin synthesis is imperative for fiber elongation [9]. In another 2-DE-based comparative proteomic analysis, a total of 235 protein spots were found having different abundance during the entire elongation process at five distinct time points: 5, 10, 15, 20 and 25 DPA of upland cotton (*G. hirsutum* cultivar CRI 35) [10]. Further Kyoto Encyclopedia of Genes and Genomes (KEGG) Orthology-based Annotation System (KOBAS) analyses based on the identified 235 DDPs indicated that glycolysis is the most significantly regulated biochemical pathway during the fiber elongation process [11]. With the availability of cotton genome sequences, post-genomic proteomics studies further shed many new insights into cotton fiber initiation and elongation processes [12–16]. Hu et al. successfully compared the total proteome of two allotetraploid cotton species (*G. hirsutum* and *G. barbadense*) and their diploid parents at 10 and 20 DPA, providing the first evidence that the two allopolyploid species have achieved superficially similar modern fiber phenotypes through different evolutionary routes [17]. However, the mechanisms of which proteins are essential for fiber elongation and how polyploidization increases the fiber length of the allotetraploid upland cotton are poorly understood. Furthermore, because most of these proteomic studies focused on the midst and late stage of fiber elongation process, the early events occur in the fiber elongation process are still poorly characterized at the proteome level.

In the present study, we reported the first comparative proteomic analyses of fiber and ovule proteome of diploid Asian cotton (*G. arboreum* cultivar DPL971) in the early stages of elongation process (1-9 DPA) and the identification of 55 DDPs of developing diploid cotton ovules with attached fibers. Through comparing the dynamic proteome of Asian cotton ovules and attached fibers with differential proteome of upland cotton fibers, we clarified 13 possible essential proteins required for fiber elongation and four important proteins whose increased abundance are correlated with improved fiber length.

## Results and discussions

### Dynamic proteomes of diploid Asian cotton ovules and fibers in the early stages of fiber elongation process

Fiber length measurement indicated that the diploid Asian cotton (*G. arboreum* cultivar DPL971) shows a gradual increase of fiber length during the early stages of fiber elongation process as the allotetraploid upland cotton (*G. hirsutum* cultivar CRI35) does (Additional file 1: Figure S1). The fastest fiber elongation rate was observed at 5 DPA in both two cultivars. Since then, the elongation rate gradually slowed down. Interestingly, the fiber length of cultivar DPL971 is always shorter than that of cultivar CRI35 (Additional file 1: Figure S1), supporting the notion that polyploidization improves the fiber length trait.

**Fig. 1:**
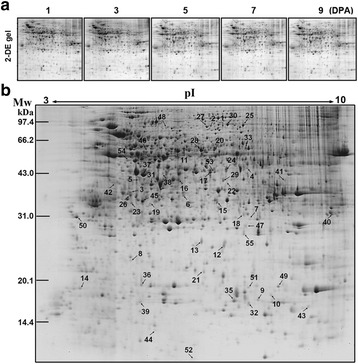
2-DE map of the 55 identified differentially displayed protein spots. **a** Representative 2-DE gel of total proteins extracted from diploid Asian cotton ovules with attached fibers at five time points. **b** 2-DE map of total proteins extracted from 5 DPA diploid Asian cotton ovules with attached fibers (IPG strip pH 3–10 NL, 12.5 % SDS-PAGE, blue silver staining). 55 differentially displayed protein (DDP) spots are all indicated on the 2-DE map

To characterize the dynamic proteome change of cotton ovules and fibers in the early stages of fiber elongation process, total protein extracted from ovules with attached fibers of Asian cotton cultivar DPL971 at five stages (1, 3, 5, 7 and 9 DPA) were separated by 2-DE (Fig. 1a). Approximately 1800 stained protein spots could be reproducibly detected on each 2-D gel, and most of these protein spots showed no significant variance between any two stages. Only 55 protein spots were statistically analyzed being dynamic with their abundance has a greater than two-fold variation (*p*-value < 0.05) during the fiber elongation process (protein abundance at 3-9 DPA in comparison with that at 1 DPA) using one-way ANOVA and Student-Newman-Keuls tests (Fig. 1b and Additional file 2: Table S1). The 55 distinct spots were manually excised from the 2-DE gels and digested with trypsin. Finally, 55 spots selected for mass spectrometry analysis were all successfully identified and represented 55 distinct proteoforms [18] and 52 unique proteins (Additional file 3: Table S2 and Additional file 4).

**Fig. 2:**
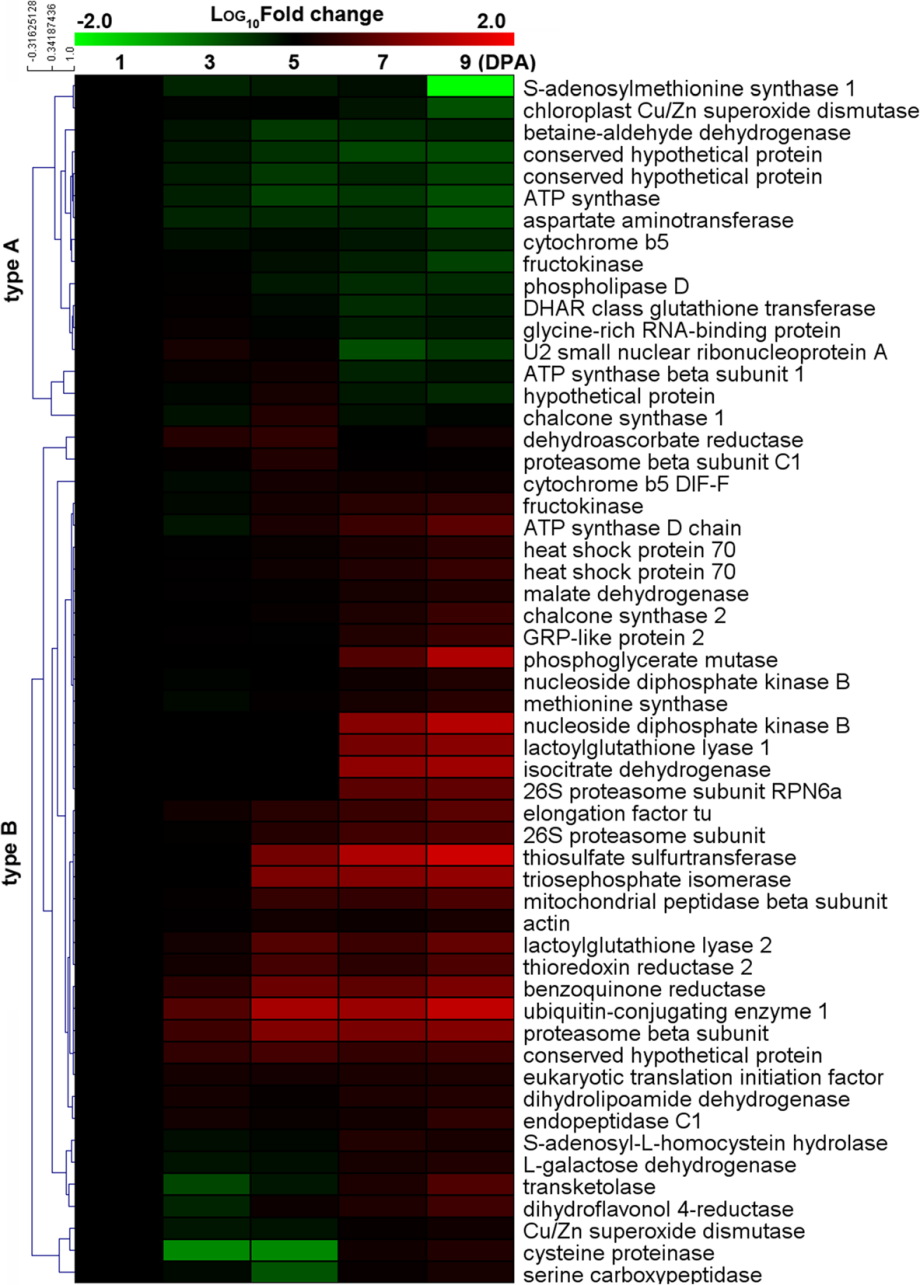
Protein abundance variance analysis of the 55 differentially displayed protein spots. Complete linkage hierarchical cluster analysis of the 55 DDPs at five time points was performed by comparing the average fold change of the DDPs. Protein names are indicated at the right. Type A and B stands for the proteins preferentially expressed in initiation and elongation process, respectively

**Fig. 3:**
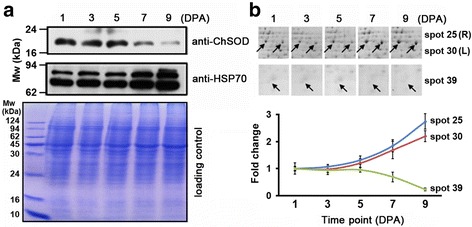
Comparison of expression profiles of two selected proteins. **a** Western blot assay was performed using peptide-specific antibodies (detailed in Materials and Methods) against chloroplast Cu/Zn superoxide dismutase (anti-ChSOD) and heat shock protein 70 (anti-HSP70). The experiments were repeated for three times using different ovule attached with fiber samples with similar results. The 2-DE image and fold change of chloroplast Cu/Zn superoxide dismutase (spot No.39) and heat shock protein 70 (spot No.25 and 30) were shown in (**b**) based on their Vol% values

The 55 DDP spots were clustered to two types according to their expression patterns (Fig. 2). The type A class contains 16 DDP spots, all of which have a higher protein abundance at 1 and 3 DPA, whereas the remaining 39 DDP spots were all grouped in the type B class having a higher protein abundance between 5 and 9 DPA. Two proteins, chloroplast Cu/Zn superoxide dismutase and heat shock protein 70, belongs to type A and type B class, respectively. Western blot analysis indicated that expression of chloroplast Cu/Zn superoxide dismutase is gradually decreasing during 1–9 DPA, whereas protein abundance of the two isoforms of heat shock protein 70 are both gradually increasing during the same period (Fig. 3a). These results are in full agreement with the protein abundance variance revealed by 2-DE and mass spectrometry identification (Fig. 3b), confirming the correctness of our analyses. Interesting, initiation of cotton fiber from the ovule epidermis occurs from -3 DPA to 3 DPA, whereas the fast fiber elongation process generally starts at 5 DPA and ends at 25 DPA [2–4]. Thus, the two types of DDP spots correspond exactly to proteins preferentially expressed in the fiber initiation and elongation process, respectively.

### Functional analyses of the differentially displayed proteins of diploid Asian cotton ovules and fibers in the early stages of fiber elongation process

**Fig. 4:**
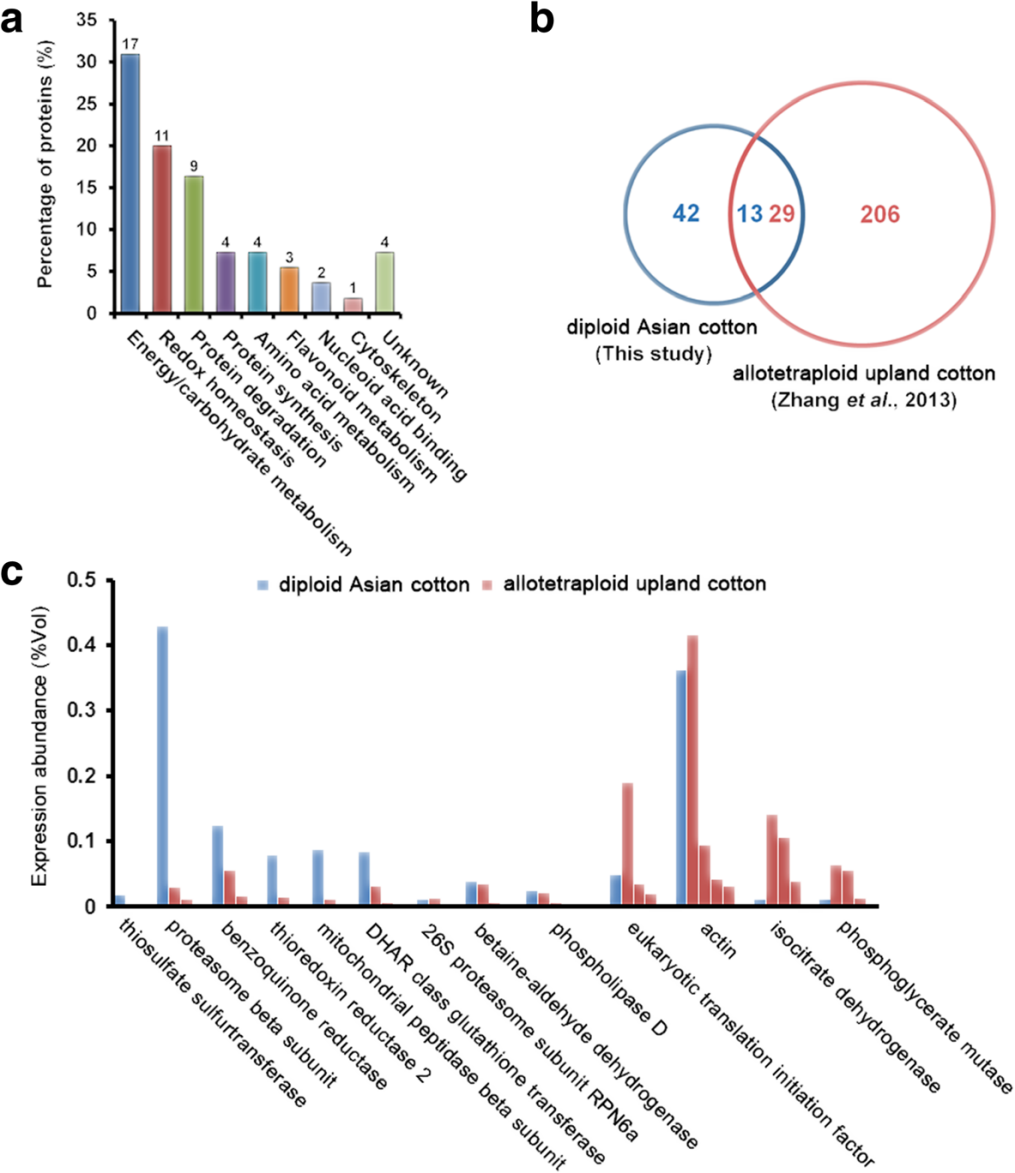
Functional analysis of the 55 differentially displayed protein spots. **a** Functional classification of the 55 DDP spots. The number of identities in each group are shown on the top. **b** Venn diagram analysis of the 55 DDP spots in this study and 235 DDP spots of elongating allotetraploid cotton fibers indicated that 13 DDPs in diploid Asian cotton have different number of orthologues (total number: 29) in allotetraploid upland cotton. **c** Comparison of the protein abundance at 5 DPA between the 13 DDPs in diploid Asian cotton and 29 homologous DDPs in allotetraploid upland cotton. The nine DDPs having more than one homologous DDPs were compared with all the DDPs of allotetraploid upland cotton in the same group

AgriGO analysis indicated that the 55 DDPs could be divided into nine functional groups (Fig. 4a). Of these groups, energy/carbohydrate metabolism, including both glycolysis and the TCA cycle (17 DDPs), redox homeostasis (11 DDPs), and protein degradation (9 DDPs) contains the largest number of DDPs. This result is understandable because energy supplement is prerequisite for rapid fiber cell elongation [2, 4], whereas fast cell growth, such as the cotton fiber elongation process, needs an intracellular oxidation-reduction equilibrium [19] and a fast protein turnover rate [2, 8]. In addition, protein synthesis, amino acid and flavonoid metabolism also contained more than three DDPs, suggesting their possible important roles in cotton fiber elongation process [12, 14]. Moreover, different DDPs of each functional groups have unique protein abundance variance (Fig. 2), suggesting their expression were specifically regulated in developing Asian cotton ovules and fibers.

Many of the 55 DDPs were known important proteins required for proper fiber initiation and elongation. For example, DDP No. 31 and 39 were identified as two different Cu/Zn superoxide dismutase, which was previously characterized to be important for cotton fiber development [20]. Similarly, DDP No. 14, 40 and 54 were all identified as ATP synthase subunits, which was previously proved playing vital roles in cotton fiber elongation process [21]. The activity of malate dehydrogenase (DDP No.34) is variant among cotton cultivars with differing fiber traits [22], whereas chalcone synthase (DDP No.24 and 53) and dihydroflavonol 4-reductase (DDP No.16), two important enzymes involved in flavonoid metabolism, were reported being related to cotton fiber quality [23, 24].

Furthermore, although the function of some DDPs are unclear in cotton, investigation of the gene homologs in model plant Arabidopsis also implied their important functions in ovule and fiber development. For example, DDP No. 06 was identified as a thiosulfate sulfurtransferase, which plays important roles in embryo and seed development in *Arabidopsis thaliana* [25]. DDP No. 33 was identified as a dihydrolipoamide dehydrogenase, the E3 subunit of pyruvate dehydrogenase complex. In *Arabidopsis thaliana*, mutations of plastid pyruvate dehydrogenase complex will lead to an early embryo lethal phenotype, suggesting the important function of pyruvate dehydrogenase complex in embryo development [26]. During fiber development process, the fiber cells are always attached with the ovules which provide the fiber cells essential water, carbon source and mineral nutrients [27]. Identification of the important proteins required for embryo/ovule development in this study could give us the clue how the cotton plants delicately regulate the complex sink and source relationship of ovule and fiber to promote the fast elongation of fiber cells. This important information couldn’t be obtained through studies only focusing on fibers.

### Comparative analyses of the differentially displayed proteins of diploid Asian cotton ovules and fibers with allotetraploid upland cotton fibers

Blast search indicated that 13 of the 55 DDPs of diploid Asian cotton ovules with attached fibers in early stages of fiber elongation process were also identified in the comparative proteomic analyses of the elongating allotetraploid upland cotton fiber cells [10, 11], suggesting these 13 DDPs are required for fiber elongation (Fig. 4b). Moreover, nine of the 13 DDPs were found having more copies in allotetraploid upland cotton fiber cells than in diploid Asian cotton fiber/ovule cells (Fig. 4c). Notably, the different copies of the selected DDPs in allotetraploid upland cotton all have the same protein sequence, suggesting these different proteoforms are differentially post-translationally modified. For example, four DDPs of phospholipase D alpha protein were identified in allotetraploid fiber with the same NCBI accession number whereas only one DDP was identified in diploid fiber/ovule.

Comparison of the protein abundance between the 13 DDPs in diploid Asian cotton and the homologous 29 DDPs in allotetraploid upland cotton at 5 DPA, the same time point of fiber elongation process, further revealed that protein abundance of four proteins, including translation initiation factor, actin, isocitrate dehydrogenase and phosphoglycerate mutase, were all increased in allotetraploid upland cotton (Fig. 4c). Moreover, DDP number of the four proteins were also increased in allotetraploid upland cotton. These results strongly suggested that activity of the four proteins might be selectively up-regulated in the polyploidization process, implying the four proteins are important for improving the fiber length trait. In agreement with this suspicion, fiber length of the transgenic cottons expressing the actin gene is significantly longer than that of wild-type cotton plants [28].

## Conclusions

In summary, we reported the first comparative proteomics study of diploid Asian cotton ovules with attached fibers in the early stages of fiber elongation process. Combined with the proteome dataset of elongating allotetraploid upland cotton fibers, our study provides a reference list of essential proteins supporting the fast cotton fiber elongation process. Information of these essential proteins including protein expression level and MS identification data provide a valuable resource for future functional studies.

## Methods

### Plant materials

Asian cotton (*G. arboreum* cultivar DPL971) and upland cotton (*G. hirsutum* cultivar CRI35) was grown in a standard agronomic field during the period from April to September in Beijing. The ages of the ovules with attached fibers selected for total protein extraction and fiber length measurement were 1, 3, 5, 7 and 9 DPA. All of the collected samples were frozen in liquid nitrogen and then stored at −80 °C for protein extraction.

### Measurements of fiber length

Ovules were detached from the fresh cotton bolls and boiled at 95 °C water bath for 10 min. Aggregated fibers were combed in water, observed and photographed under a SZX12 anatomy microscope (Olympus, Japan) equipped with a DP70 digital camera system. Five ovules were used to represent each developmental stage whereas length of 10 fibers were recorded for each ovule.

### Protein extraction

Total protein was extracted using modified Tris-phenol method as described [29]. About 1 g of frozen cotton ovules with attached fibers was ground with 10 % PVPP (w/w) and 10 % quartz sand (w/w) in liquid nitrogen using a mortar and pestle. The powder was suspended completely in twenty milliliters of ice-cold acetone (adding 2 % β-mercaptoethanol) and centrifuged at 12,000 g for 15 min at 4 °C to wash away impurities, and this step was repeated twice. The freeze-dried powder was homogenized in 5 mL of extraction buffer containing 50 mM Tris–HCl, pH 8.6, 2 % SDS, 2 % (w/w) β-mercaptoethanol, 1 mM PMSF, and then an equal volume of Tris saturated phenol (pH 8.0) was added. The mixture was vortexed thoroughly for 5 min, and the phenol phase was collected and precipitated with 5 volume of 0.1 M ammonium acetate in methanol at −20 °C for 30 min. After centrifuging at 12, 000 g for 15 min, the collected protein pellets were washed three times with cold 0.1 M ammonium acetate in methanol, and then washed three times with cold 80 % acetone in water. The lyophilized pellets were dissolved in rehydration buffer (7 M urea, 2 M thiourea, 4 % CHAPS, 1 % IPG buffer, 20 mM DTT) and centrifuged at 12 000 g for 15 min to remove insoluble materials. The concentration of extracted proteins was quantified using Bradford method with biotechnology grade BSA protein as a quantification standard [30]. The proteins underwent 2-DE immediately or were stored at −80 °C.

### 2-DE and image analyses

2-DE was performed according to the manufacturer’s instruction (2-DE Manual, GE Healthcare). 1 mg protein mixed with rehydration buffer (7 M urea, 2 M thiourea, 4 % CHAPS, 1 % IPG buffer, 20 mM DTT) in a total volume of 1 mL, was loaded onto a nonlinear IPG Drystrip (pH 3-10, 24 cm, GE Healthcare, Piscataway, USA). The strips were hydrated in the rehydration buffer for 18 h at room temperature. Then isoelectric focusing was performed on an Ettan IPGphor isoelectric focusing system (GE Healthcare, Uppsala, Sweden) under the following conditions: 100 V for 40 min, 500 V for 40 min, 1000 V for 1 h, 4000 V for 2 h, and 8000 V for 8 h until total voltage hours of 75,000 was achieved. Before SDS-PAGE analysis, strips were incubated for 2 × 15 min in equilibration buffer (6 M urea, 50 mM pH 8.8 Tris–HCl, 30 % (v/v) glycerol, 2 % (w/v) SDS, a trace of bromophenol blue). One percent DTT (w/v) was added to the above for the first 15 min and 2.5 % iodoacetamide (w/v) instead for the second 15 min. After equilibration, strips were placed on top of a vertical 12.5 % SDS-polyacrylamide self-cast gel and electrophoresis was performed at 4 °C and 5 W/gel for 45 min, and then 17 W/gel for 5 h until the dye front reached the bottom of gels. For calculation of molecular weight (MW) of the 2-DE protein spots, a filter paper piece pre-loaded with a protein marker (14.4–97.4 kDa) were placed along with the equilibrated strip on top of the gels. After electrophoresis, 2-D gels were stained by Colloidal Coomassie Blue G-250 [31]. The 2-D gels were scanned at 600 dpi resolution using a UMAX PowerLook 2100XL scanner (Willich, Germany) with following parameters: scan mode, transparent; color, grey; calibration, auto calibration. Image analysis was performed with ImageMaster Platinum software (version 6.0) (GE Healthcare). Proteins extracted from three different samples of each time point were analyzed by 2-DE and triplicates were applied to each protein sample, thus a total of 45 CBB-stained 2-D gel images were obtained. The spots were quantified using the % volume criterion. The match analysis was done in automatic mode using the following detection parameters: Smooth 4, Saliency 300, and Min Area 29, and further manual editing was performed to correct the mismatched and unmatched spots. The relative volume of each spot was assumed to represent its expression abundance. A significant difference was defined by the criterion *p*-value < 0.05 when analyzing parallel spots between groups with one-way ANOVA and Student-Newman-Keuls test using the SPSS 16.0 statistical software (IBM).

### Protein identification by MALDI-TOF/TOF

Protein spots were manually excised from the 2-D gels and digested with trypsin. Briefly, the excised gel pieces were destained with 100 mL of 25 mM NH_4_HCO_3_ in 50 % ACN until the stain faded sufficiently. After that, the gel pieces were washed twice in 100 % ACN for 10 min and then dried under vacuum for 15 min. Proteins in the gel pieces were digested in 25 mM NH_4_HCO_3_, 10 ng/mL trypsin overnight at 37C. The digestion solutions were used in the subsequent MS analysis. The 4800 MALDI TOF/TOF™ Analyzer (Applied Biosystems, Framingham, USA) was used for protein identification. The MS spectra were acquired in the positive ion reflector mode, with a mass range from 800 to 4000 Da. The peaks with S/N > 20 were selected for PMF analysis. The MS/MS analysis was performed with the 10 strongest peaks of the MS spectra, and the precursor ions were accelerated at a voltage of 8 kV. The MS/MS spectra were accumulated for at least 2000 laser shots. All of the MS and MS/MS data were analyzed in the combined mode using Global Proteome Server Explorer (software version 3.5, Applied Biosystems) to interface with the Mascot 2.2 search engine (Matrix Science) against a *G. arboreum* peptide sequence database downloaded from Cotton Genome Project website (http://cgp.genomics.org.cn, including 41,331 protein sequences, 14,876,209 residues) [32]. Searching parameters were set as follows: S/N ≥ 3.0; fixed modification, carbamidomethyl (Cys); variable modification, oxidation (Met); maximum number of missing cleavages, 1; MS tolerance, ± 0.1 Da; MS/MS tolerance, ± 0.5 Da. The identities with the highest score were subsequently analyzed using blast tools against the Uniprot database (http://www.uniprot.org/) to obtain the gene ontology (GO) annotation.

### Antibody preparation and western blot

The specific antibodies against selected proteins, chloroplast Cu/Zn superoxide dismutase (ChSOD) and heat shock protein 70 (HSP70), were prepared by the immunisation of rabbits with synthesised protein-specific peptides (MAAHIFTTTPSHL for ChSOD and RSFRRRDIAASPL for HSP70) mixed with Freund adjuvant by Beijing Protein Innovation (Beijing, China). Affinity purifications were further performed to enrich antibodies to ensure the titers against synthesised protein-specific peptides larger than 1: 6400 by ELISA. Goat anti-rabbit antibodies that were conjugated to horseradish peroxidase were used as secondary antibodies. For western blotting, 20 μg of proteins for each sample were denatured in 6 × SDS-PAGE sampling buffer by boiling in a water bath for 5 min, then separated by 12 % SDS-PAGE along with a pre-stained protein marker (10–124 kDa) and transferred to a PVDF membrane. The signals were revealed with Lumi-Light western blotting substrate (Roche, Mannheim, Germany) and captured on autoradiographic films (Kodak, New York, USA). Films were scanned using UMAX PowerLook 2100XL scanner and densitometric analysis of the bands was performed using ImageMaster Platinum software. The experiments were repeated for three times using different ovule attached with fiber samples.

### Bioinformatics analyses of the differentially displayed protein spots

A complete linkage hierarchical cluster analysis of the differentially displayed protein (DDP) spots was performed through a comparison of the average fold change value of each spots (comparison to 1 DPA) using MeV 4.7 software package. Functional classification of the DDP spots were performed using agriGO toolkits (http://bioinfo.cau.edu.cn/agriGO/) in hypergeometric testing mode. Amino acid sequences of the DDP spots were BLAST searched against with known DDPs of allotetraploid elongating cotton fibers to retrieve the most homologous allotetraploid cotton DDPs with an E-value = 0.

## Additional files

Additional file 1: Figure S1. Fiber length of two cotton species in the early stages of fiber elongation process. (TIF 44 kb) Additional file 2: Table S1. Relative abundance of the 55 DDP spots. (XLSX 19 kb) Additional file 3: Table S2. Information of the identified 55 DDP spots. (XLSX 16 kb) Additional file 4: Annotated PMF spectra for 55 DDP spots. (PPT 728 kb)

## Abbreviations

2-DE: Two-dimensional electrophoresis
ChSOD: Chloroplast Cu/Zn superoxide dismutase
DDP: Differentially displayed protein
DPA: Days post-anthesis
EST: Expressed sequence tag
HSP70: Heat shock protein 70
MALDI TOF: Matrix-assisted laser desorption/ionization time of flight
PMF: Peptide mass fingerprinting
