# Supplementary figures and images for: Comparative proteomic analyses of Asian cotton ovules with attached fibers in the early stages of fiber elongation process
Source: Proteome Sci. 2016 Sep 8;14(1):13. doi: 10.1186/s12953-016-0101-1 (PMC5015342; doi:10.1186/s12953-016-0101-1)

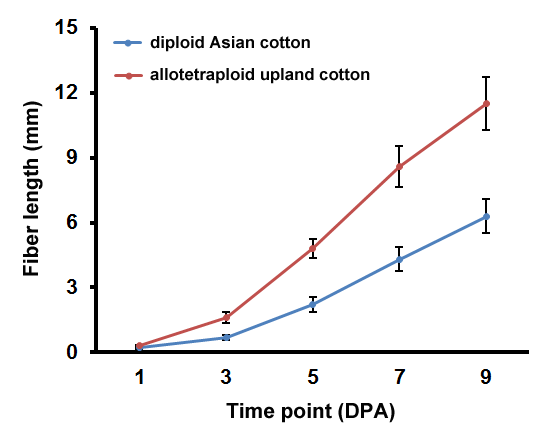

Supplement: Additional file 1: Figure S1. — Fiber length of two cotton species in the early stages of fiber elongation process. (TIF 44 kb) [file 12953_2016_101_MOESM1_ESM.tif]
