# Supplementary material for: Comparative proteomic analyses of Asian cotton ovules with attached fibers in the early stages of fiber elongation process
Source: Proteome Sci. 2016 Sep 8;14(1):13. doi: 10.1186/s12953-016-0101-1 (PMC5015342; doi:10.1186/s12953-016-0101-1)
Supplement: Additional file 4: — Annotated PMF spectra for 55 DDP spots. (PPT 728 kb) [file 12953_2016_101_MOESM4_ESM.ppt]

## Slide 1
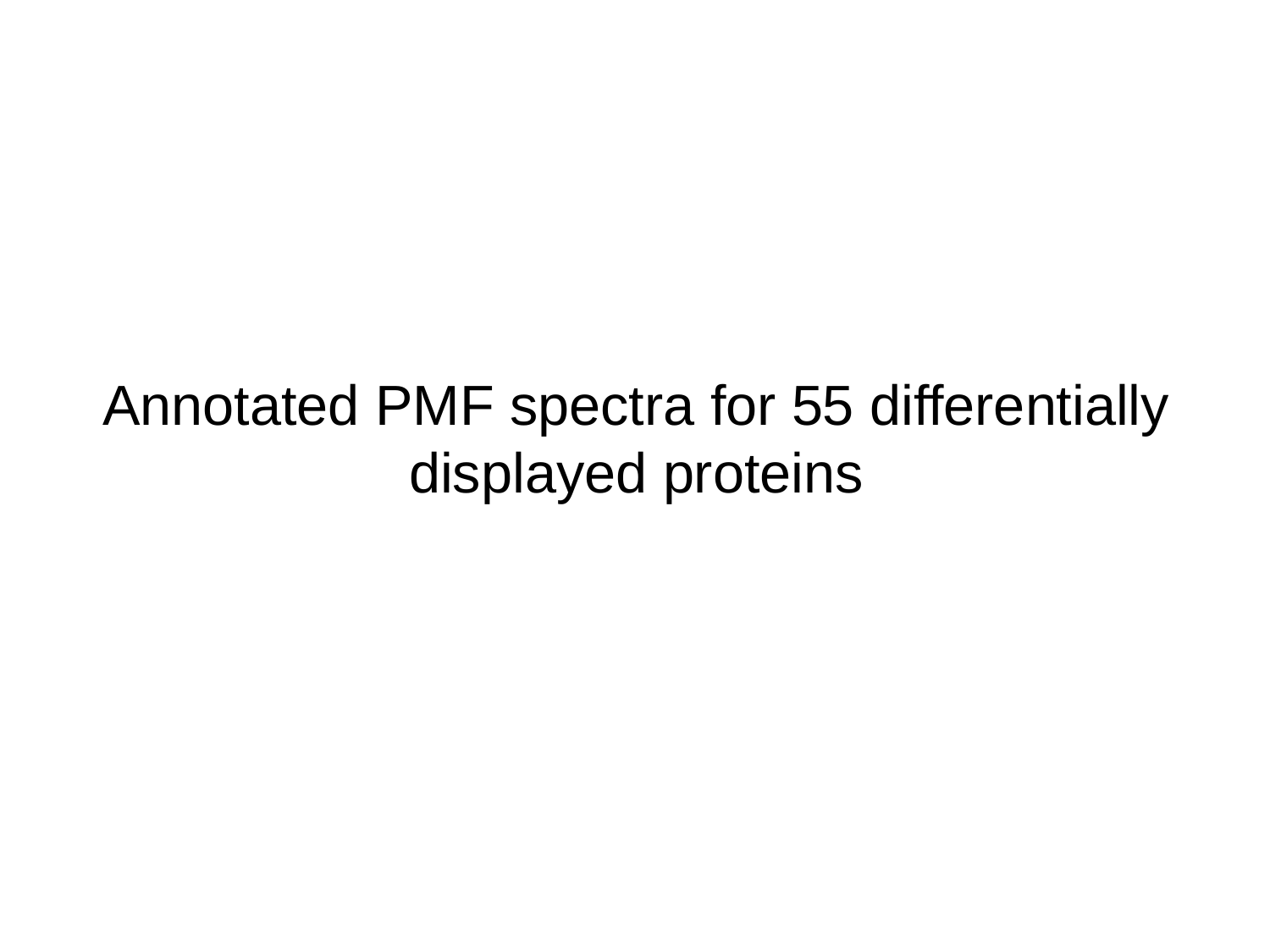

# Annotated PMF spectra for 55 differentially displayed proteins

## Slide 2
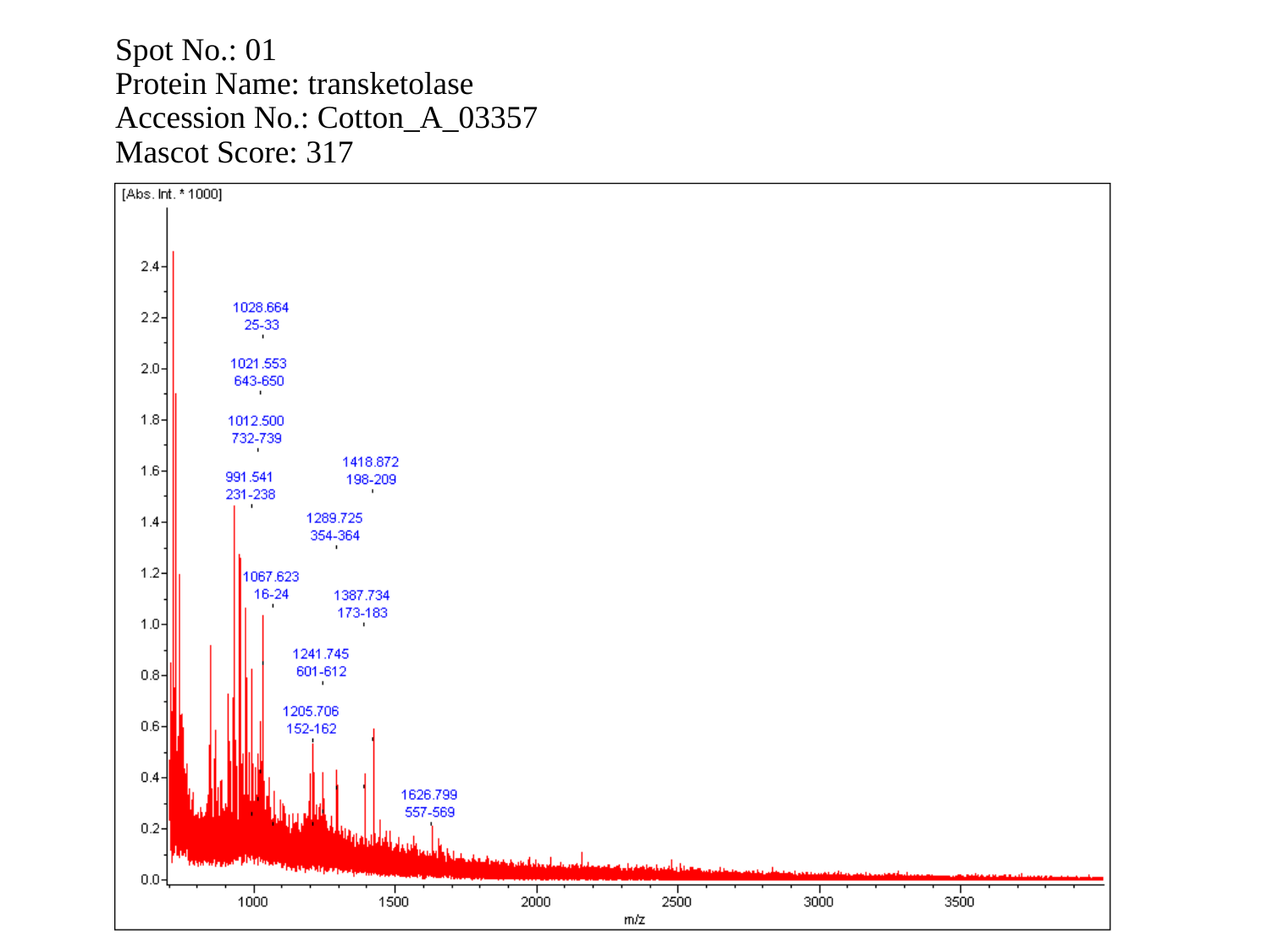

Spot No.: 01
Protein Name: transketolase
Accession No.: Cotton_A_03357
Mascot Score: 317

## Slide 3
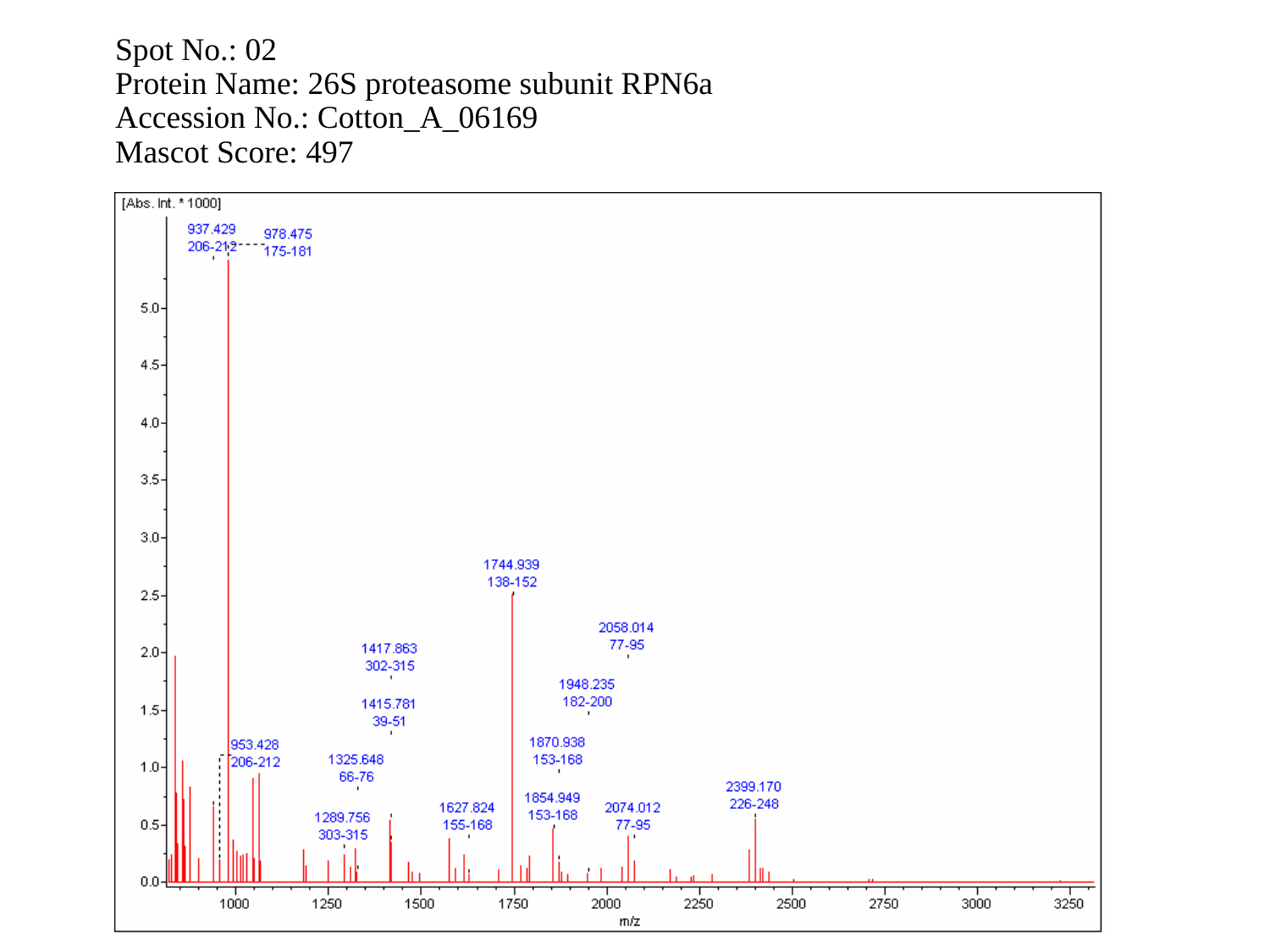

Spot No.: 02
Protein Name: 26S proteasome subunit RPN6a
Accession No.: Cotton_A_06169
Mascot Score: 497

## Slide 4
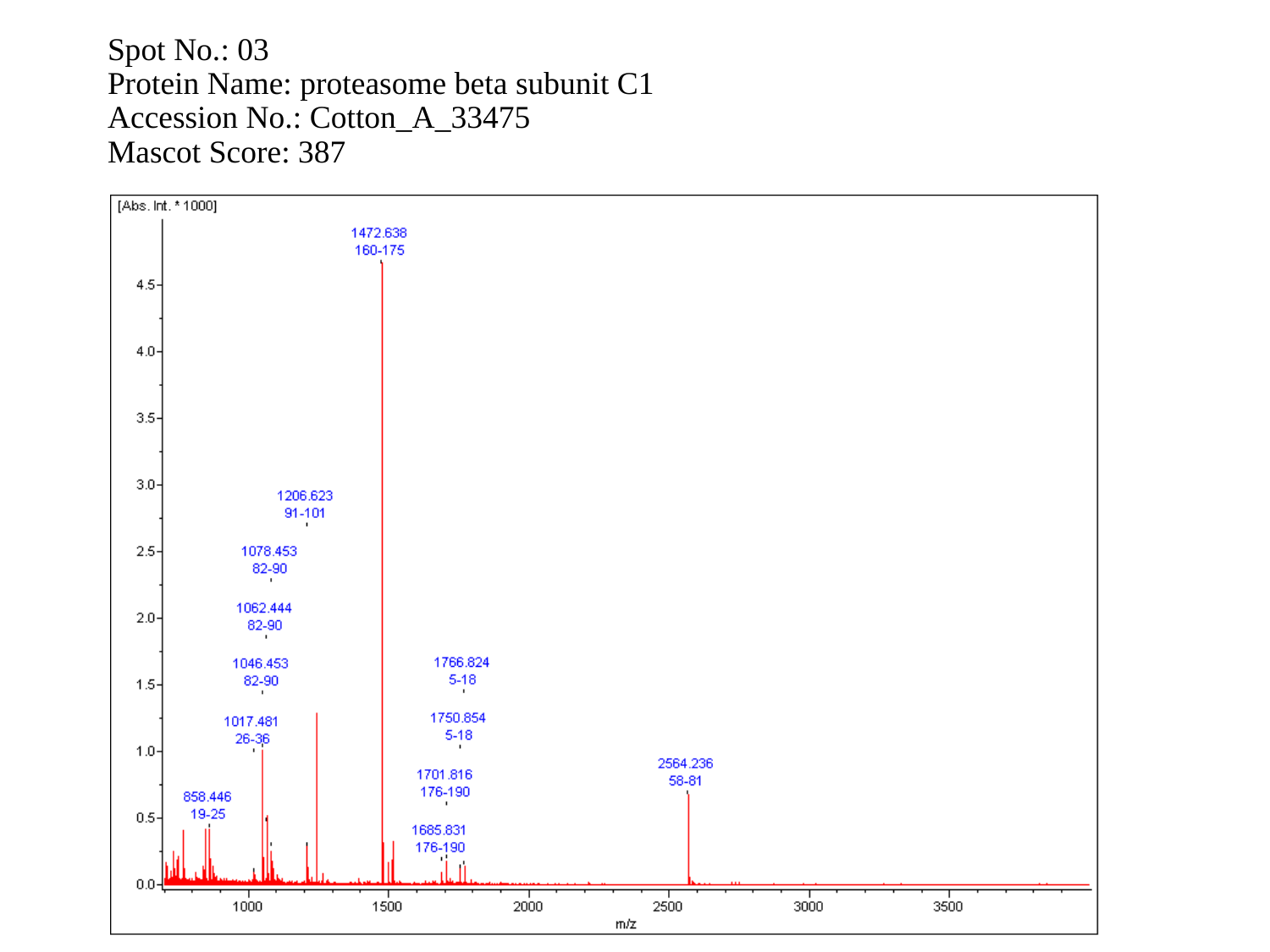

Spot No.: 03
Protein Name: proteasome beta subunit C1
Accession No.: Cotton_A_33475
Mascot Score: 387

## Slide 5
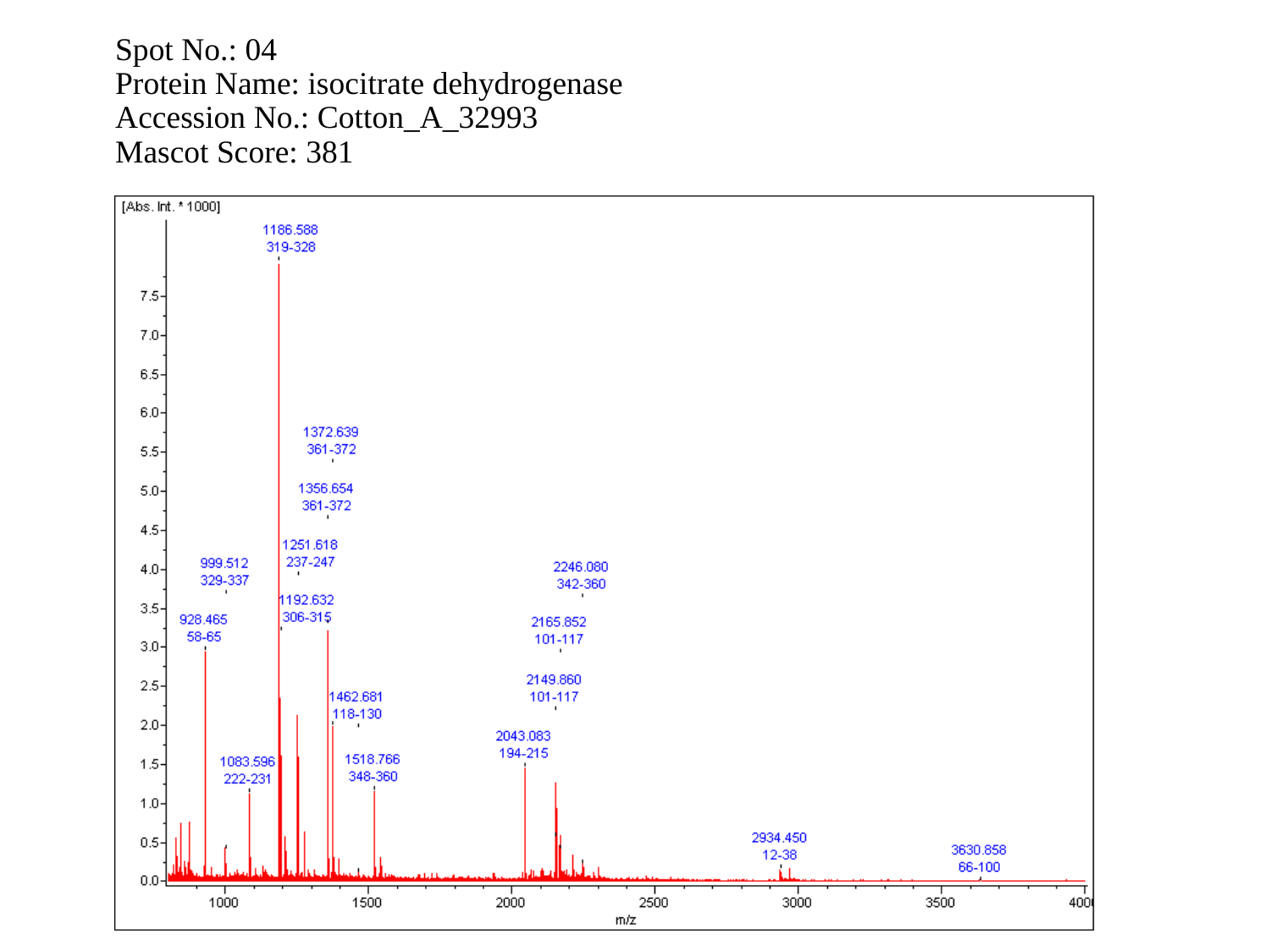

Spot No.: 04
Protein Name: isocitrate dehydrogenase
Accession No.: Cotton_A_32993
Mascot Score: 381

## Slide 6
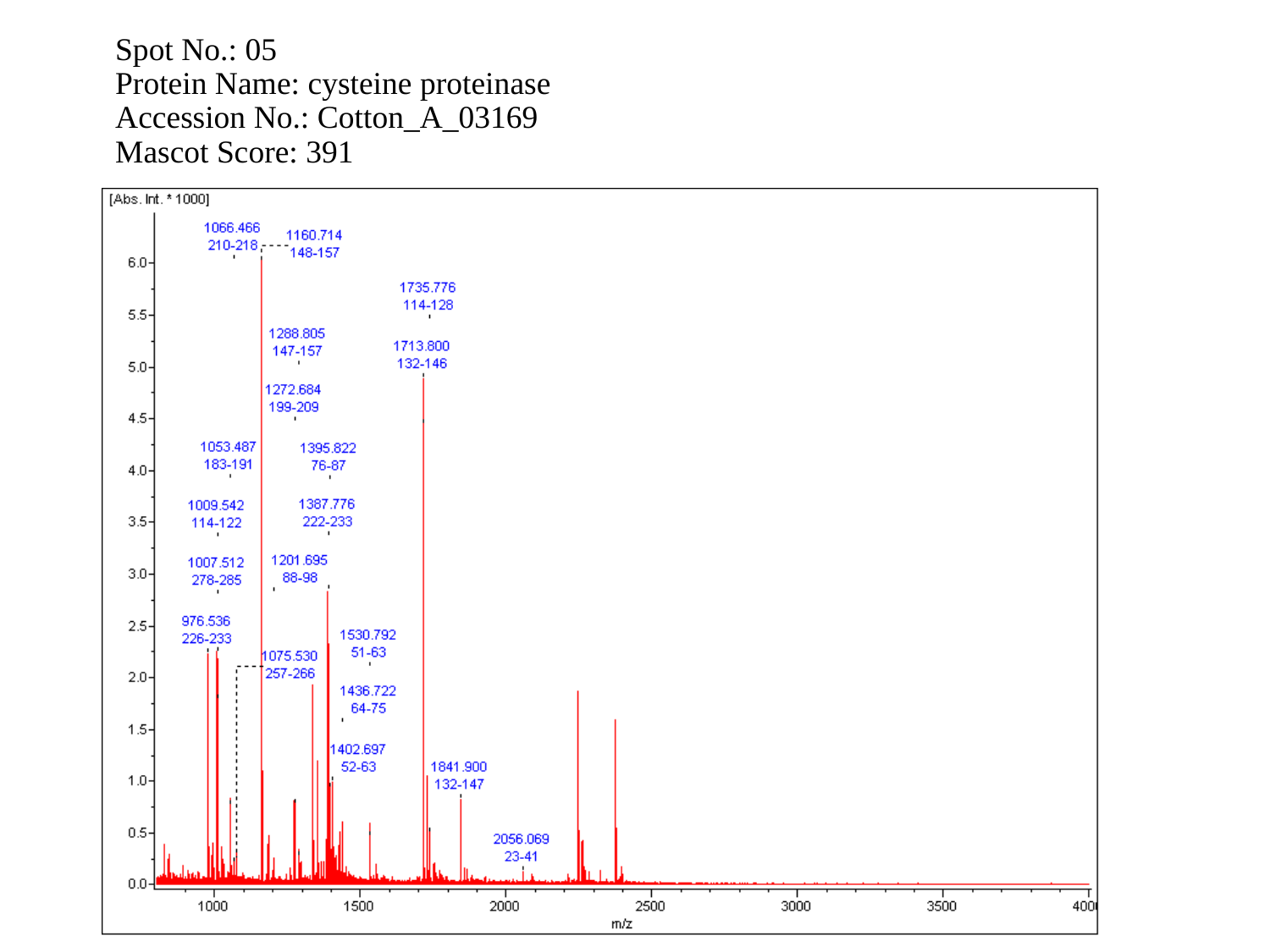

Spot No.: 05
Protein Name: cysteine proteinase
Accession No.: Cotton_A_03169
Mascot Score: 391

## Slide 7
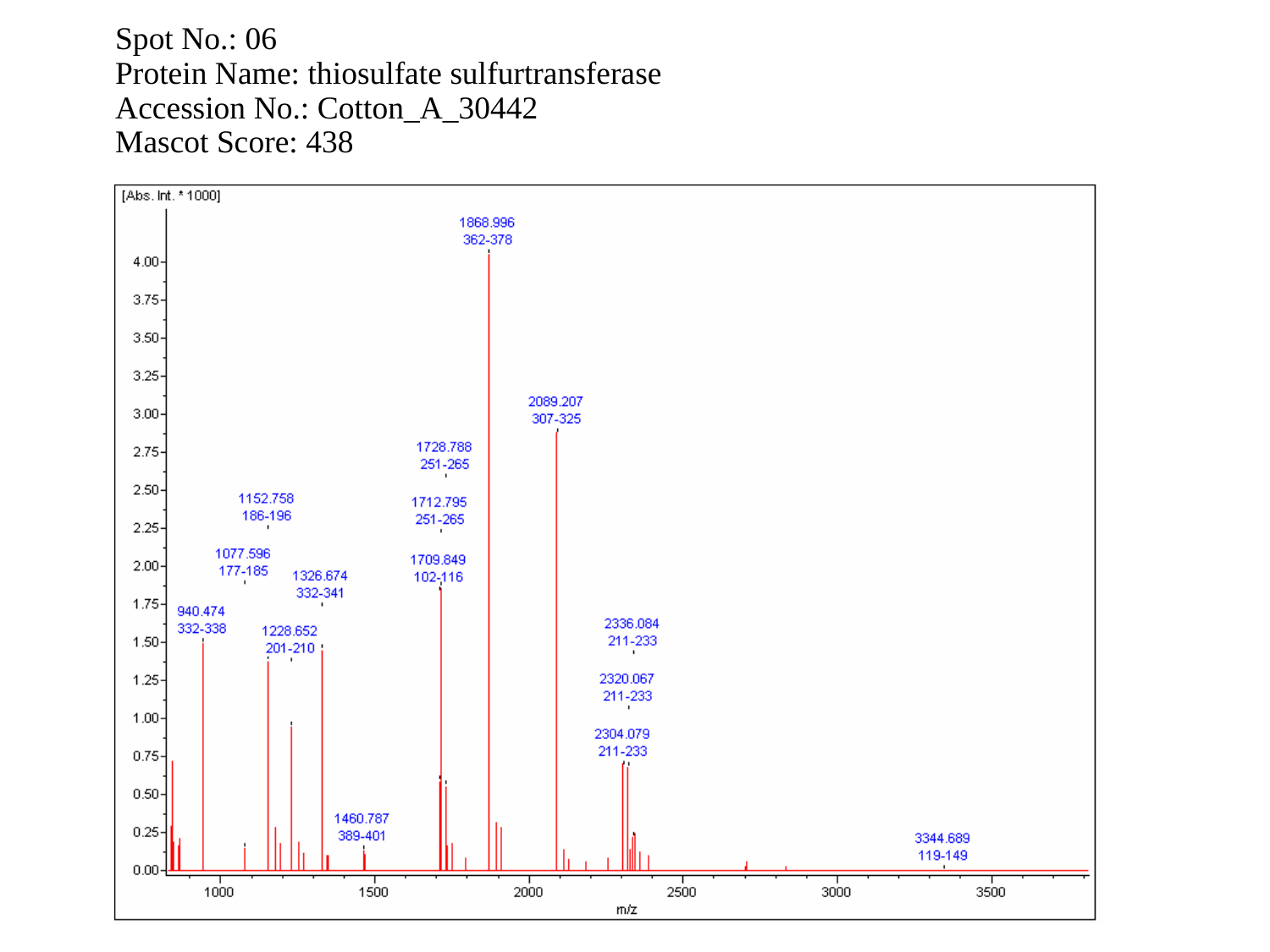

Spot No.: 06
Protein Name: thiosulfate sulfurtransferase Accession No.: Cotton_A_30442
Mascot Score: 438

## Slide 8
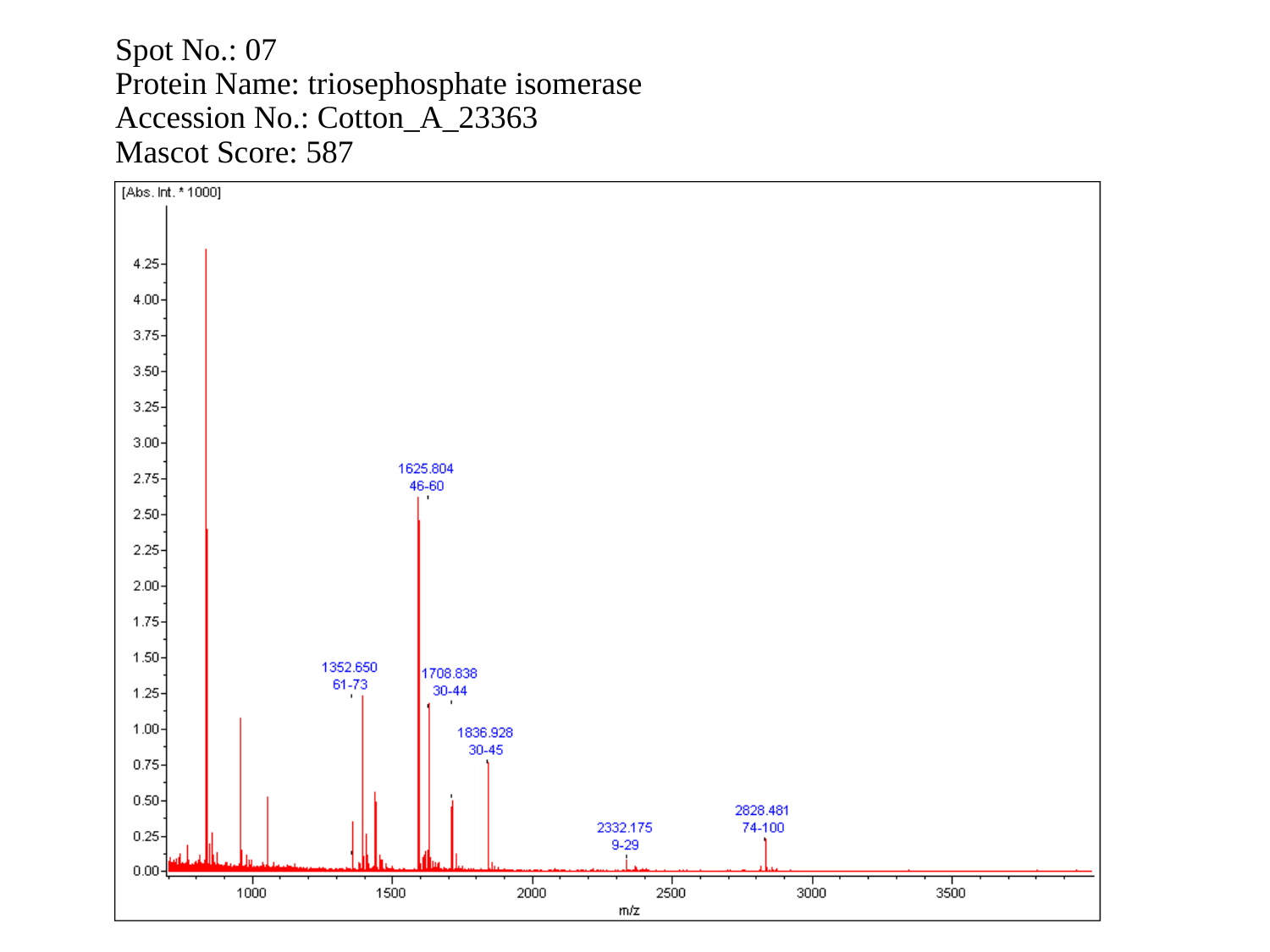

Spot No.: 07
Protein Name: triosephosphate isomerase
Accession No.: Cotton_A_23363
Mascot Score: 587

## Slide 9
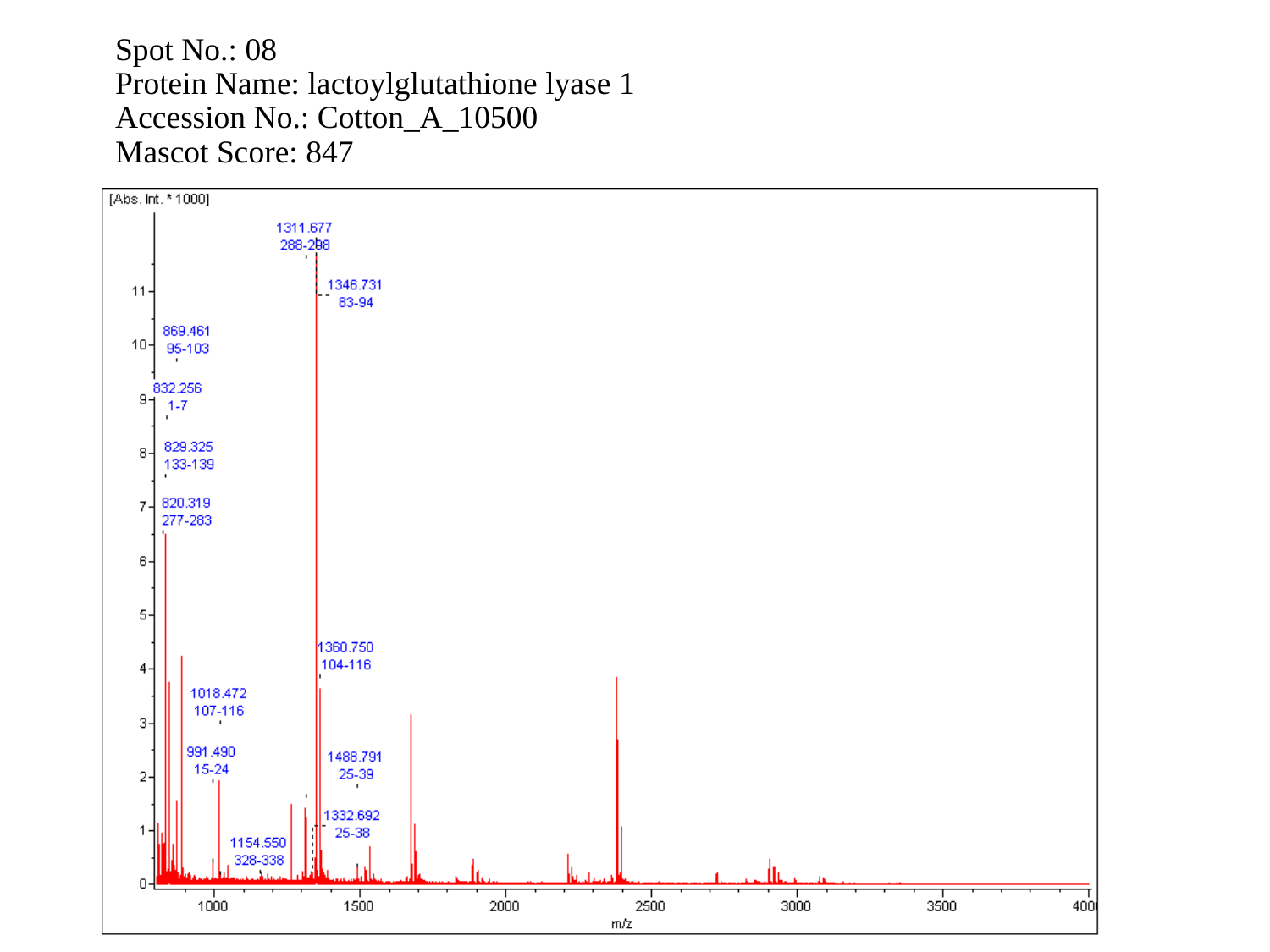

Spot No.: 08
Protein Name: lactoylglutathione lyase 1
Accession No.: Cotton_A_10500
Mascot Score: 847

## Slide 10
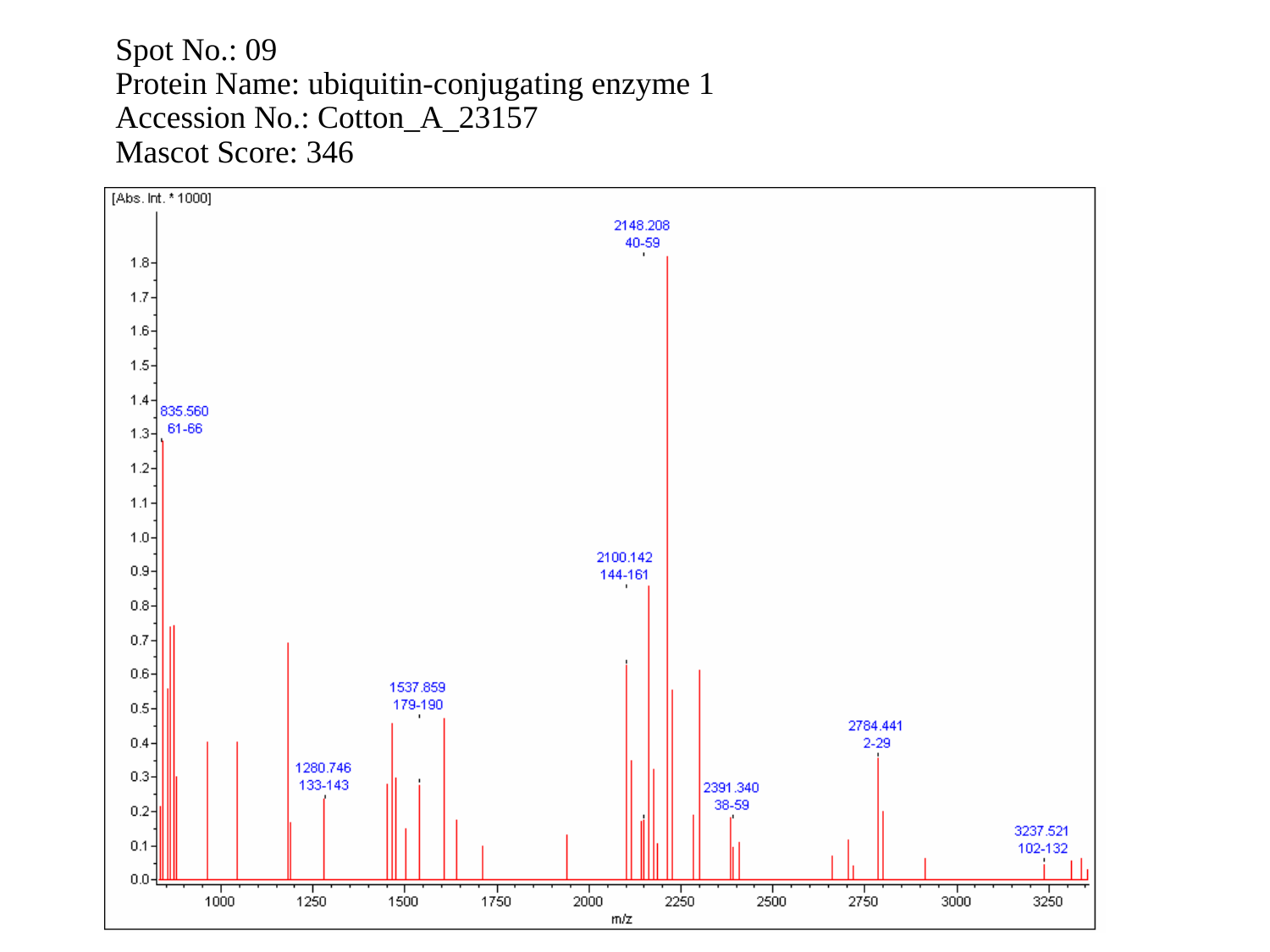

Spot No.: 09
Protein Name: ubiquitin-conjugating enzyme 1
Accession No.: Cotton_A_23157
Mascot Score: 346

## Slide 11
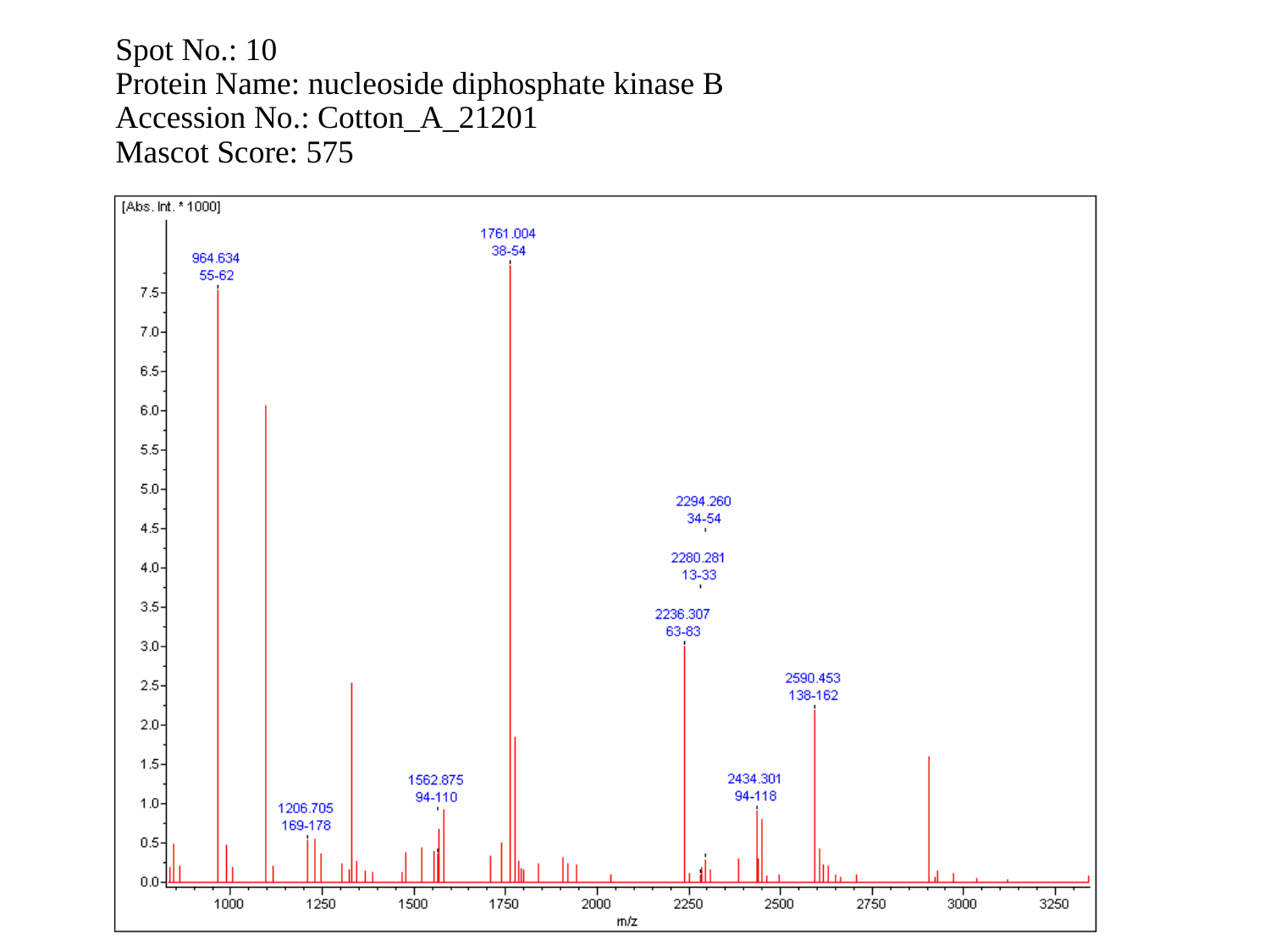

Spot No.: 10
Protein Name: nucleoside diphosphate kinase B
Accession No.: Cotton_A_21201
Mascot Score: 575

## Slide 12
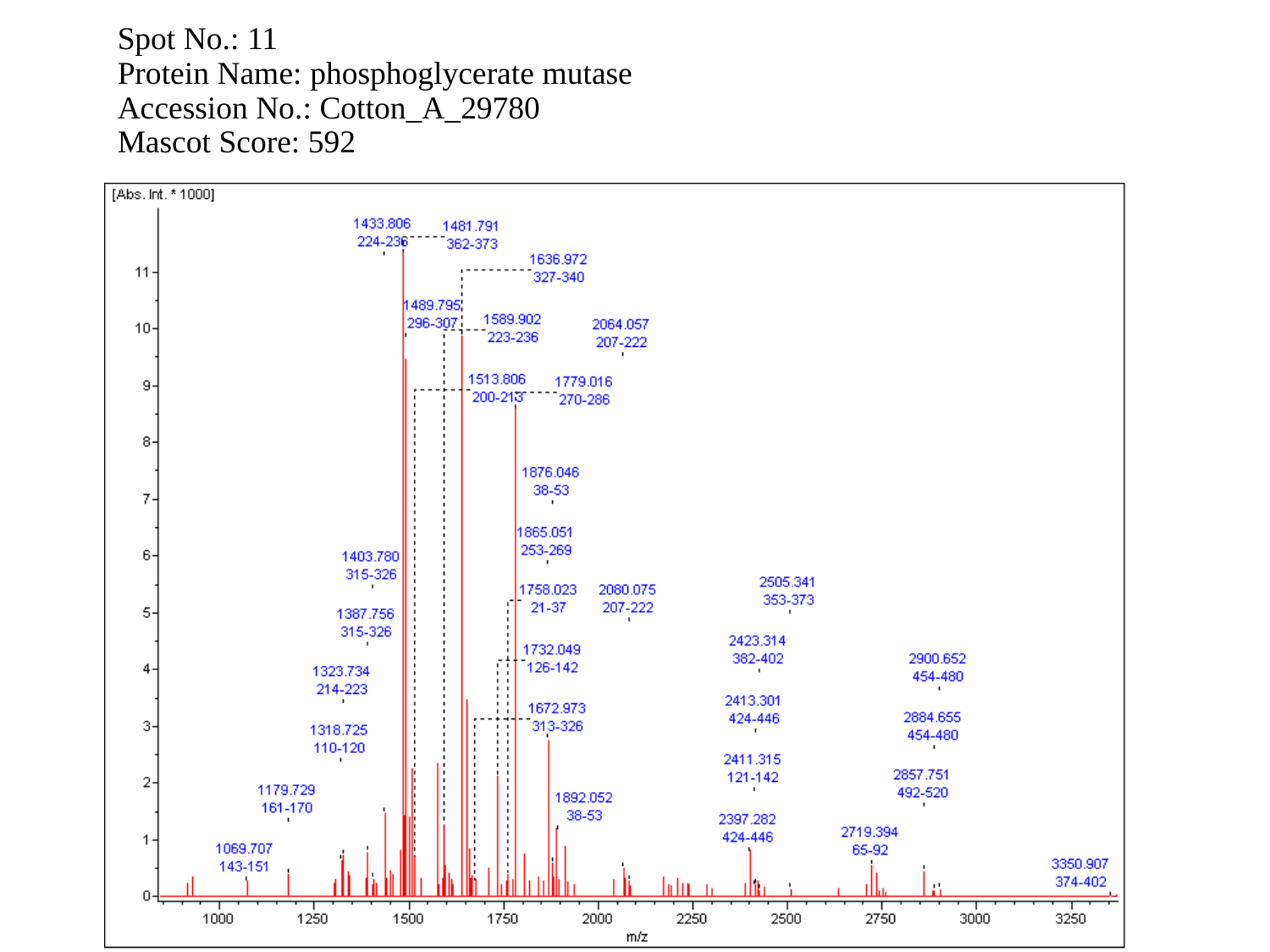

Spot No.: 11
Protein Name: phosphoglycerate mutase
Accession No.: Cotton_A_29780
Mascot Score: 592

## Slide 13
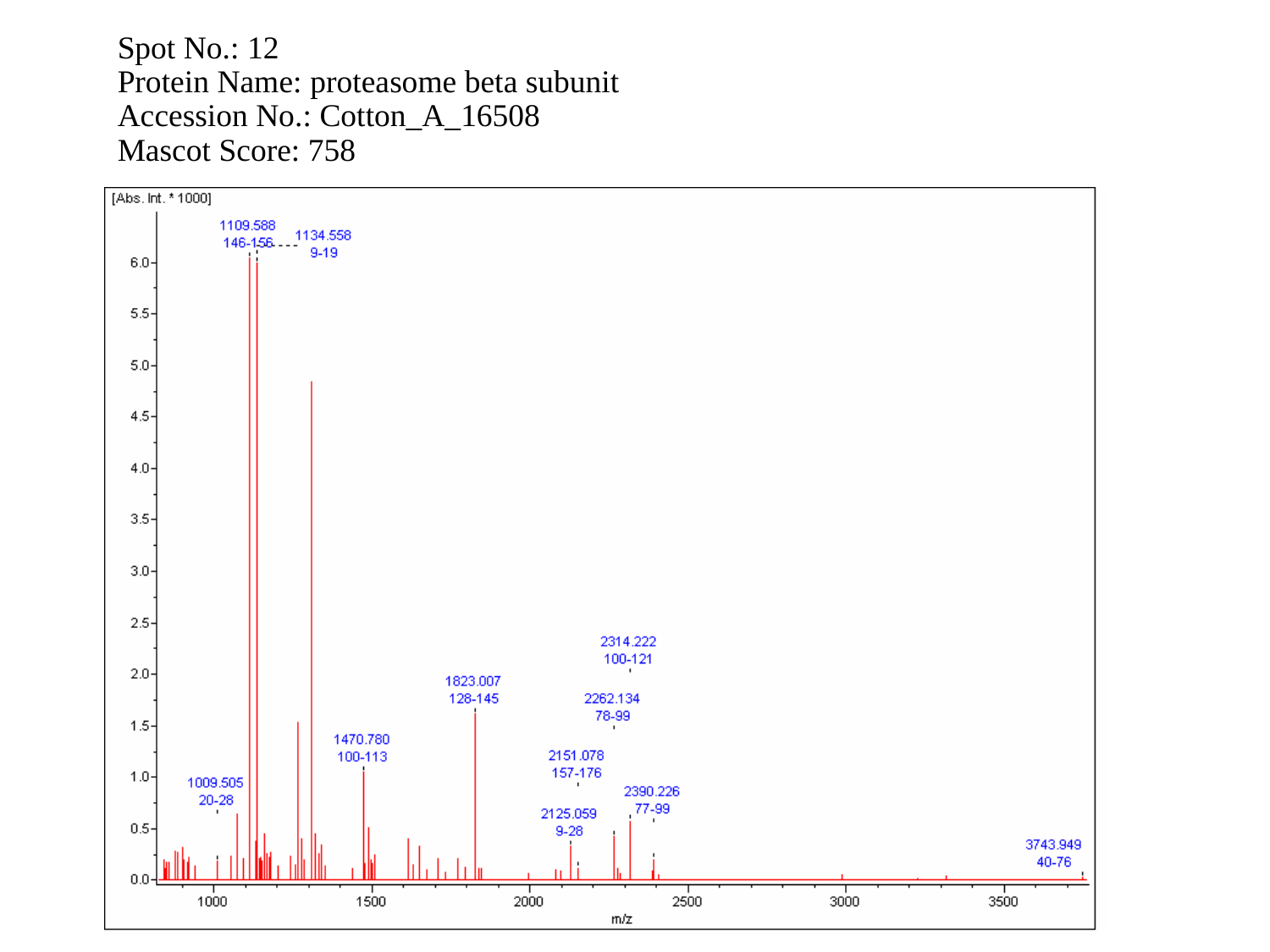

Spot No.: 12
Protein Name: proteasome beta subunit
Accession No.: Cotton_A_16508
Mascot Score: 758

## Slide 14
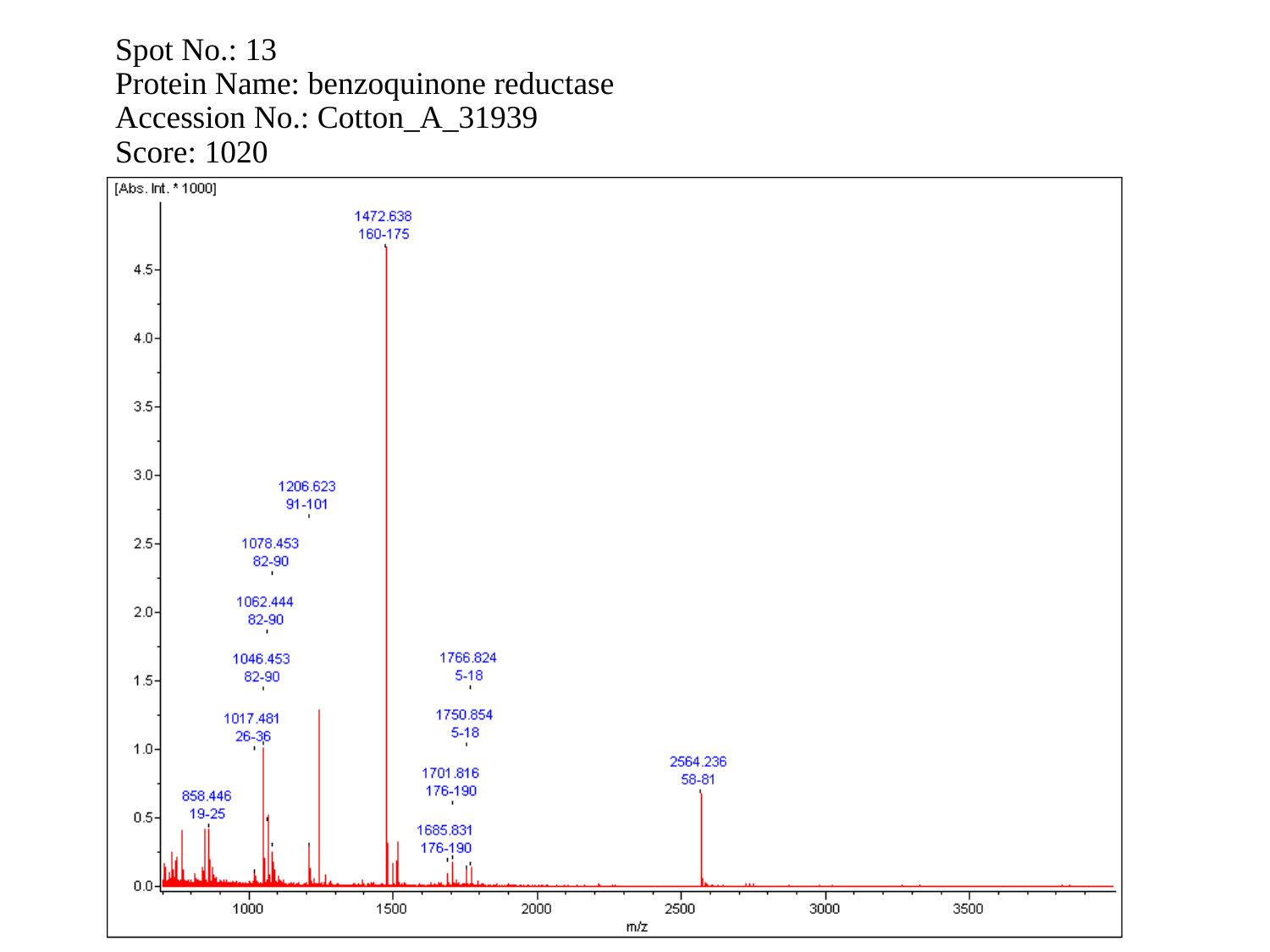

Spot No.: 13
Protein Name: benzoquinone reductase
Accession No.: Cotton_A_31939
Score: 1020

## Slide 15
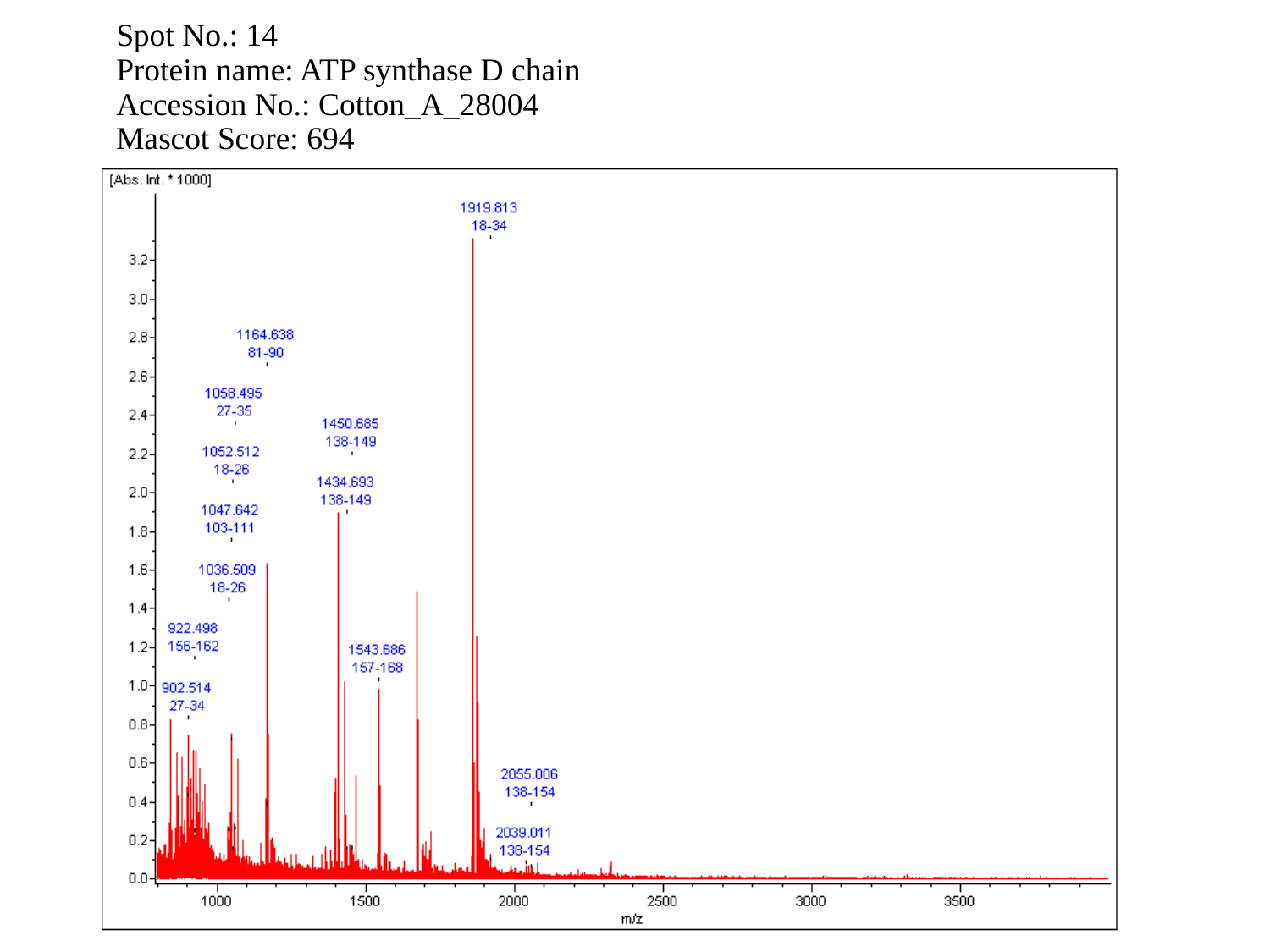

Spot No.: 14
Protein name: ATP synthase D chain
Accession No.: Cotton_A_28004
Mascot Score: 694

## Slide 16
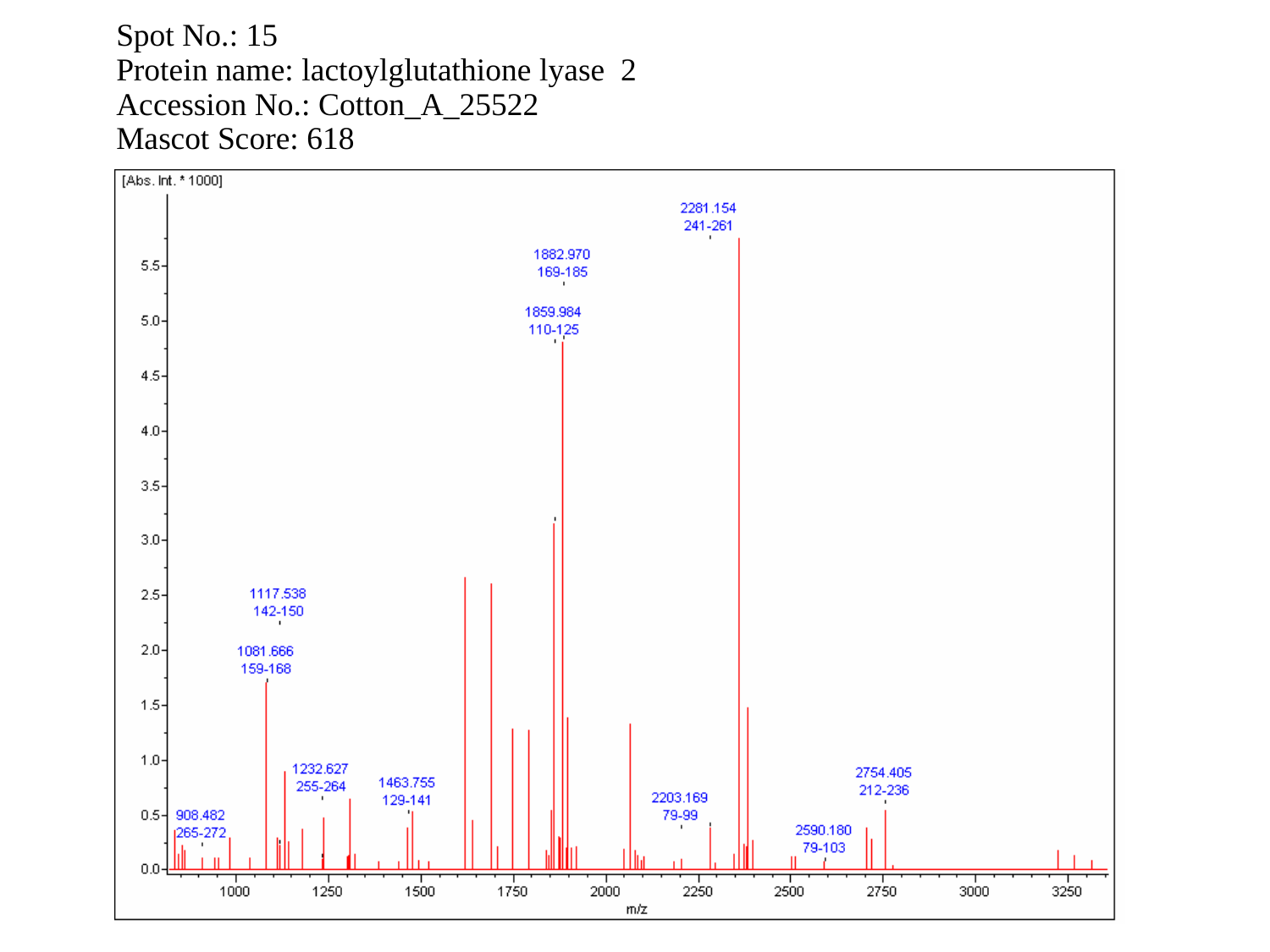

Spot No.: 15
Protein name: lactoylglutathione lyase 2
Accession No.: Cotton_A_25522
Mascot Score: 618

## Slide 17
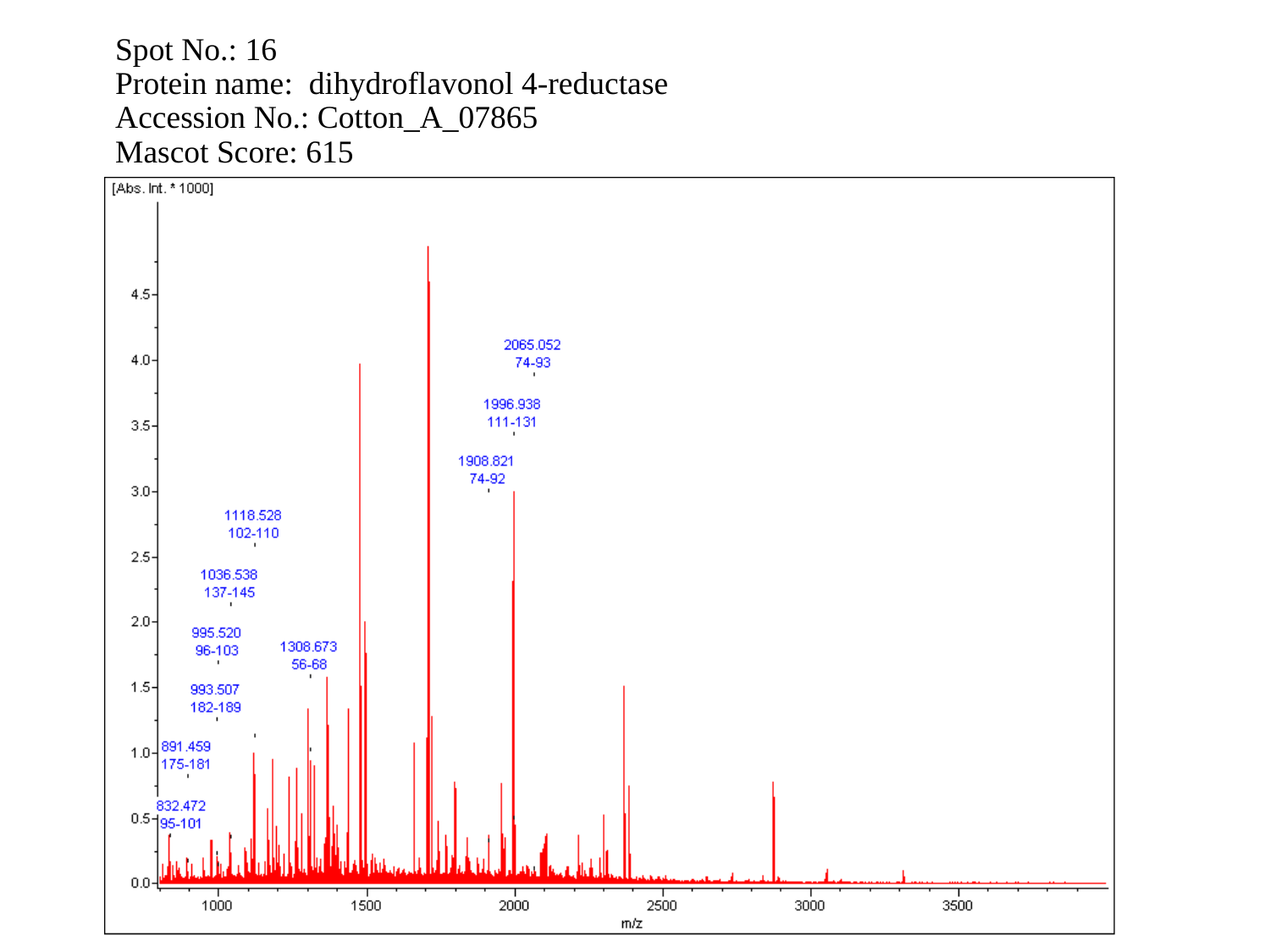

Spot No.: 16
Protein name: dihydroflavonol 4-reductase
Accession No.: Cotton_A_07865
Mascot Score: 615

## Slide 18
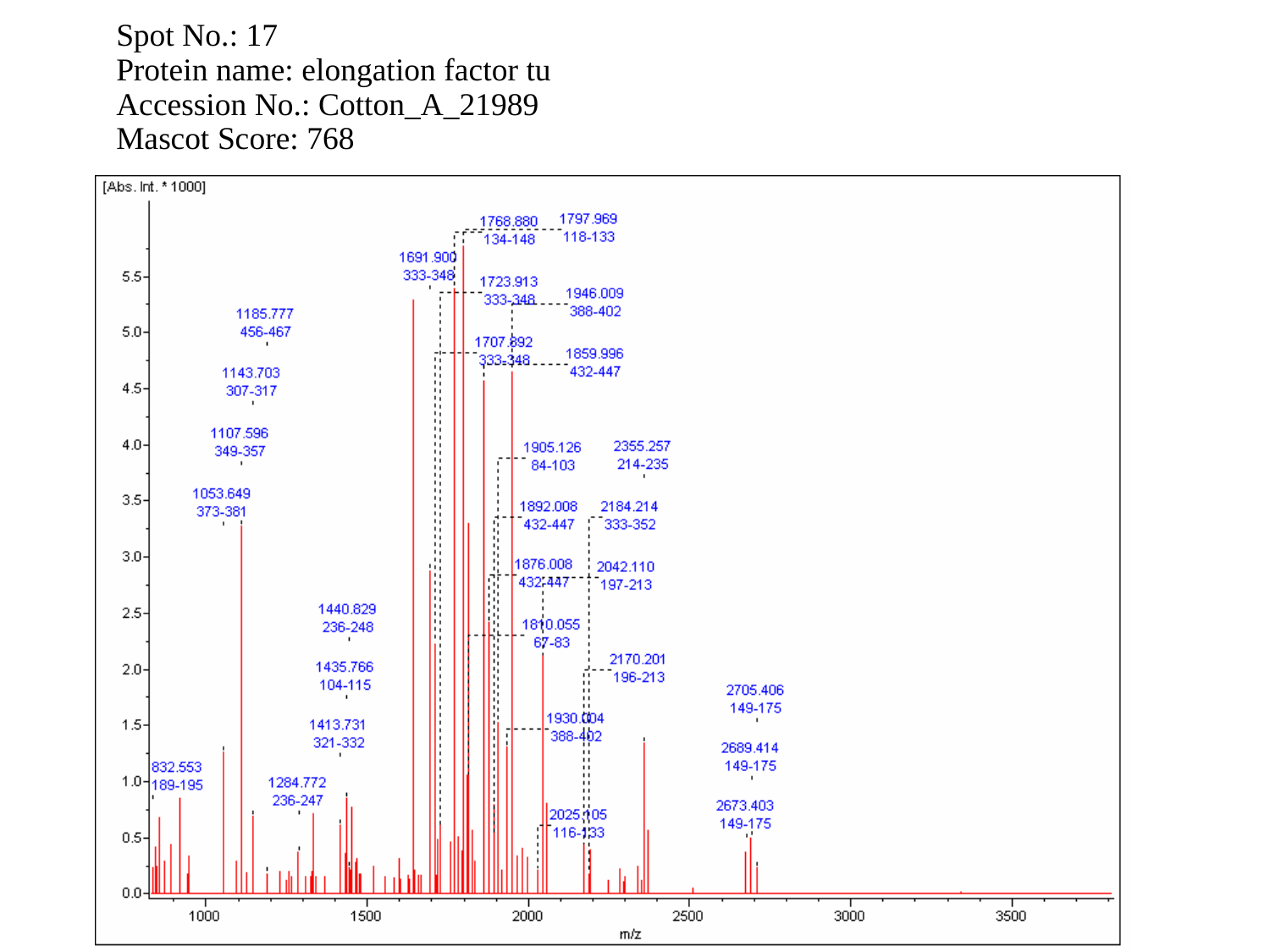

Spot No.: 17
Protein name: elongation factor tu
Accession No.: Cotton_A_21989
Mascot Score: 768

## Slide 19
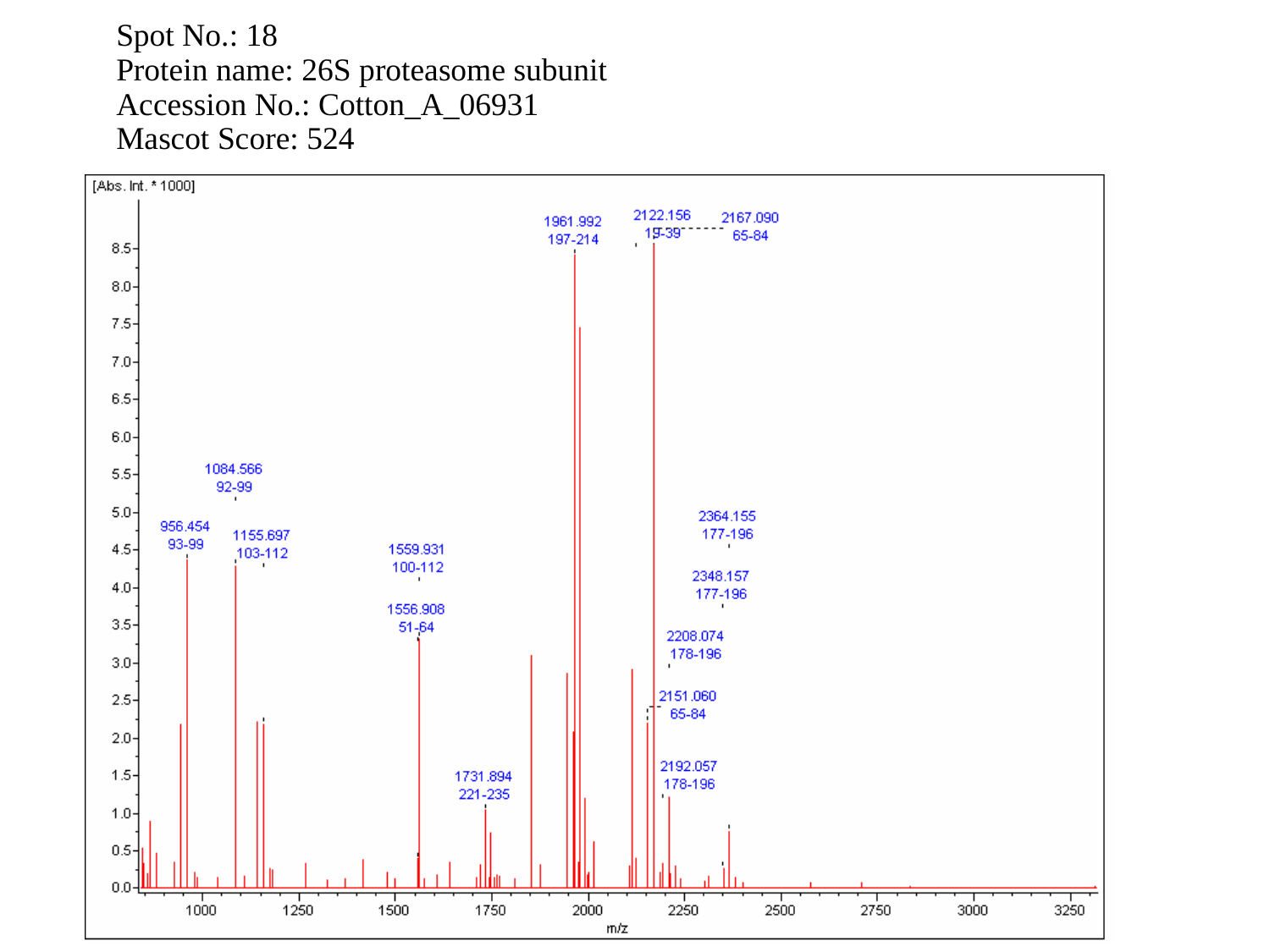

Spot No.: 18
Protein name: 26S proteasome subunit
Accession No.: Cotton_A_06931
Mascot Score: 524

## Slide 20
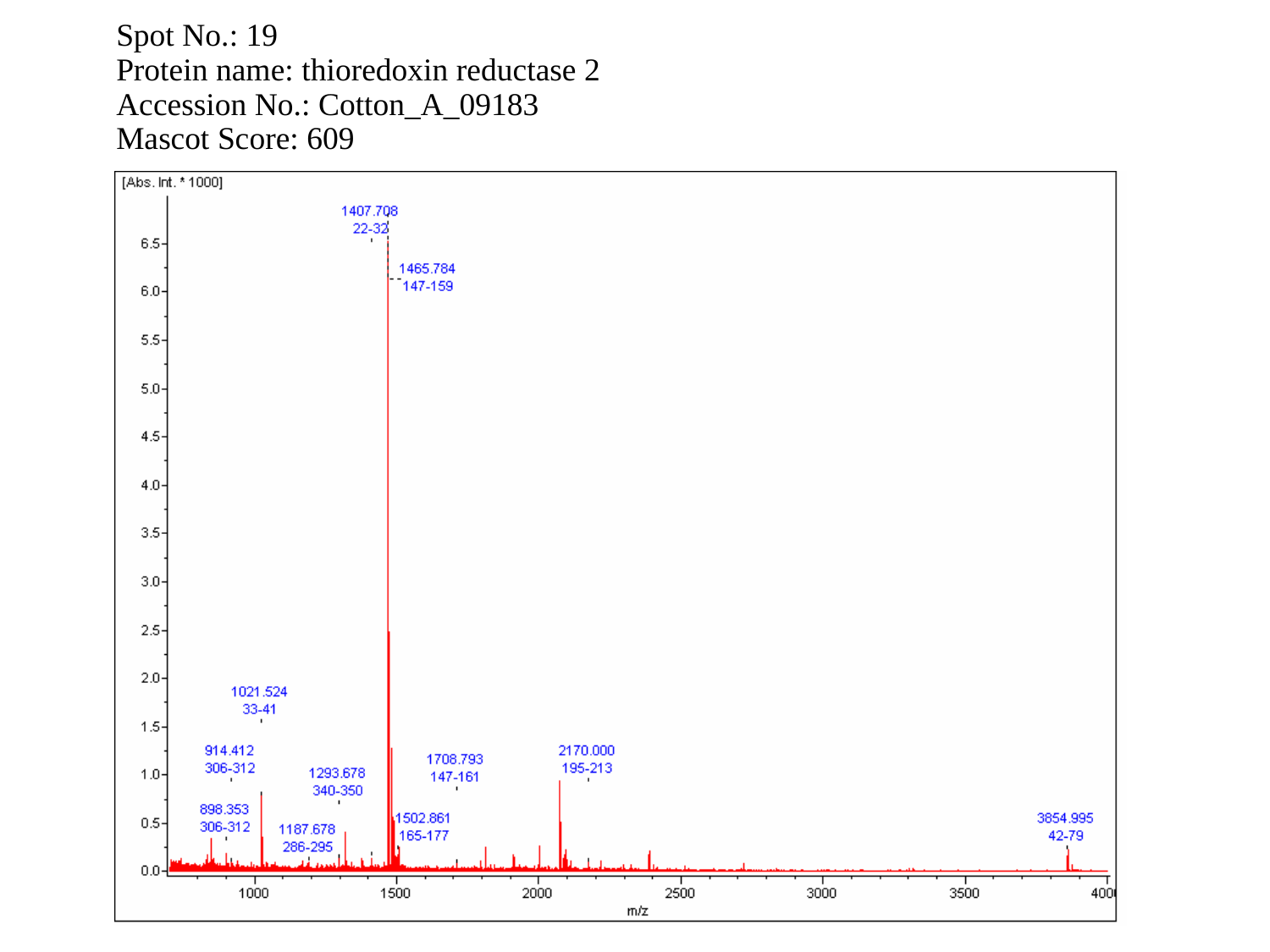

Spot No.: 19
Protein name: thioredoxin reductase 2
Accession No.: Cotton_A_09183
Mascot Score: 609

## Slide 21
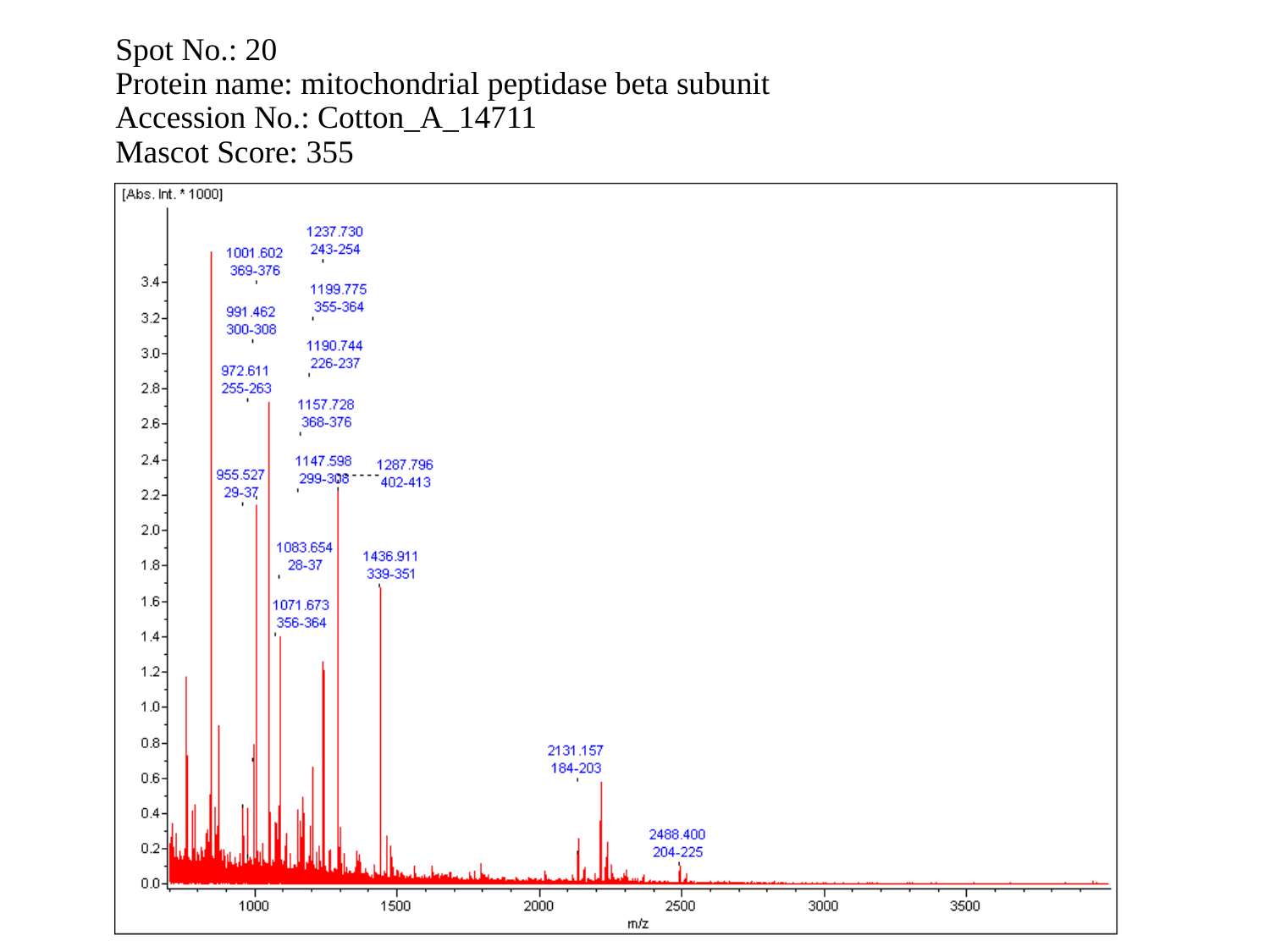

Spot No.: 20
Protein name: mitochondrial peptidase beta subunit
Accession No.: Cotton_A_14711
Mascot Score: 355

## Slide 22
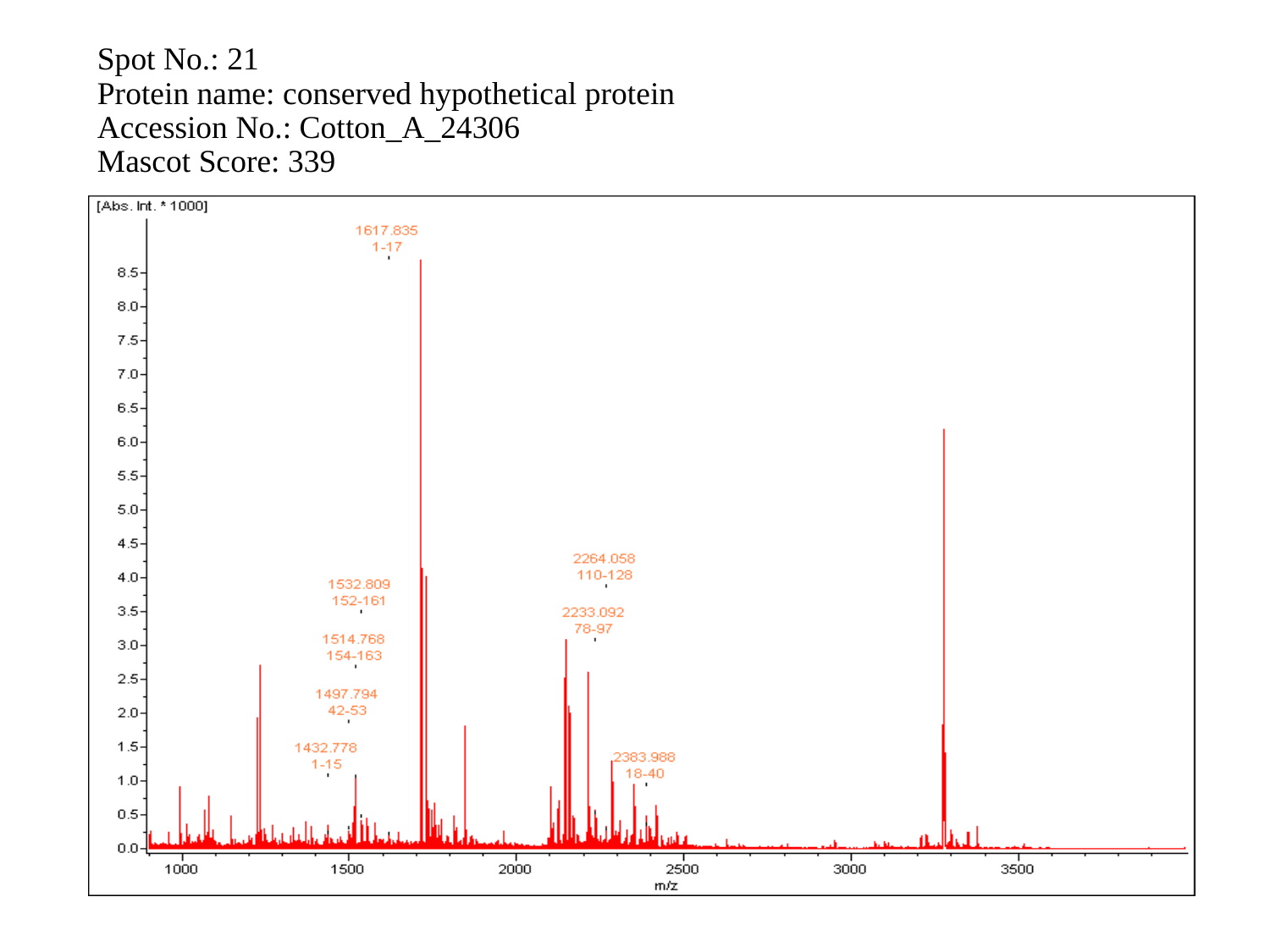

Spot No.: 21
Protein name: conserved hypothetical protein
Accession No.: Cotton_A_24306
Mascot Score: 339

## Slide 23
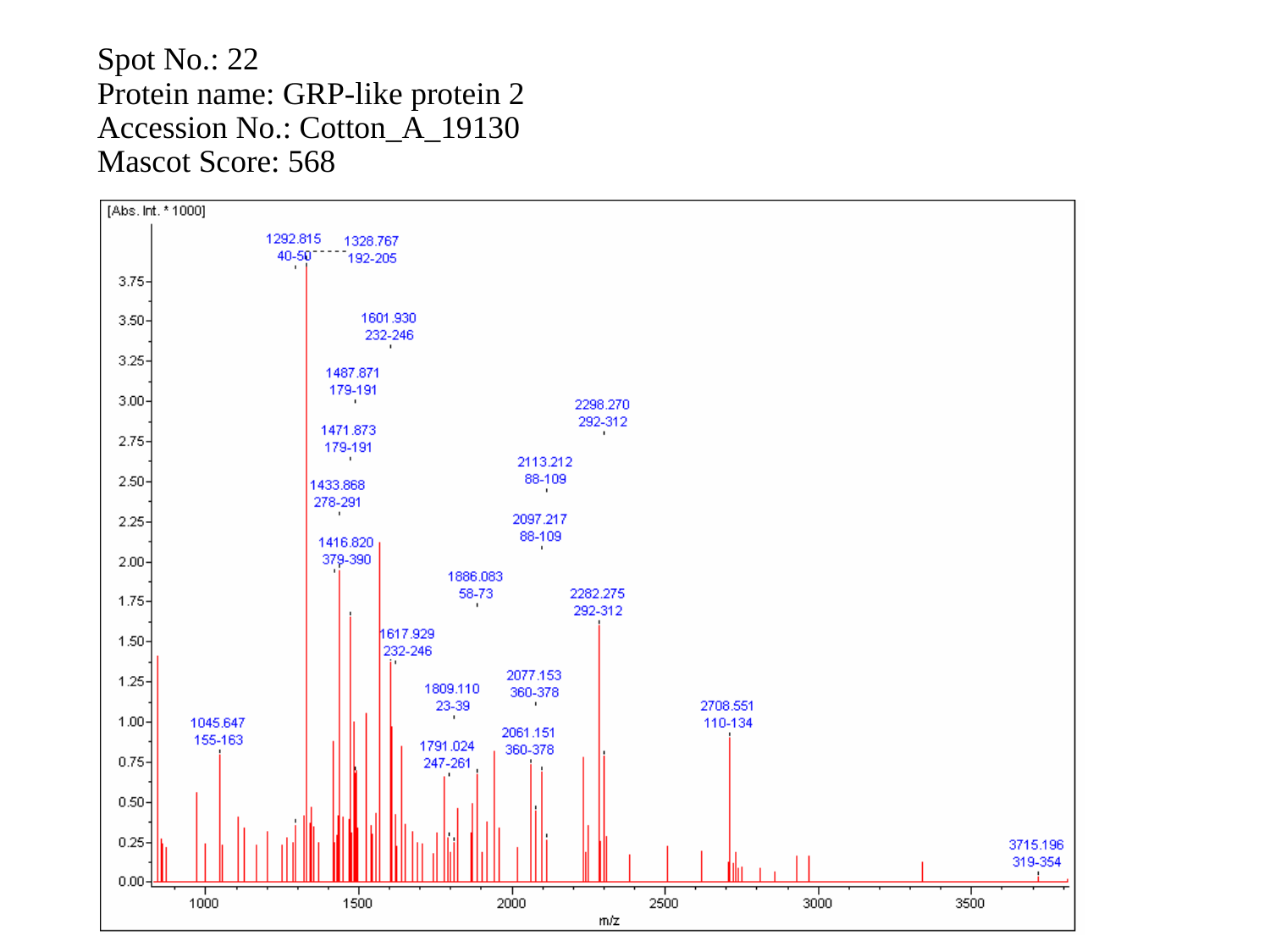

Spot No.: 22
Protein name: GRP-like protein 2
Accession No.: Cotton_A_19130
Mascot Score: 568

## Slide 24
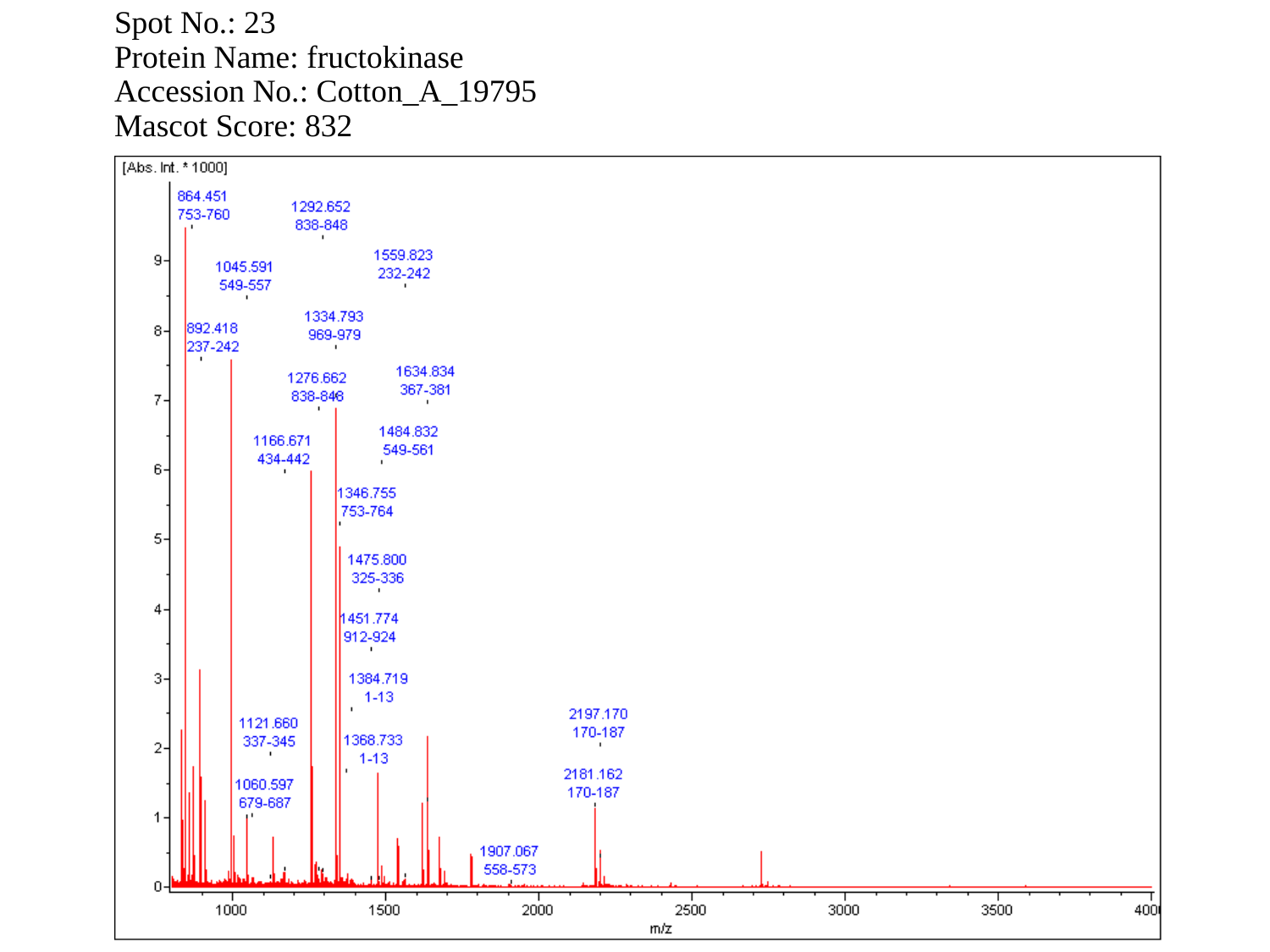

Spot No.: 23
Protein Name: fructokinase
Accession No.: Cotton_A_19795
Mascot Score: 832

## Slide 25
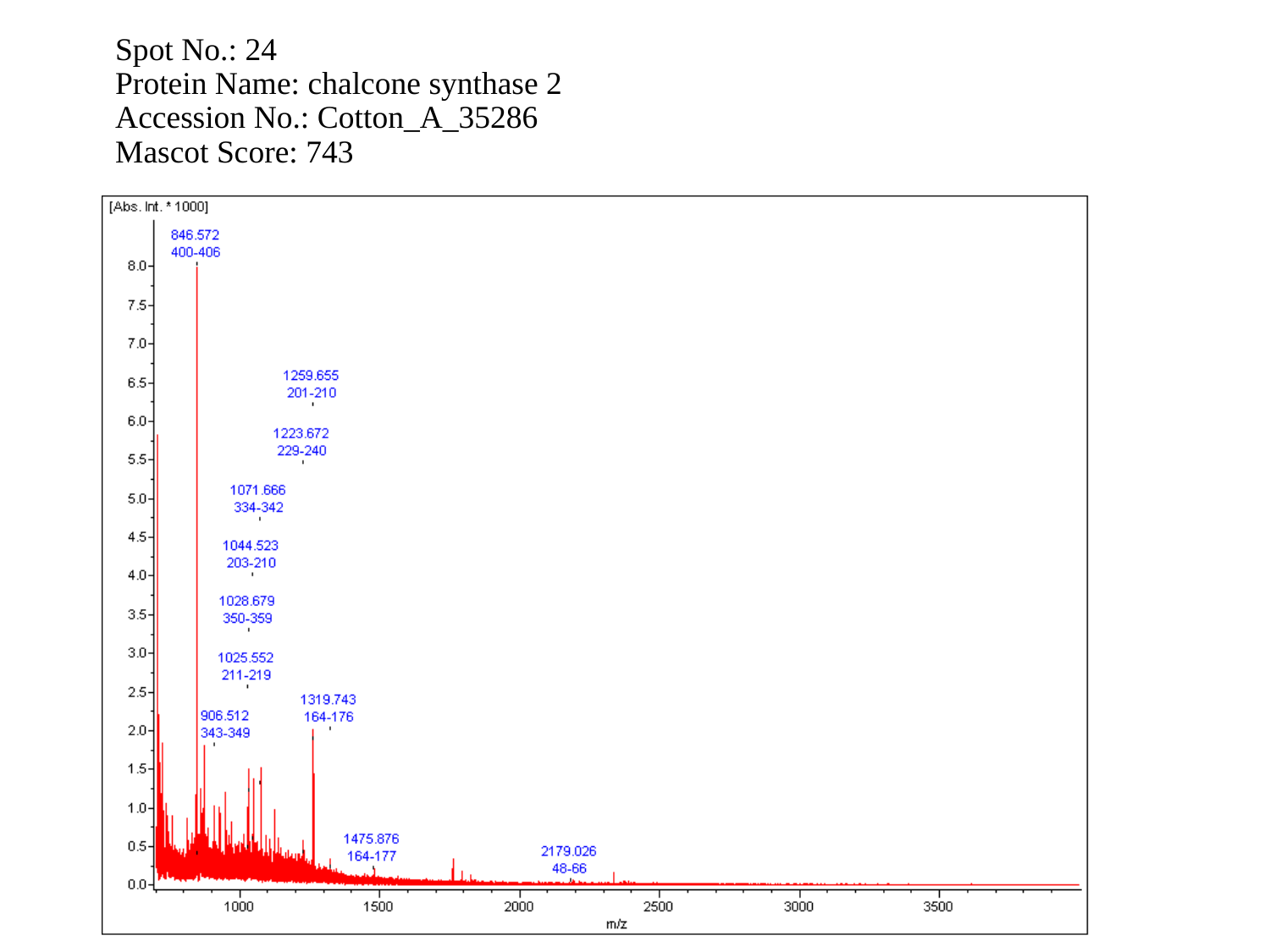

Spot No.: 24
Protein Name: chalcone synthase 2
Accession No.: Cotton_A_35286
Mascot Score: 743

## Slide 26
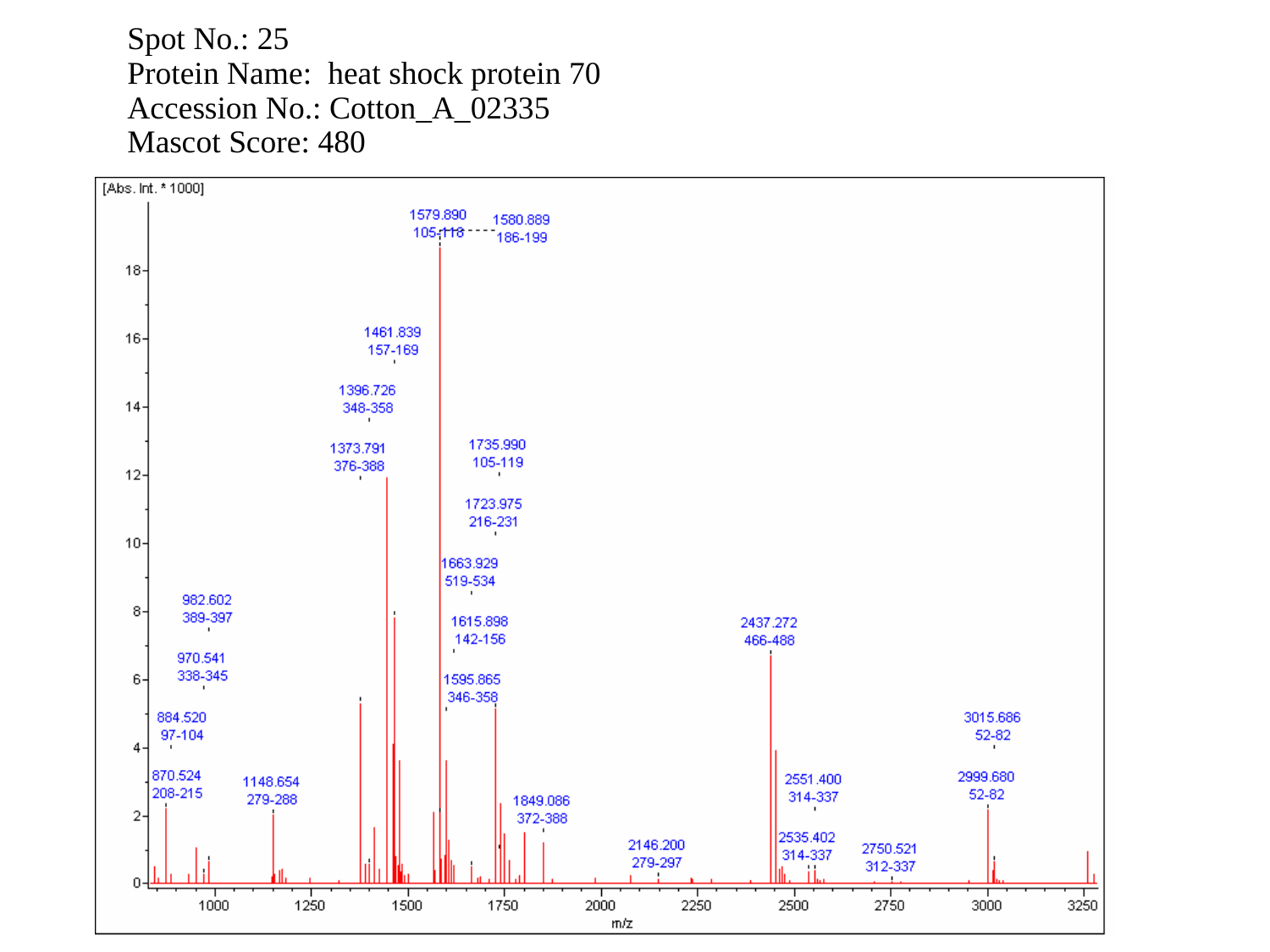

Spot No.: 25
Protein Name: heat shock protein 70
Accession No.: Cotton_A_02335
Mascot Score: 480

## Slide 27
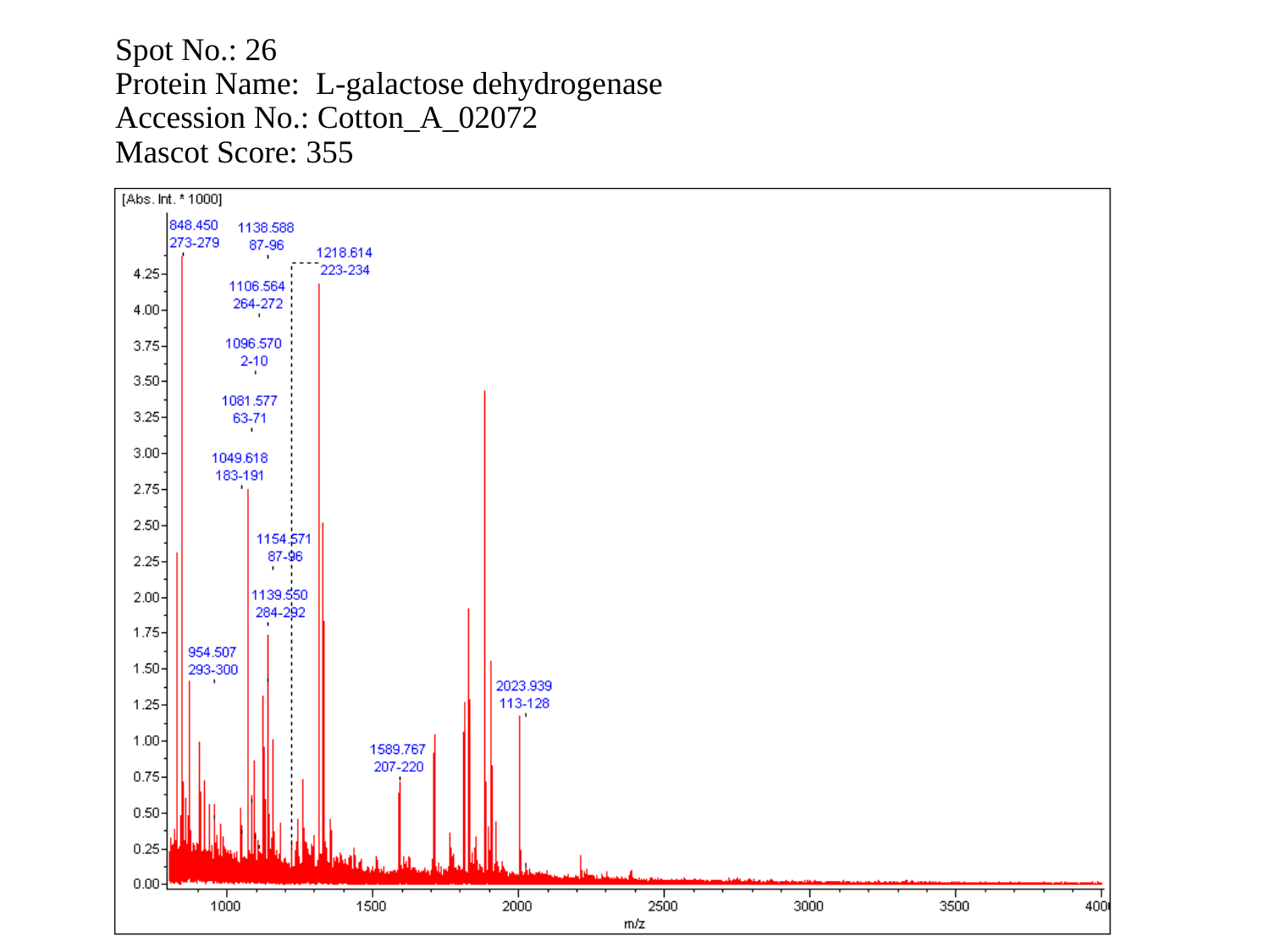

Spot No.: 26
Protein Name: L-galactose dehydrogenase
Accession No.: Cotton_A_02072
Mascot Score: 355

## Slide 28
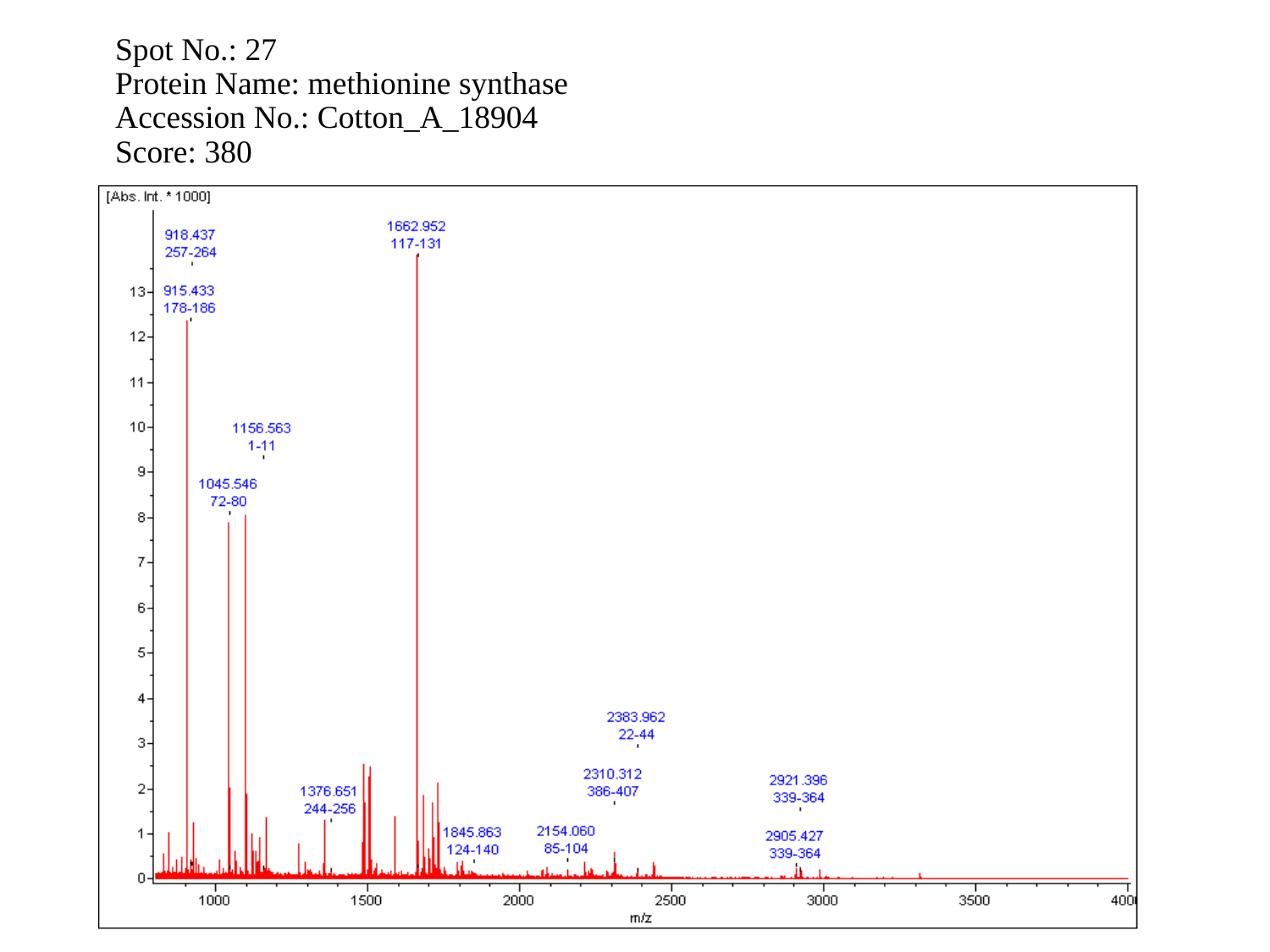

Spot No.: 27
Protein Name: methionine synthase
Accession No.: Cotton_A_18904
Score: 380

## Slide 29
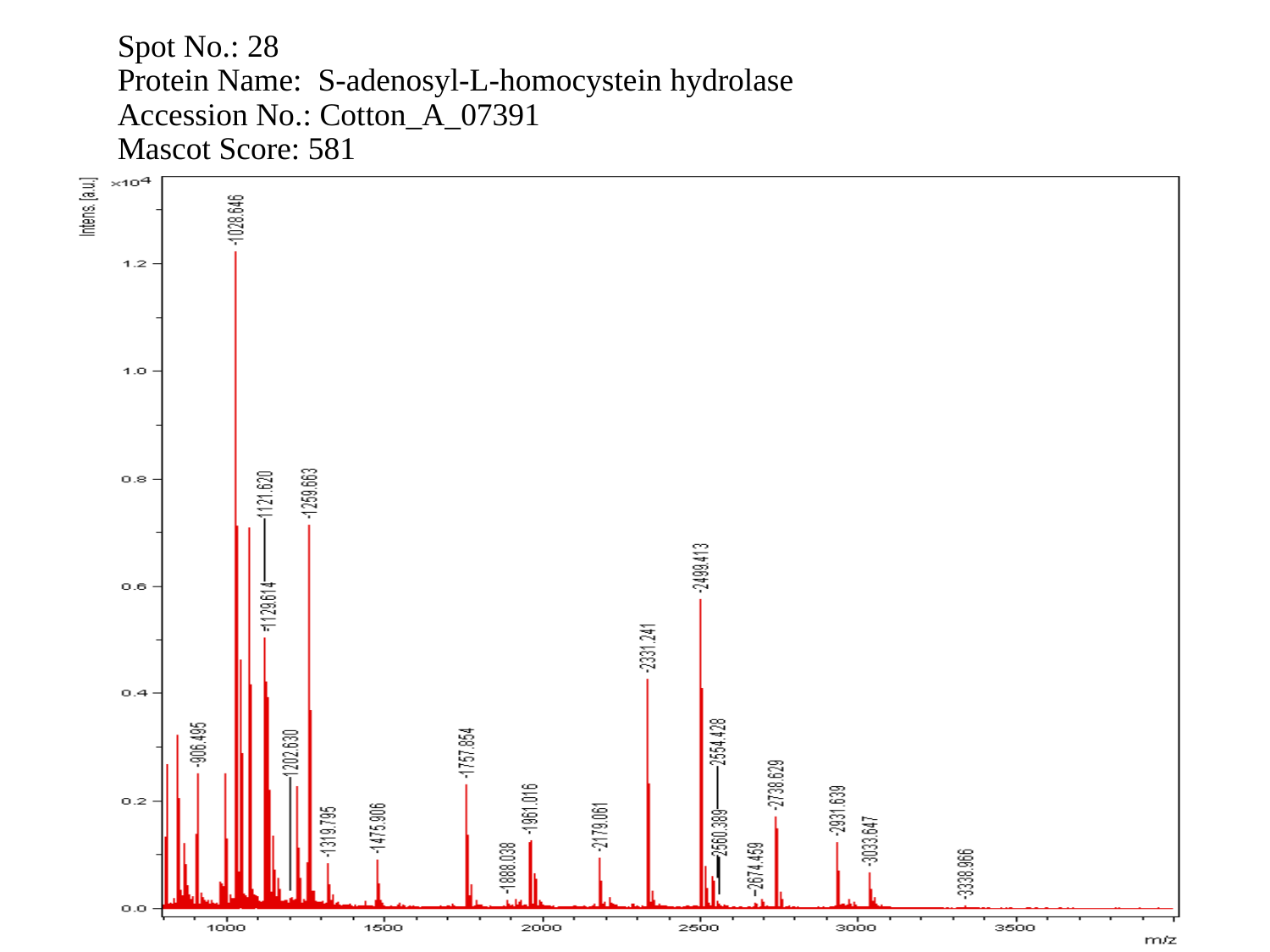

Spot No.: 28
Protein Name: S-adenosyl-L-homocystein hydrolase
Accession No.: Cotton_A_07391
Mascot Score: 581

## Slide 30
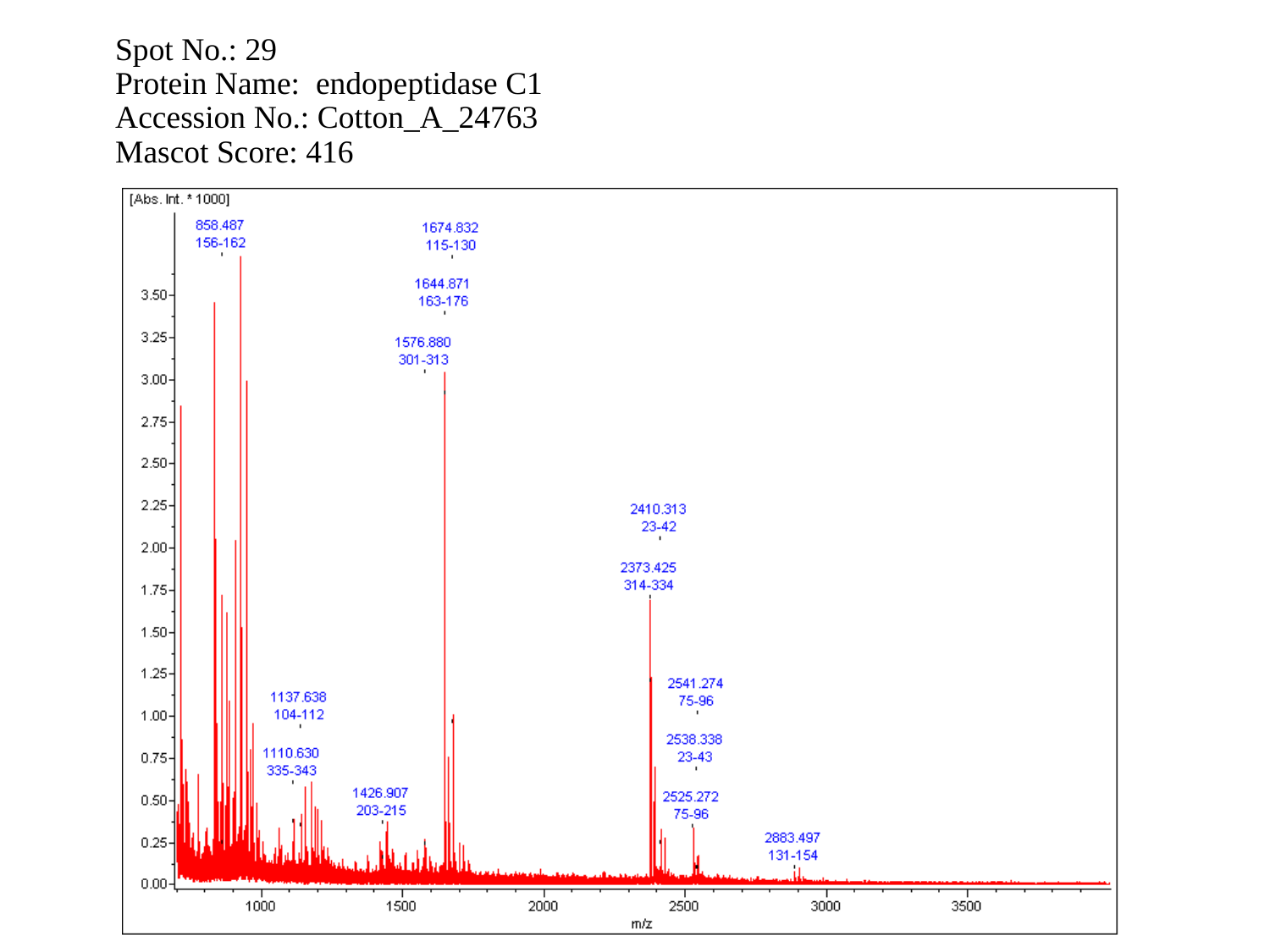

Spot No.: 29
Protein Name: endopeptidase C1
Accession No.: Cotton_A_24763
Mascot Score: 416

## Slide 31
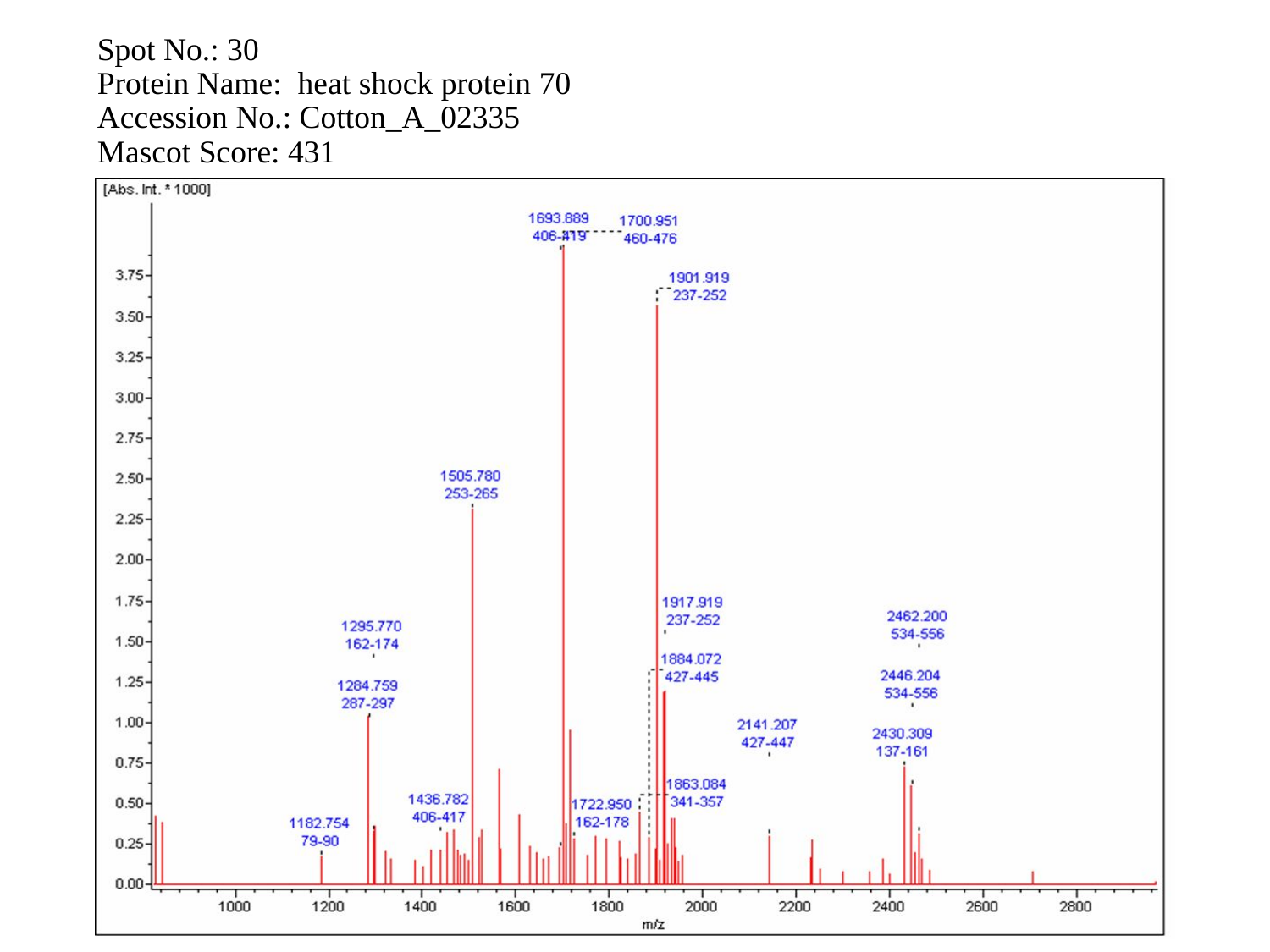

Spot No.: 30
Protein Name: heat shock protein 70
Accession No.: Cotton_A_02335
Mascot Score: 431

## Slide 32
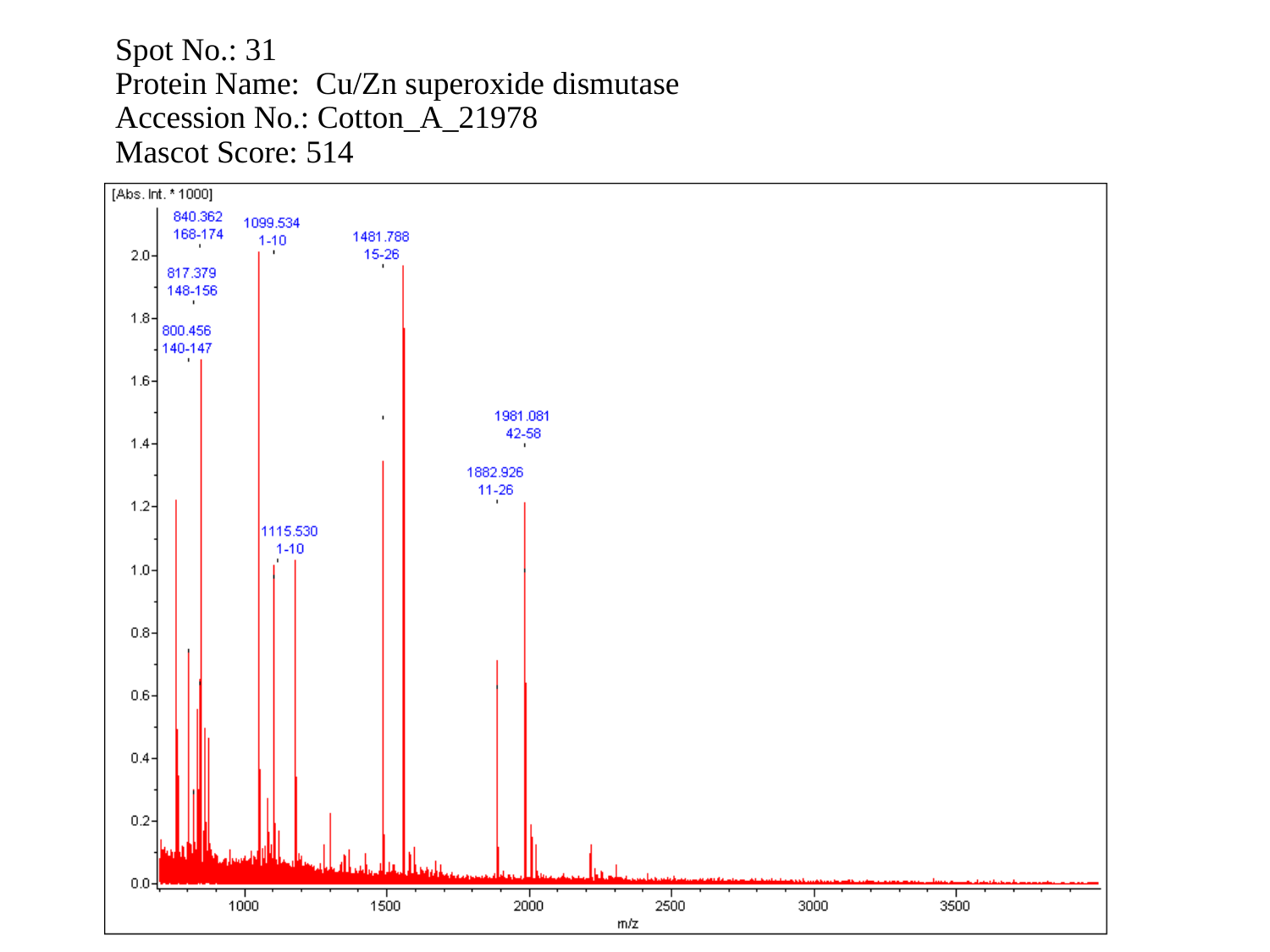

Spot No.: 31
Protein Name: Cu/Zn superoxide dismutase
Accession No.: Cotton_A_21978
Mascot Score: 514

## Slide 33
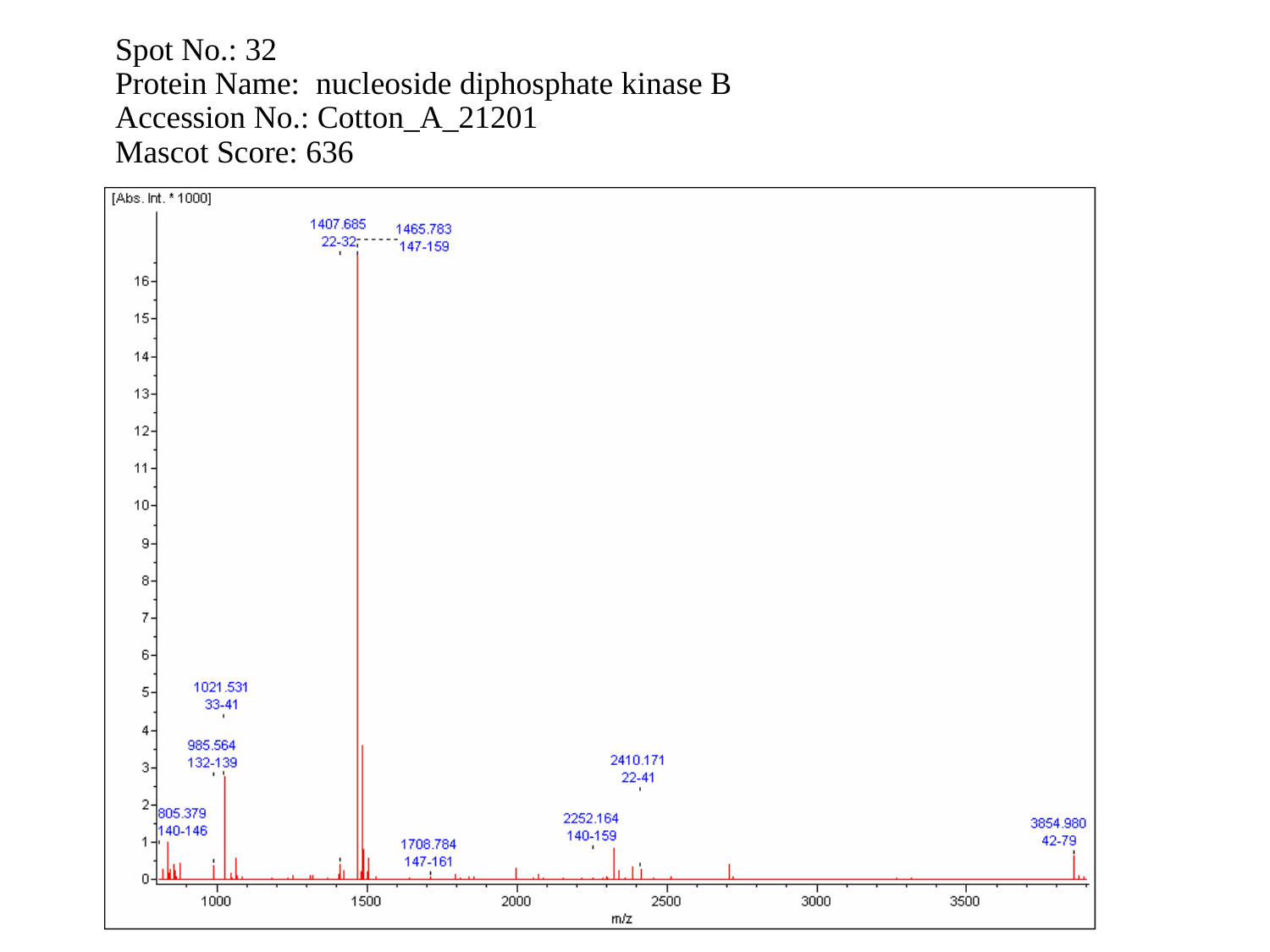

Spot No.: 32
Protein Name: nucleoside diphosphate kinase B Accession No.: Cotton_A_21201
Mascot Score: 636

## Slide 34
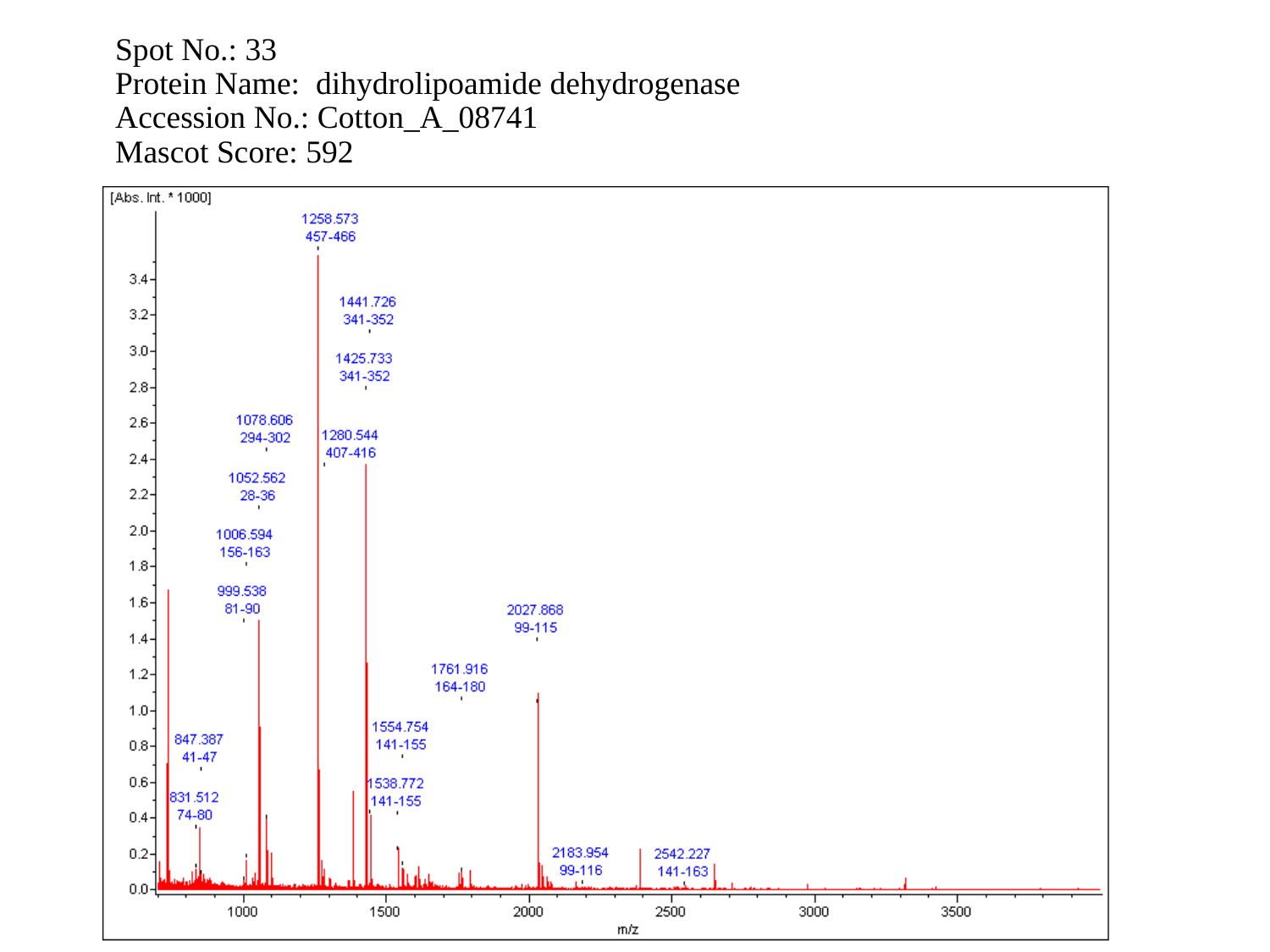

Spot No.: 33
Protein Name: dihydrolipoamide dehydrogenase
Accession No.: Cotton_A_08741
Mascot Score: 592

## Slide 35
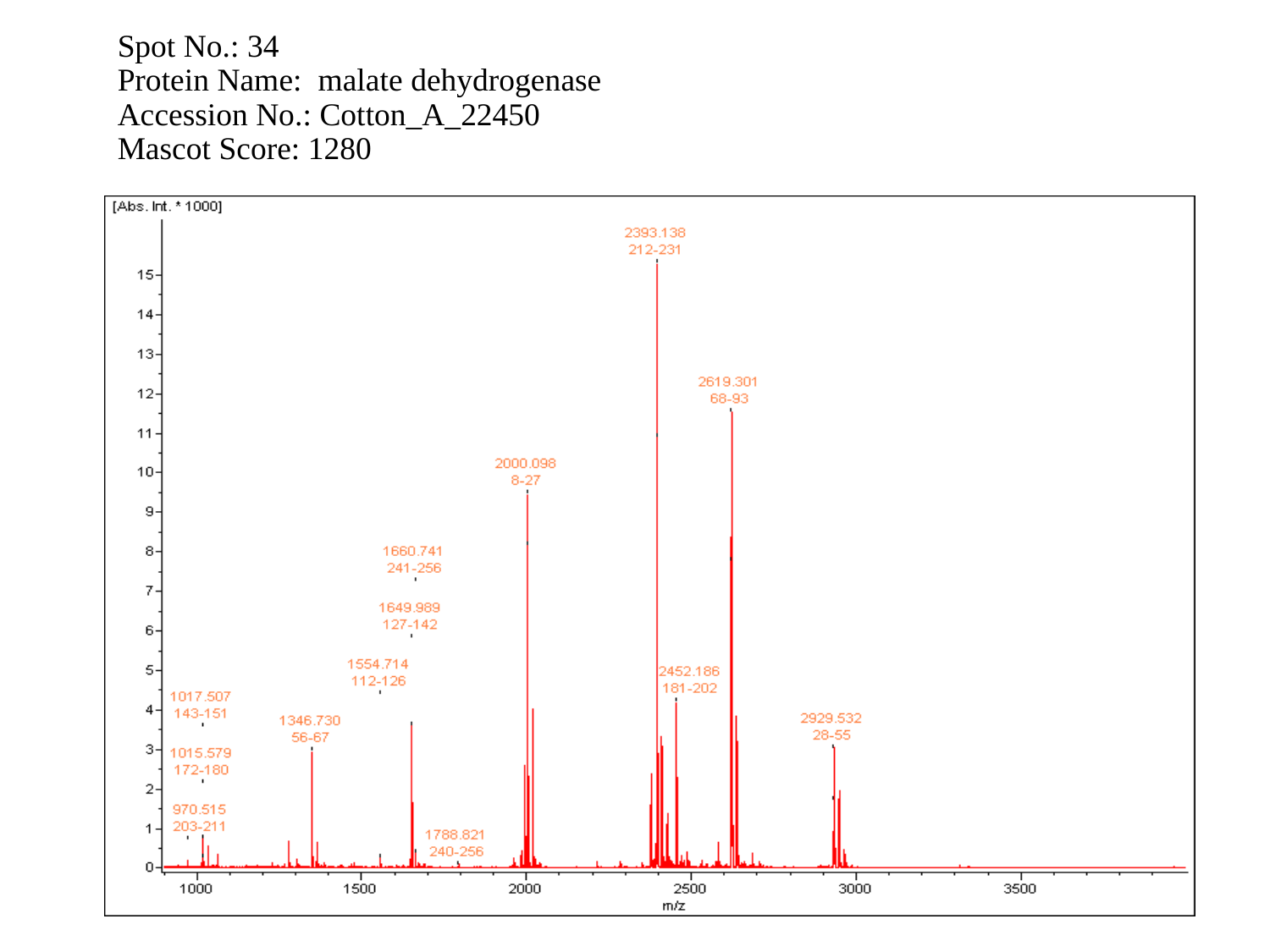

Spot No.: 34
Protein Name: malate dehydrogenase
Accession No.: Cotton_A_22450
Mascot Score: 1280

## Slide 36
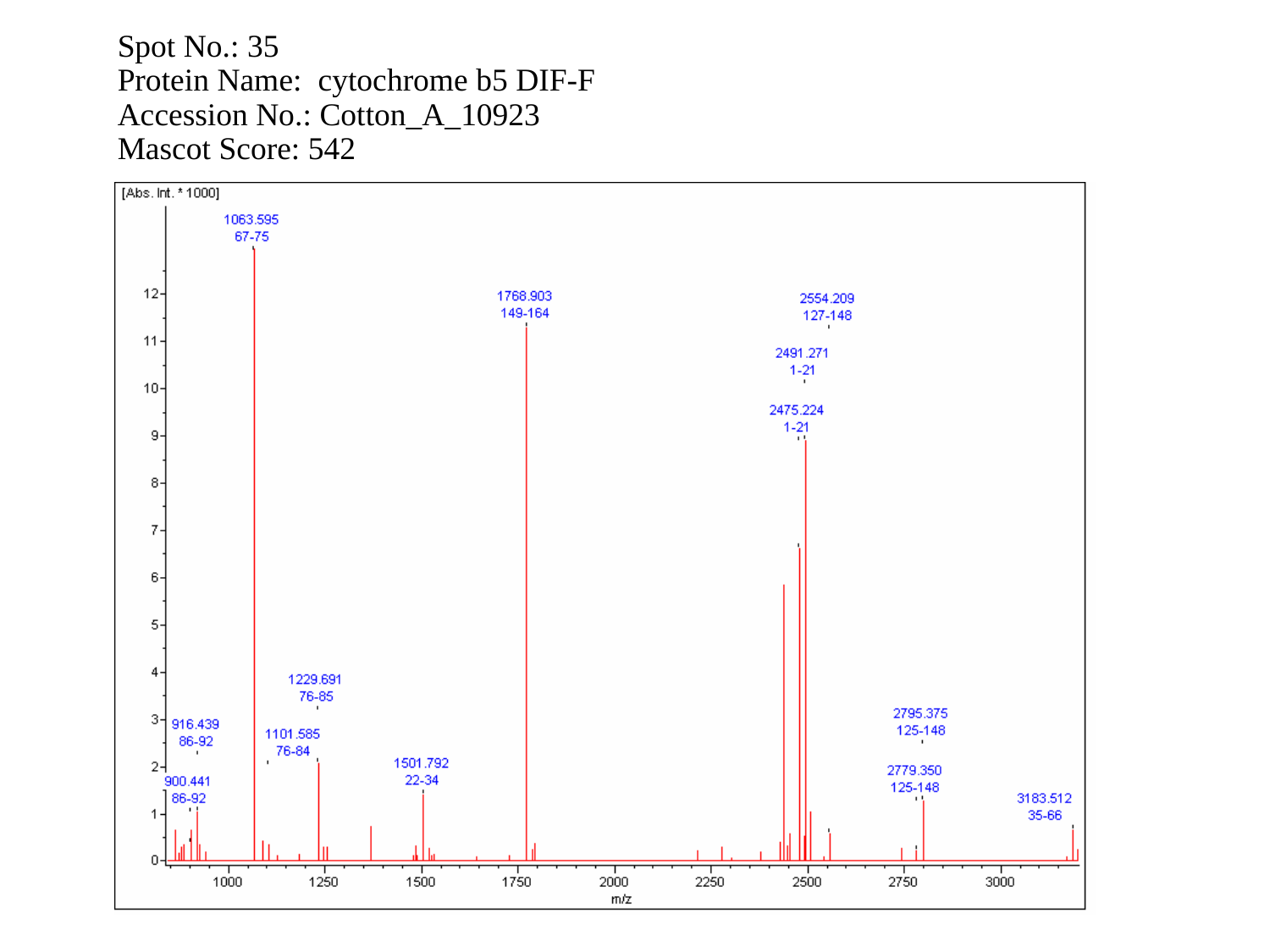

Spot No.: 35
Protein Name: cytochrome b5 DIF-F
Accession No.: Cotton_A_10923
Mascot Score: 542

## Slide 37
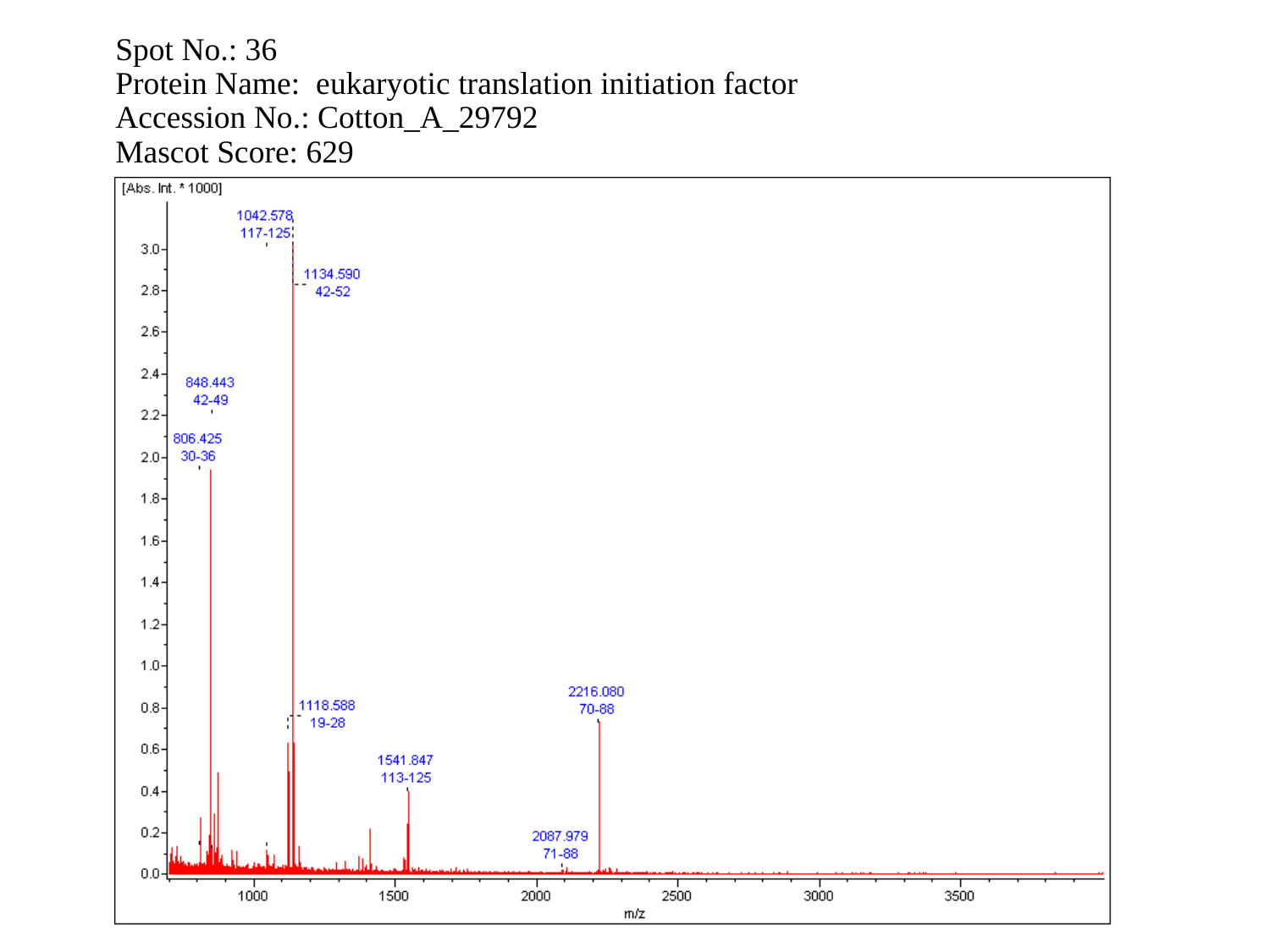

Spot No.: 36
Protein Name: eukaryotic translation initiation factor
Accession No.: Cotton_A_29792
Mascot Score: 629

## Slide 38
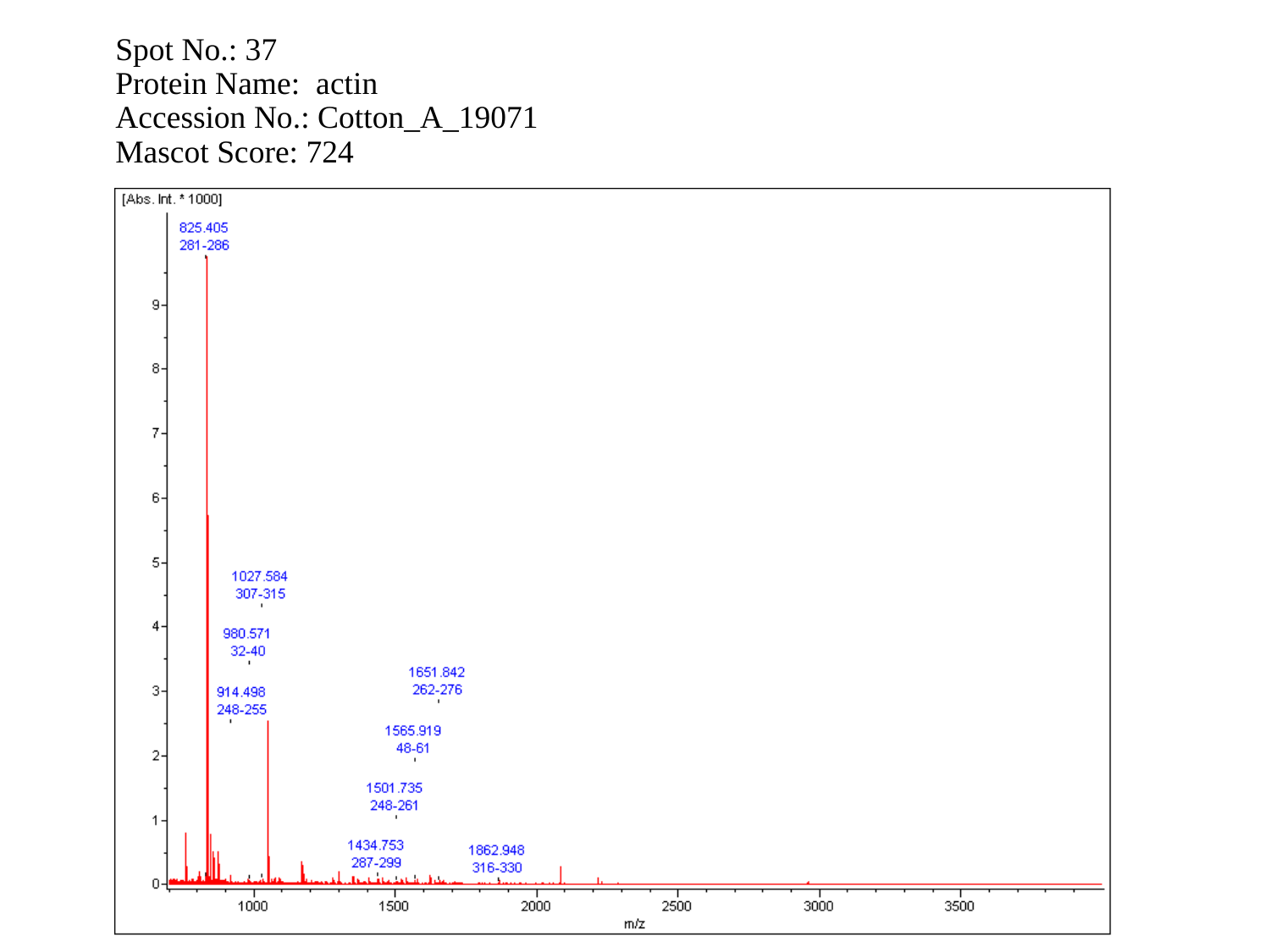

Spot No.: 37
Protein Name: actin
Accession No.: Cotton_A_19071
Mascot Score: 724

## Slide 39
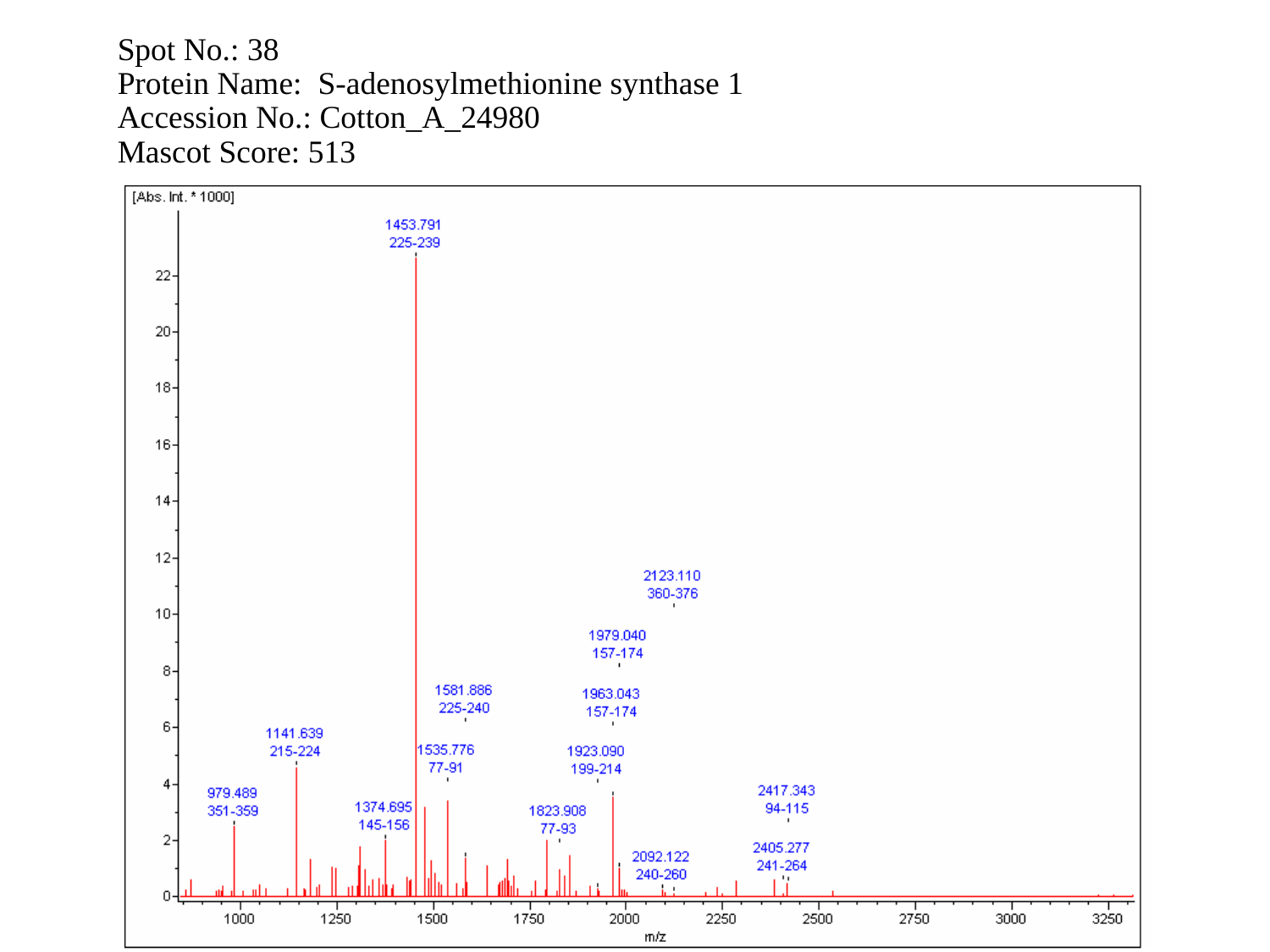

Spot No.: 38
Protein Name: S-adenosylmethionine synthase 1
Accession No.: Cotton_A_24980
Mascot Score: 513

## Slide 40
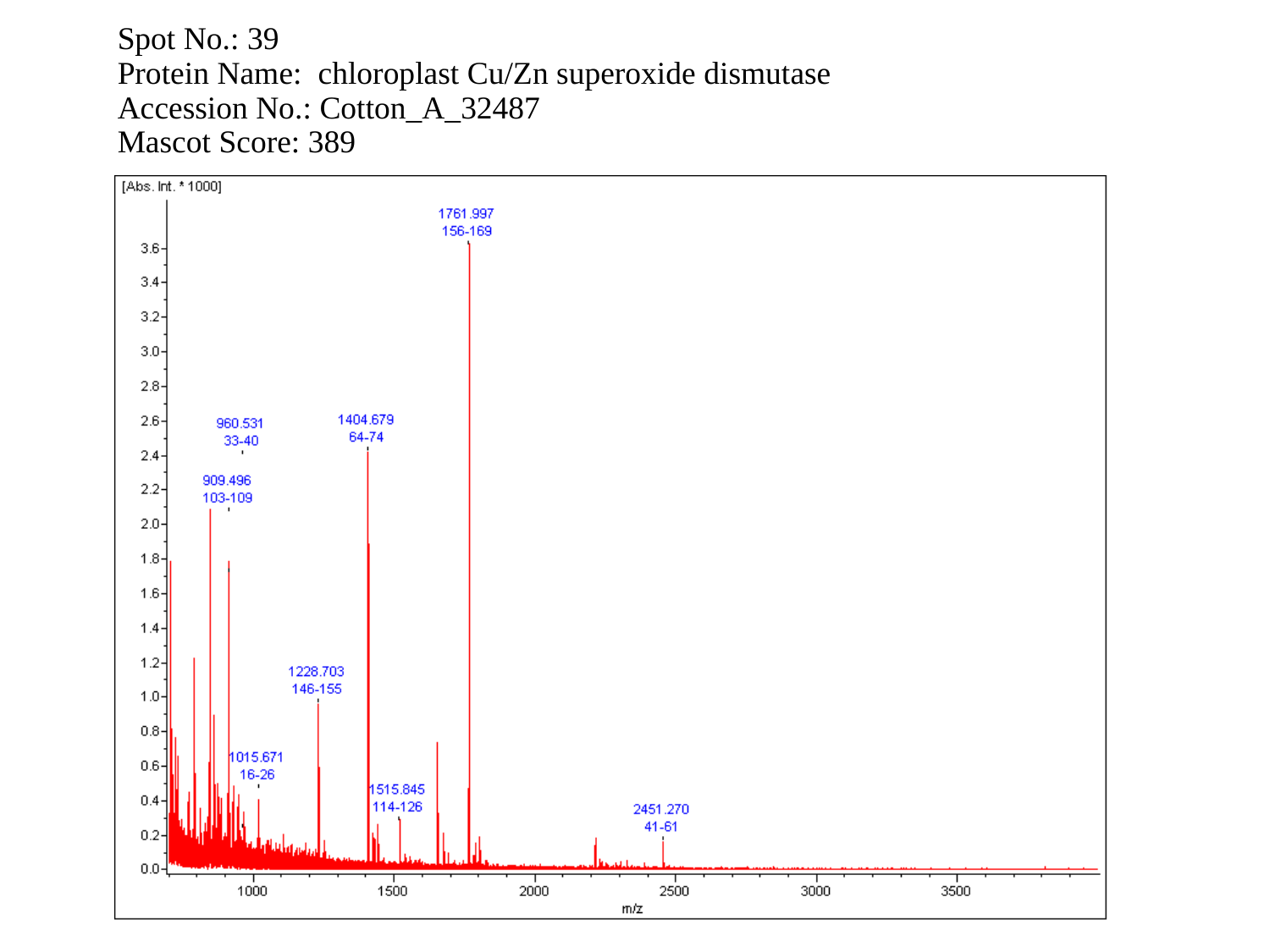

Spot No.: 39
Protein Name: chloroplast Cu/Zn superoxide dismutase
Accession No.: Cotton_A_32487
Mascot Score: 389

## Slide 41
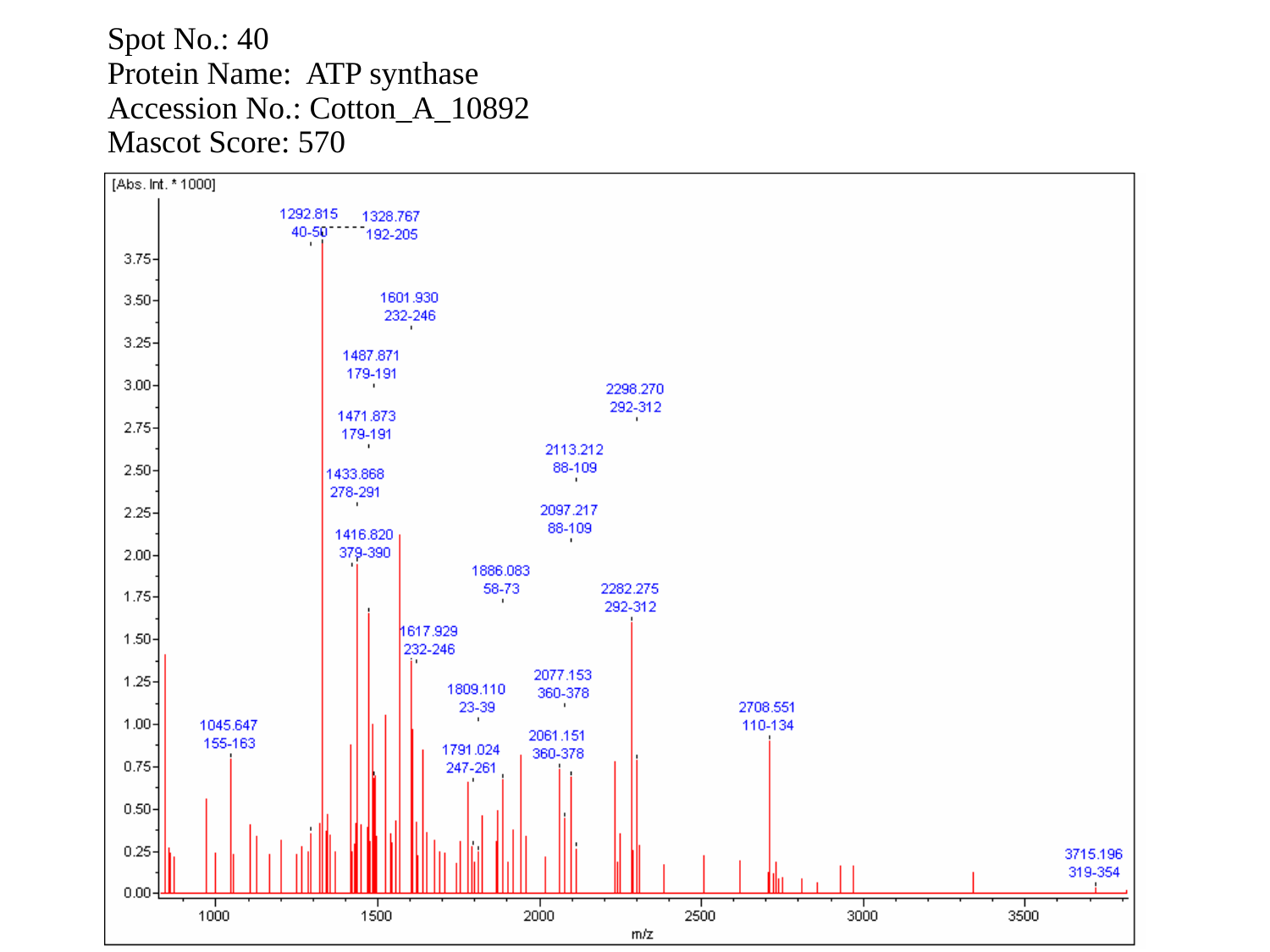

Spot No.: 40
Protein Name: ATP synthase
Accession No.: Cotton_A_10892
Mascot Score: 570

## Slide 42
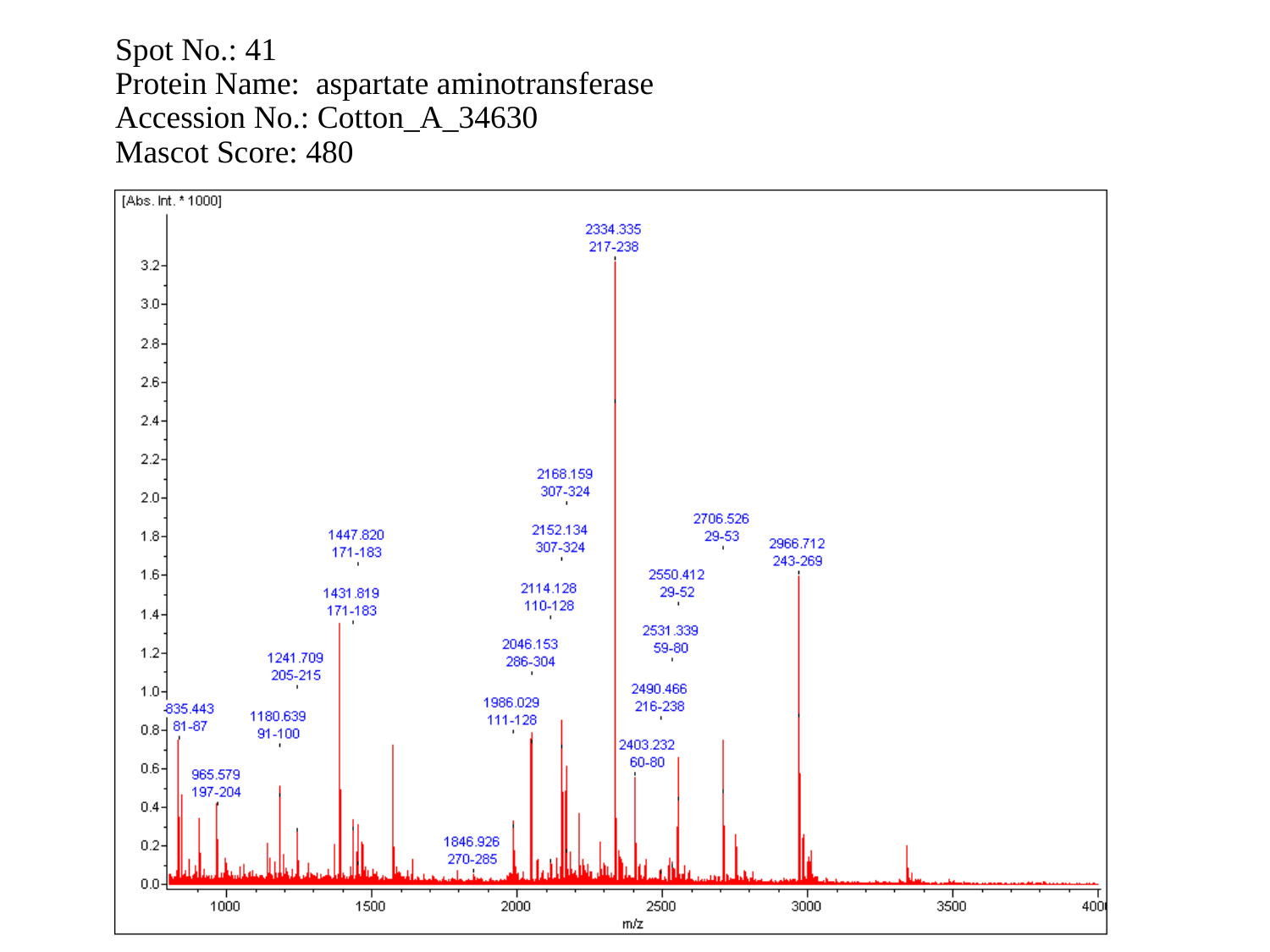

Spot No.: 41
Protein Name: aspartate aminotransferase
Accession No.: Cotton_A_34630
Mascot Score: 480

## Slide 43
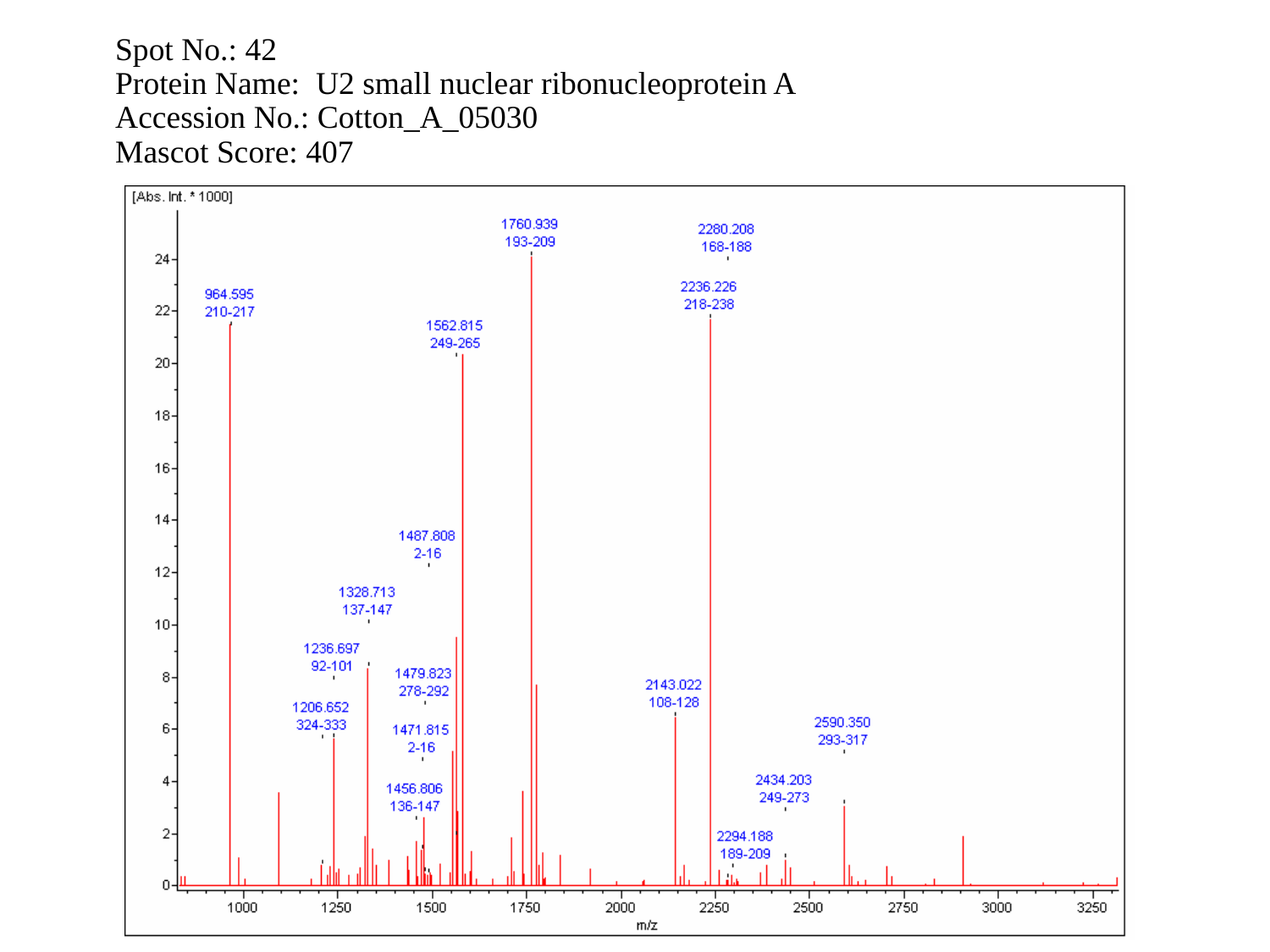

Spot No.: 42
Protein Name: U2 small nuclear ribonucleoprotein A
Accession No.: Cotton_A_05030
Mascot Score: 407

## Slide 44
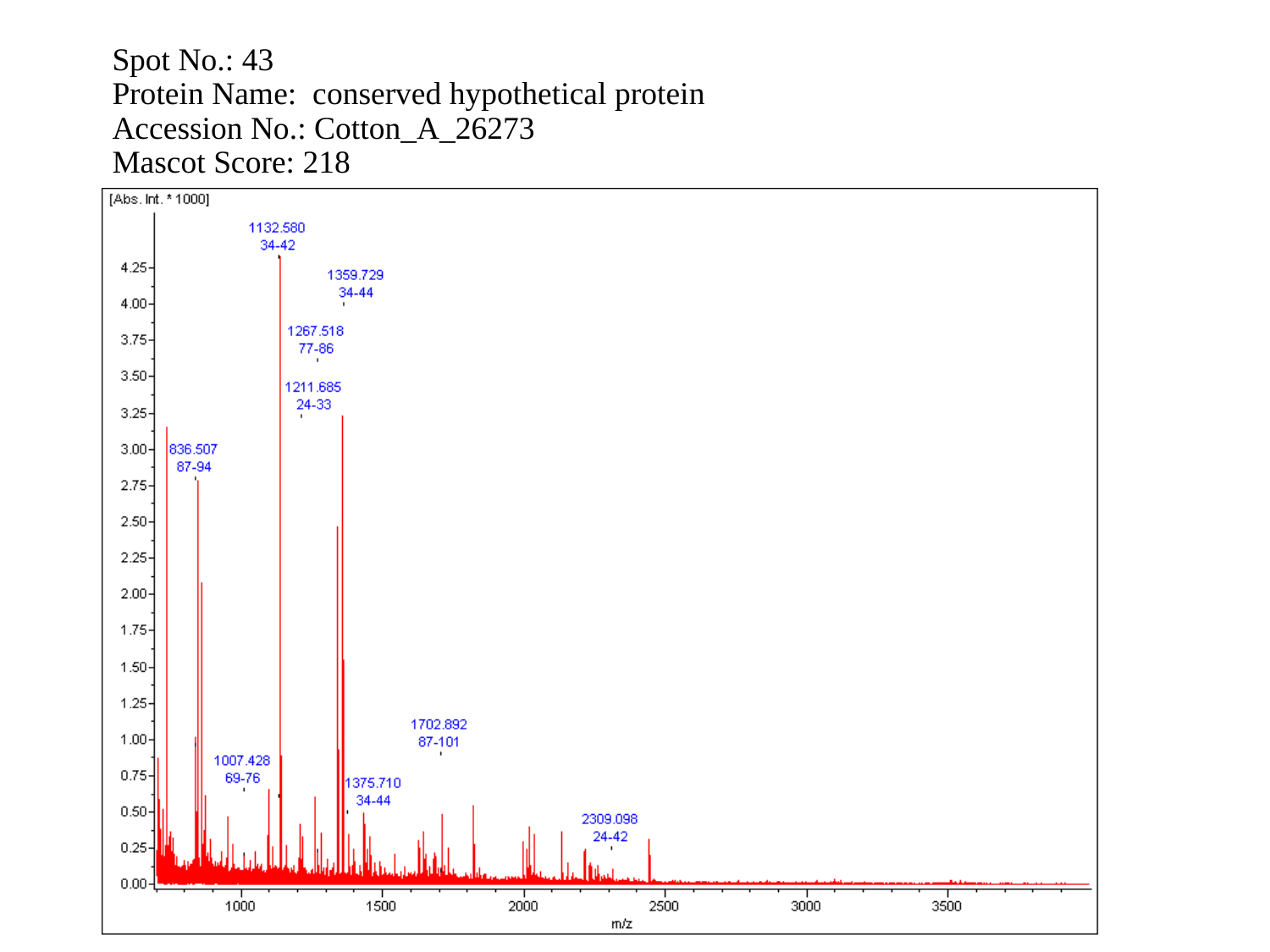

Spot No.: 43
Protein Name: conserved hypothetical protein
Accession No.: Cotton_A_26273
Mascot Score: 218

## Slide 45
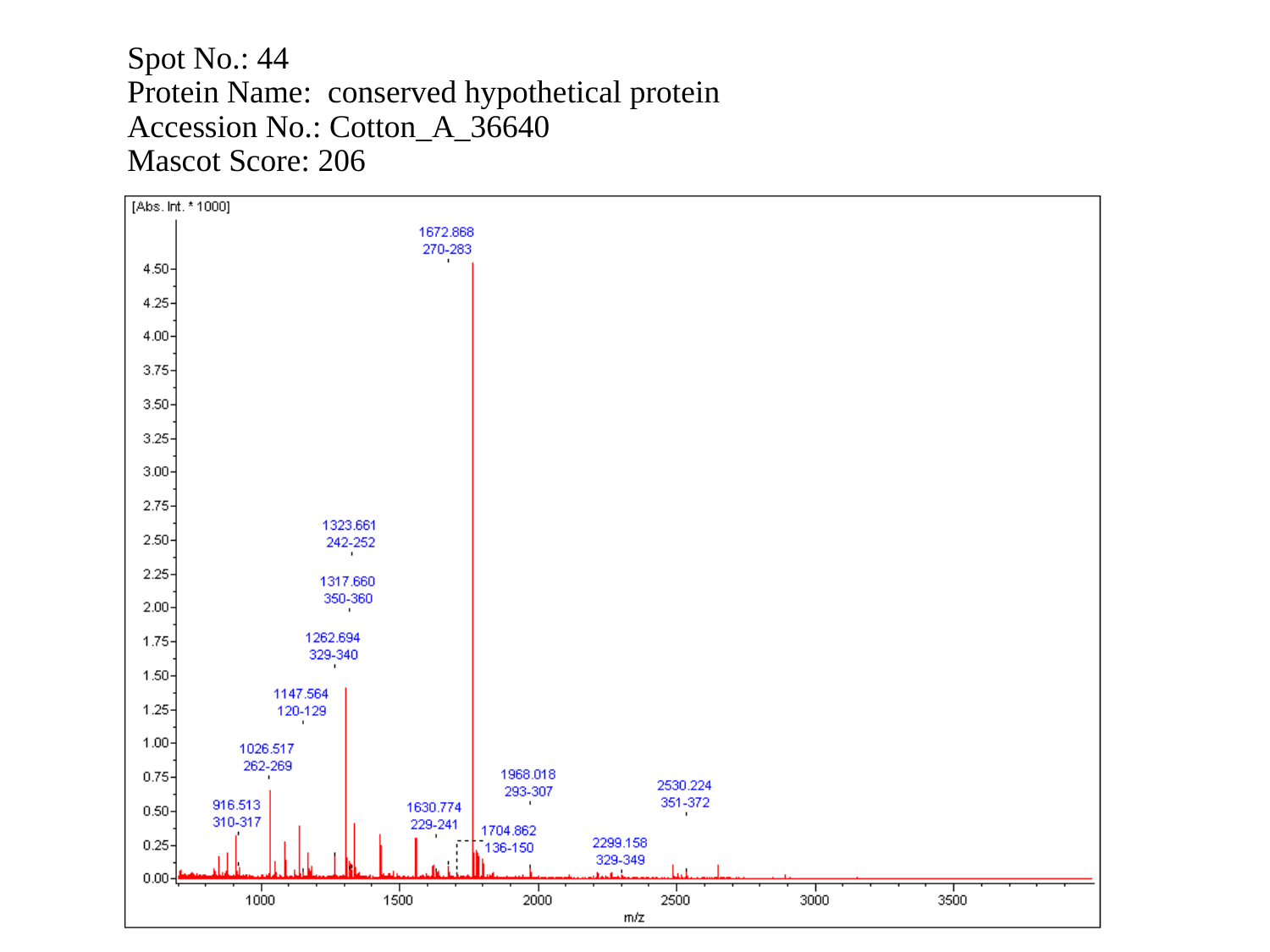

Spot No.: 44
Protein Name: conserved hypothetical protein
Accession No.: Cotton_A_36640
Mascot Score: 206

## Slide 46
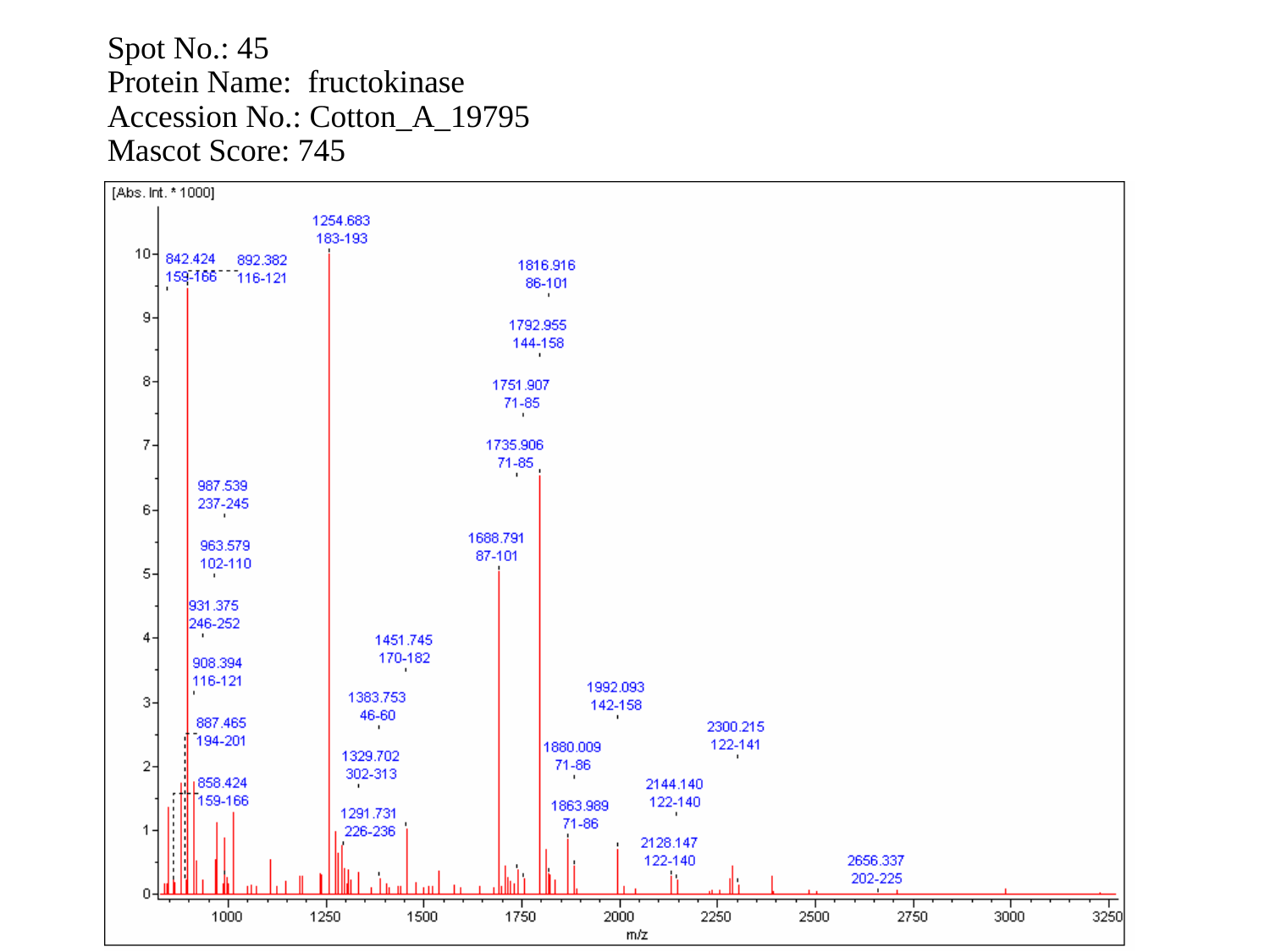

Spot No.: 45
Protein Name: fructokinase
Accession No.: Cotton_A_19795
Mascot Score: 745

## Slide 47
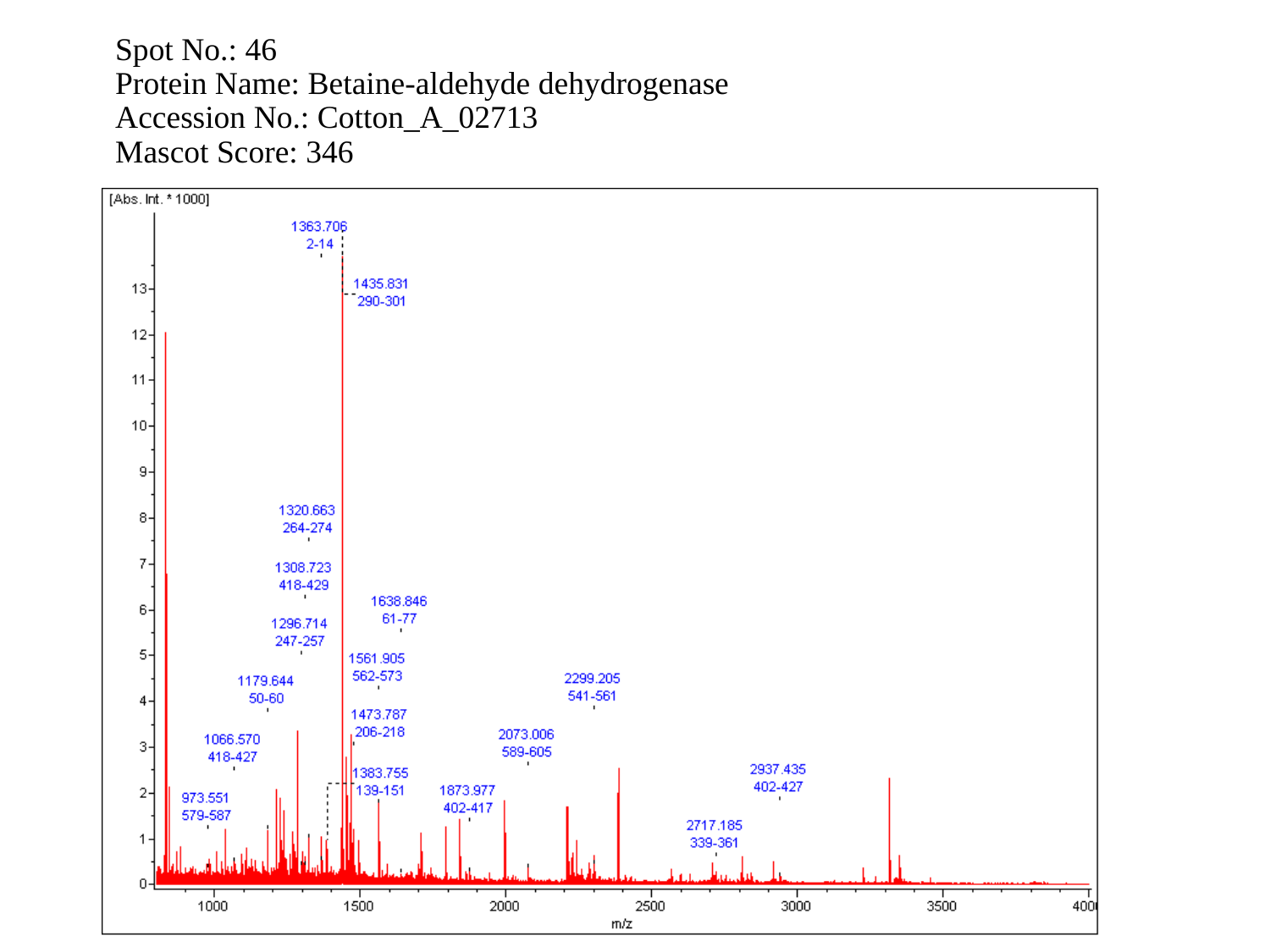

Spot No.: 46
Protein Name: Betaine-aldehyde dehydrogenase
Accession No.: Cotton_A_02713
Mascot Score: 346

## Slide 48
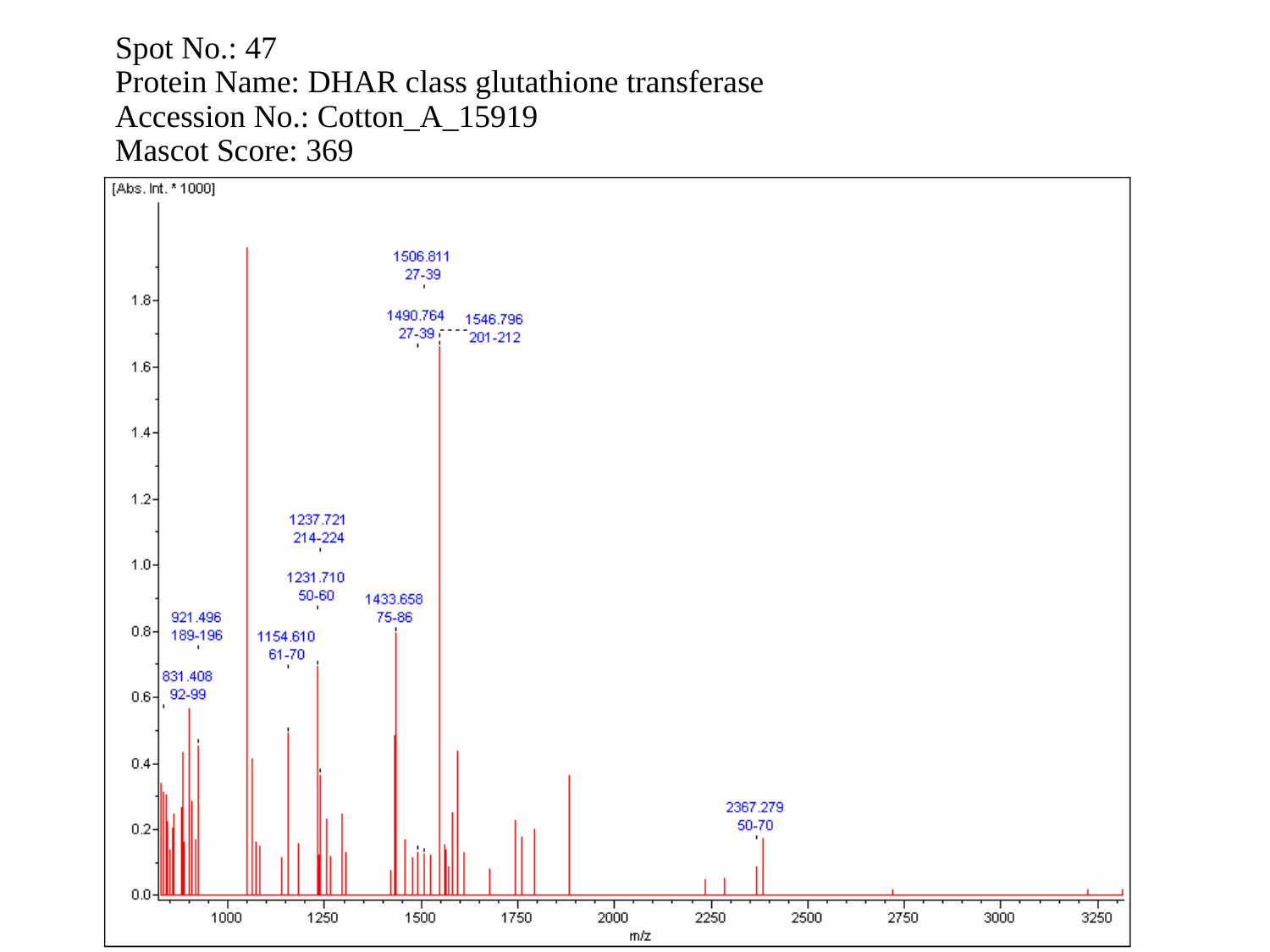

Spot No.: 47
Protein Name: DHAR class glutathione transferase Accession No.: Cotton_A_15919
Mascot Score: 369

## Slide 49
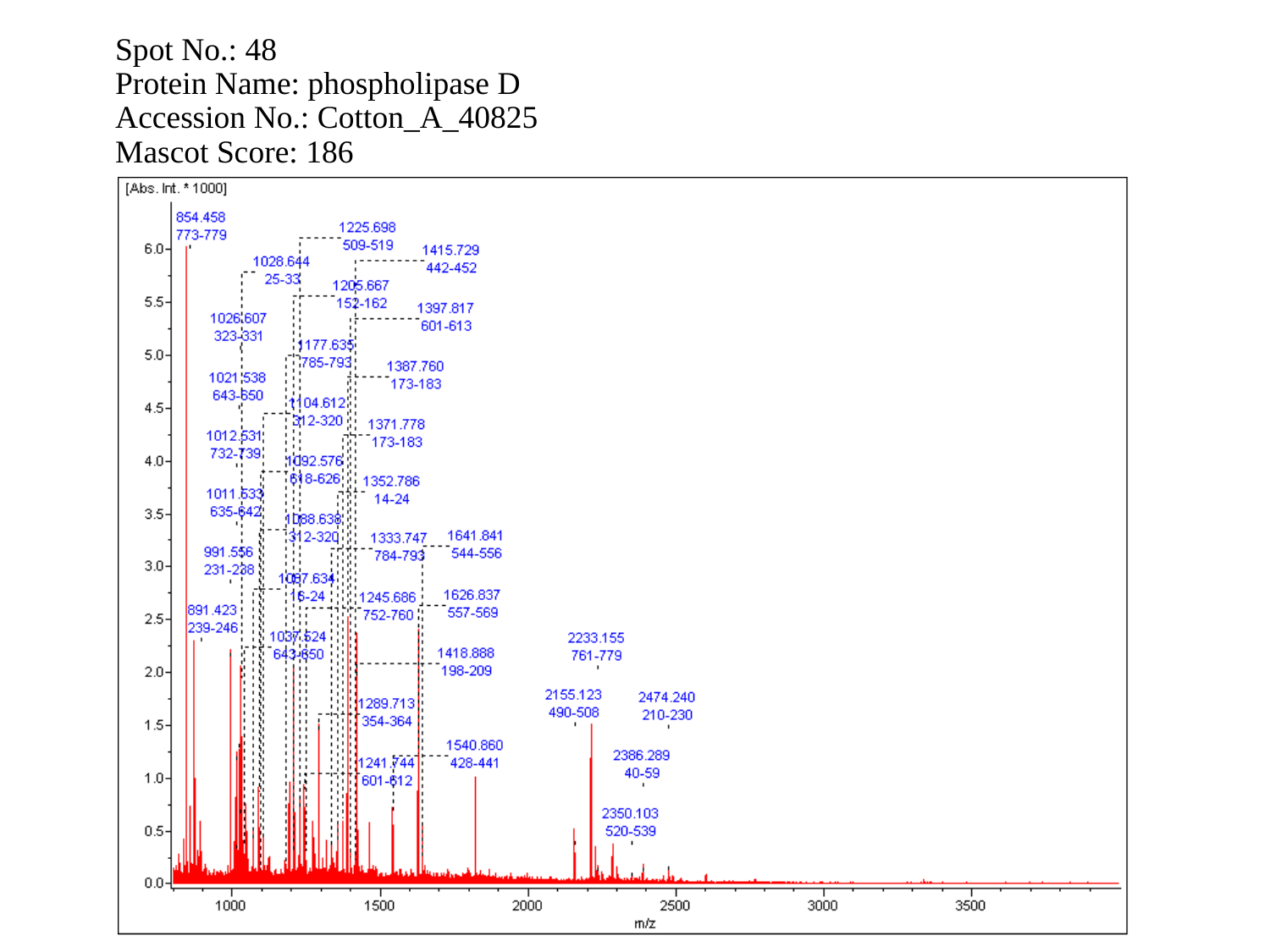

Spot No.: 48
Protein Name: phospholipase D
Accession No.: Cotton_A_40825
Mascot Score: 186

## Slide 50
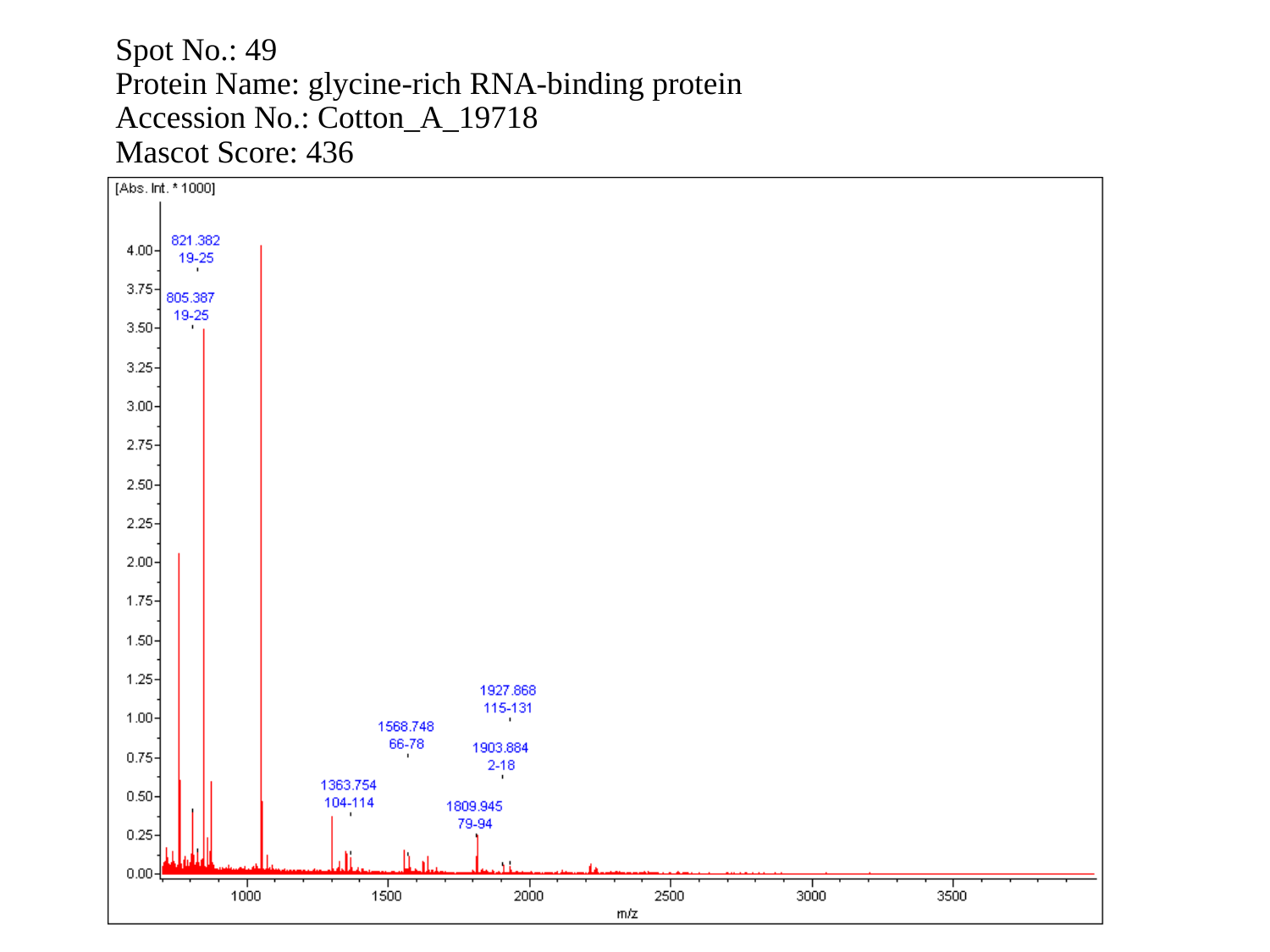

Spot No.: 49
Protein Name: glycine-rich RNA-binding protein
Accession No.: Cotton_A_19718
Mascot Score: 436

## Slide 51
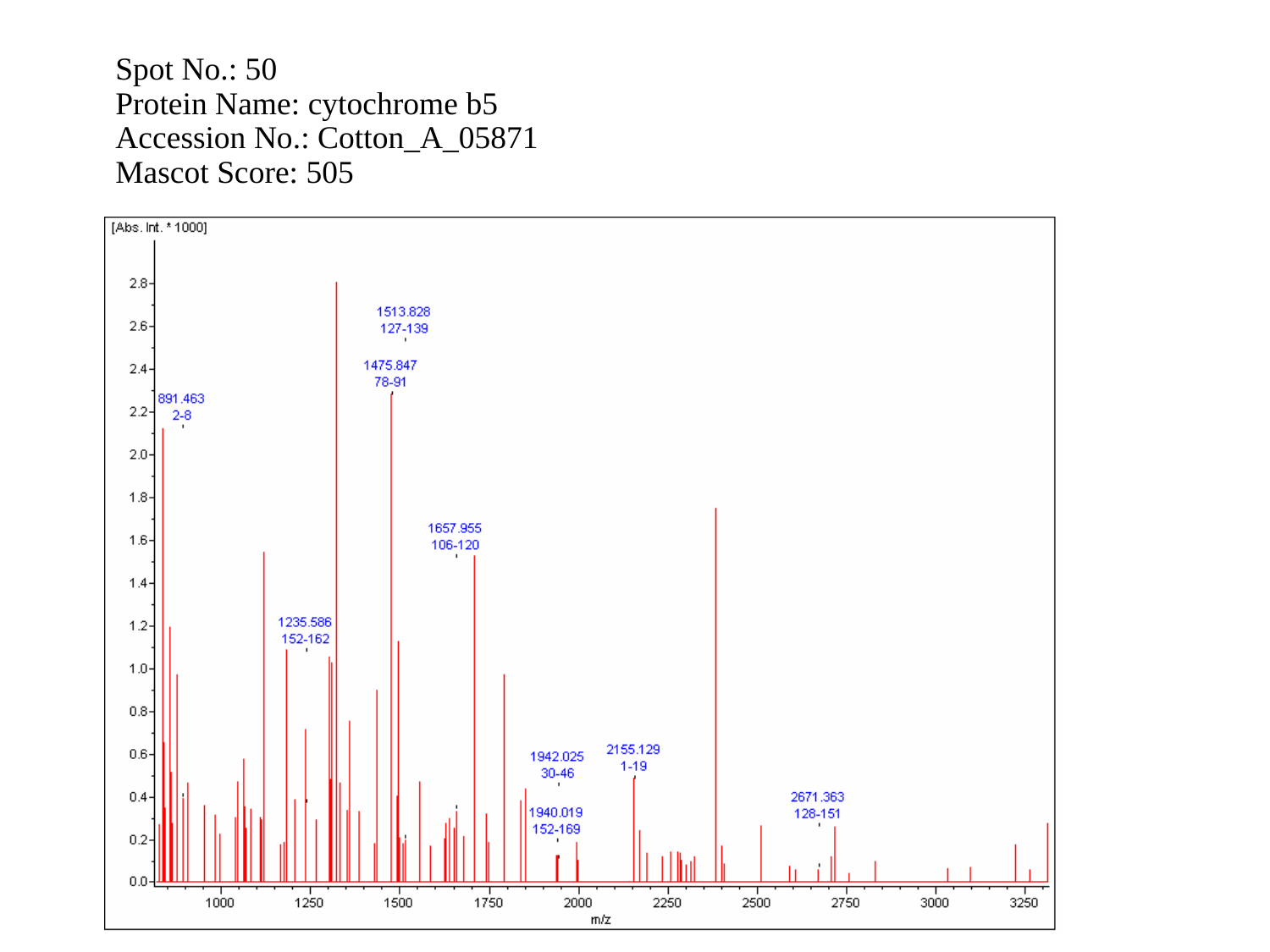

Spot No.: 50
Protein Name: cytochrome b5
Accession No.: Cotton_A_05871
Mascot Score: 505

## Slide 52
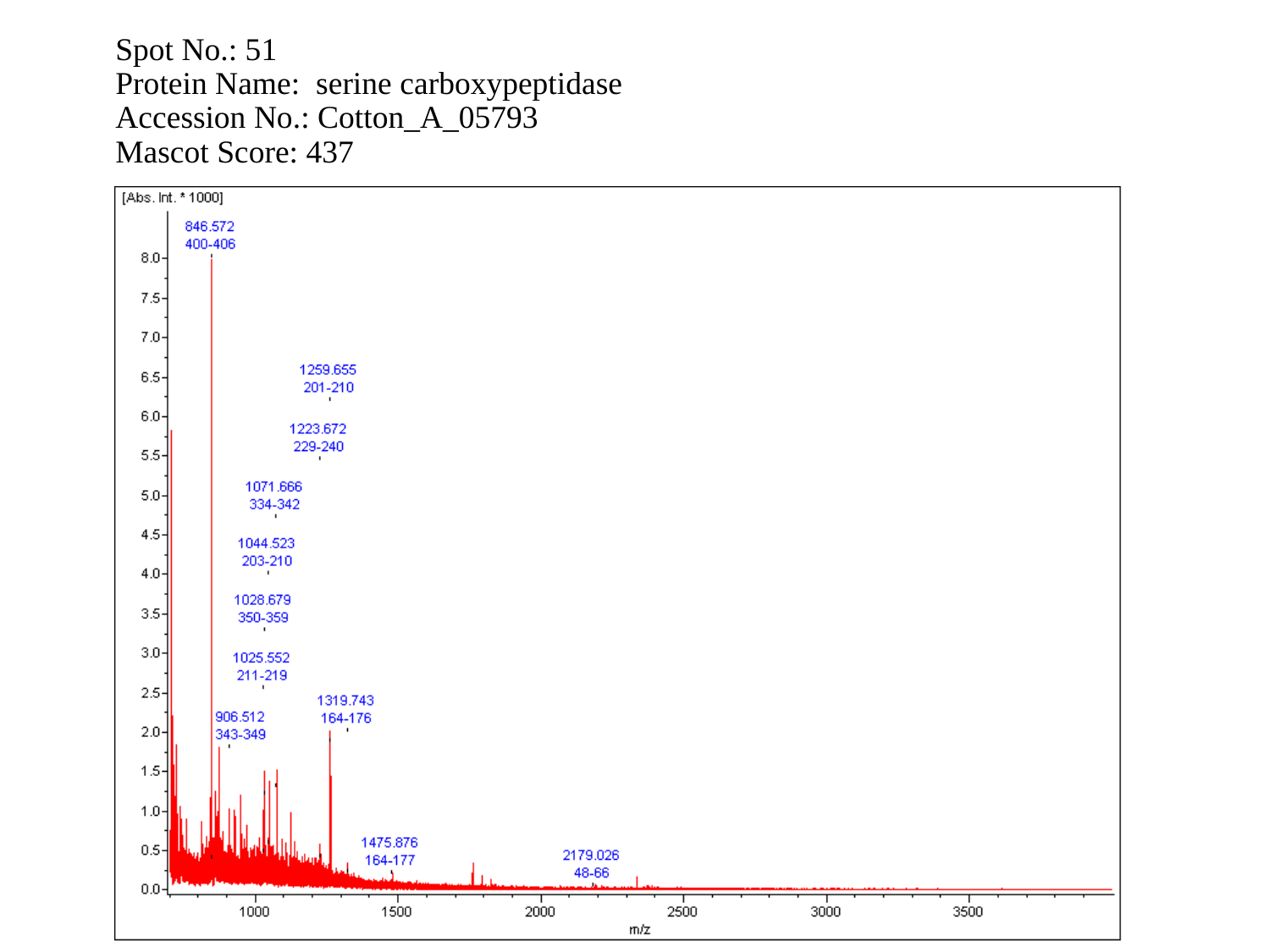

Spot No.: 51
Protein Name: serine carboxypeptidase
Accession No.: Cotton_A_05793
Mascot Score: 437

## Slide 53
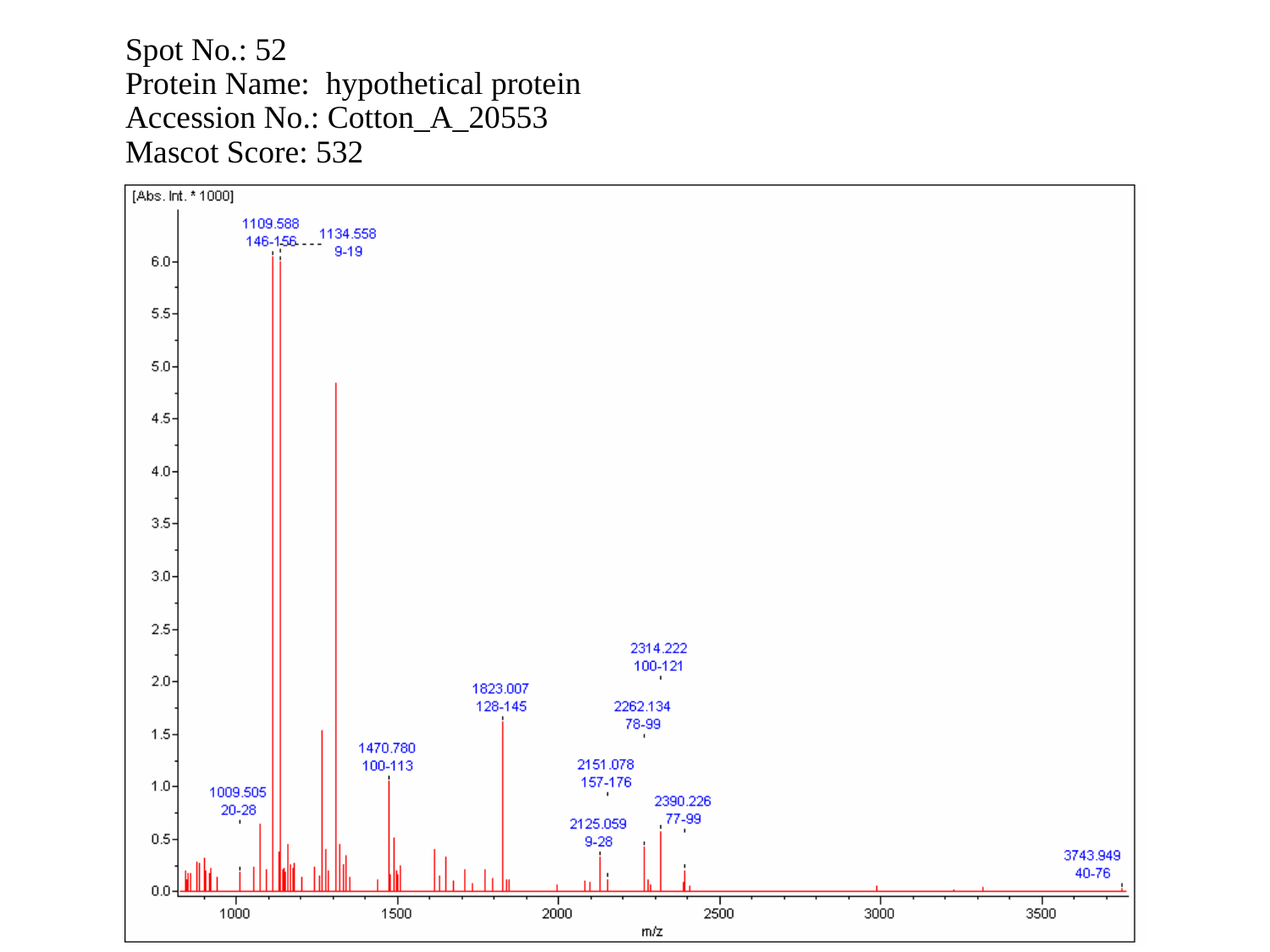

Spot No.: 52
Protein Name: hypothetical protein
Accession No.: Cotton_A_20553
Mascot Score: 532

## Slide 54
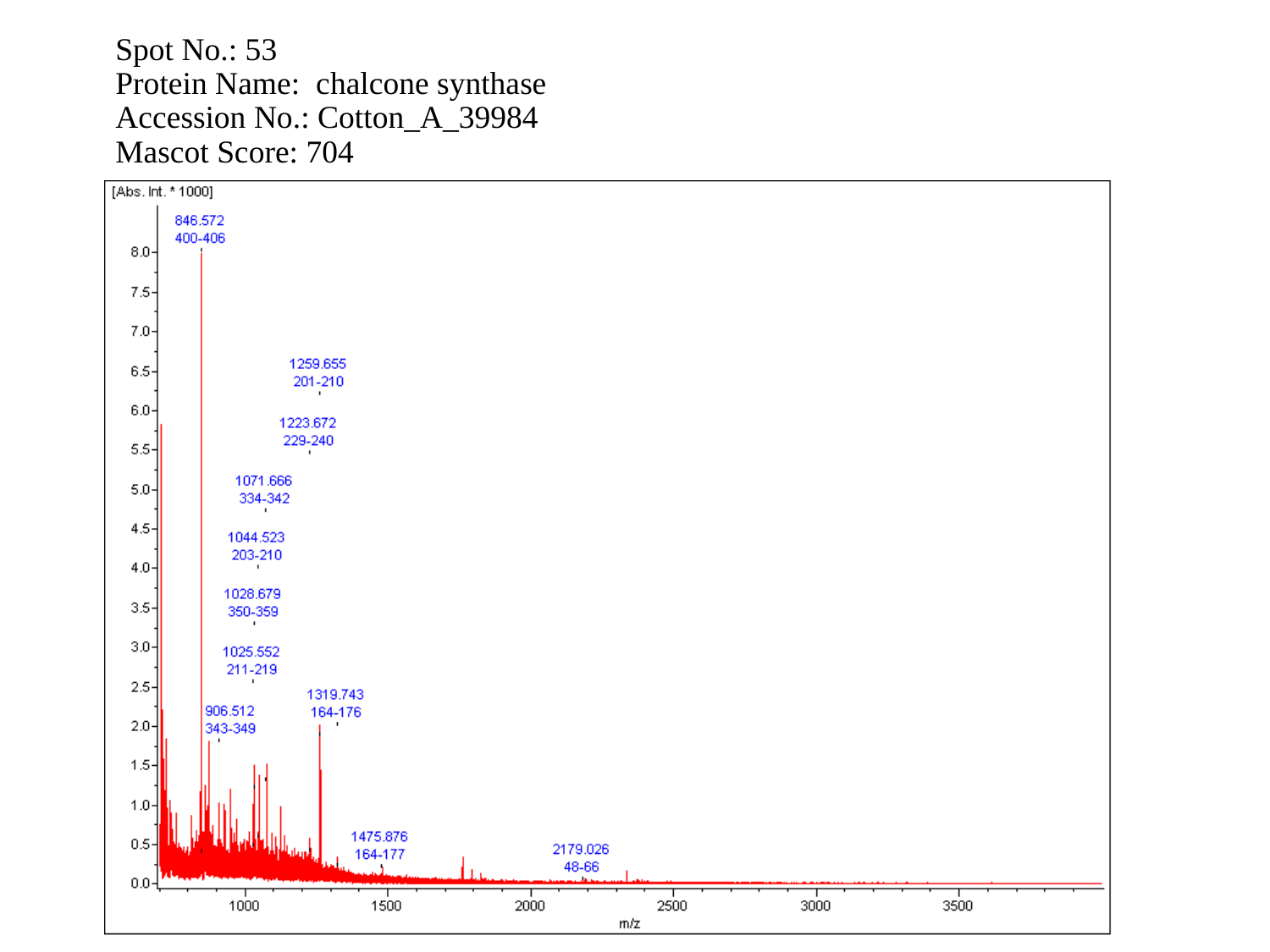

Spot No.: 53
Protein Name: chalcone synthase
Accession No.: Cotton_A_39984
Mascot Score: 704

## Slide 55
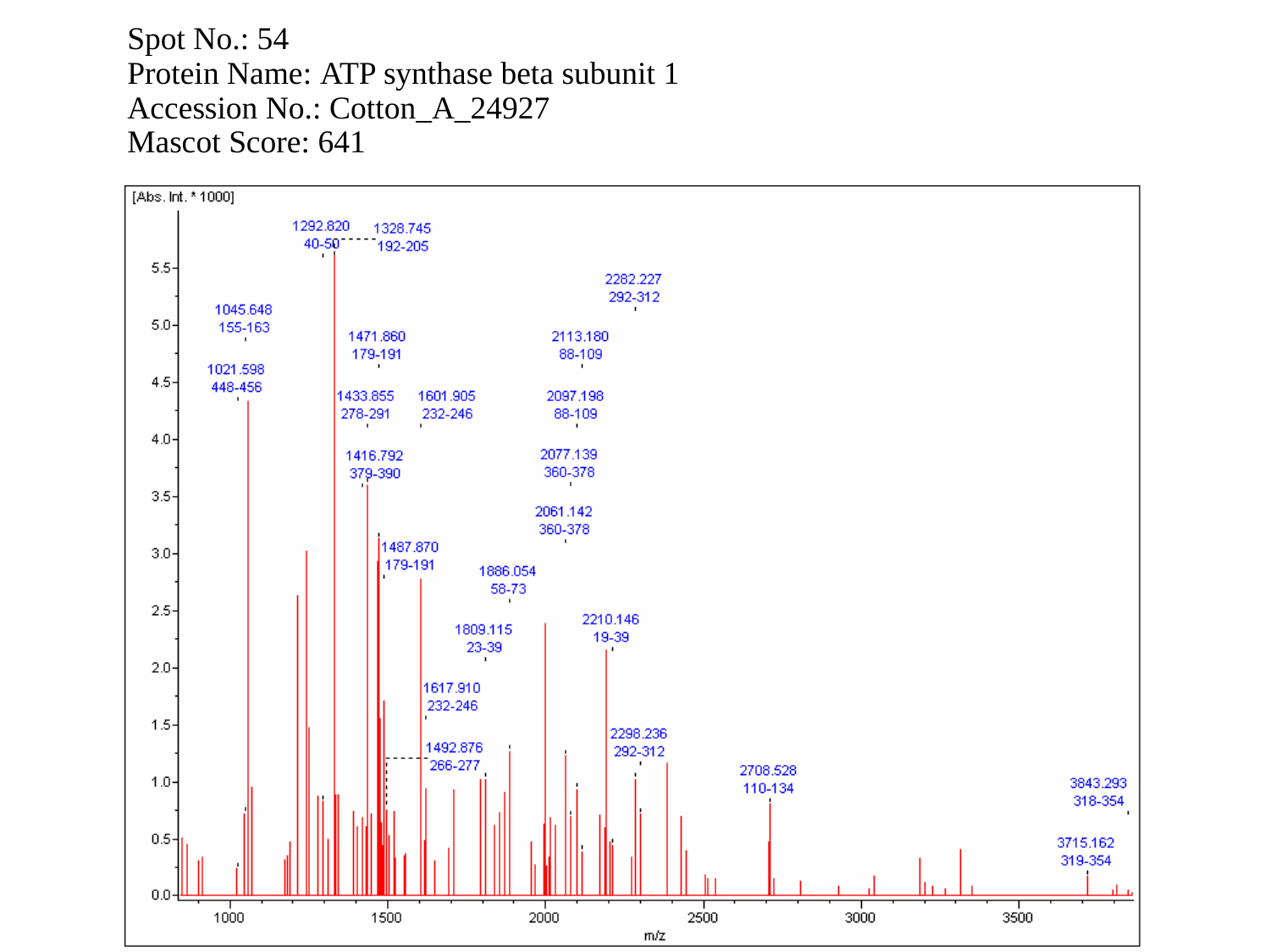

Spot No.: 54
Protein Name: ATP synthase beta subunit 1
Accession No.: Cotton_A_24927
Mascot Score: 641

## Slide 56
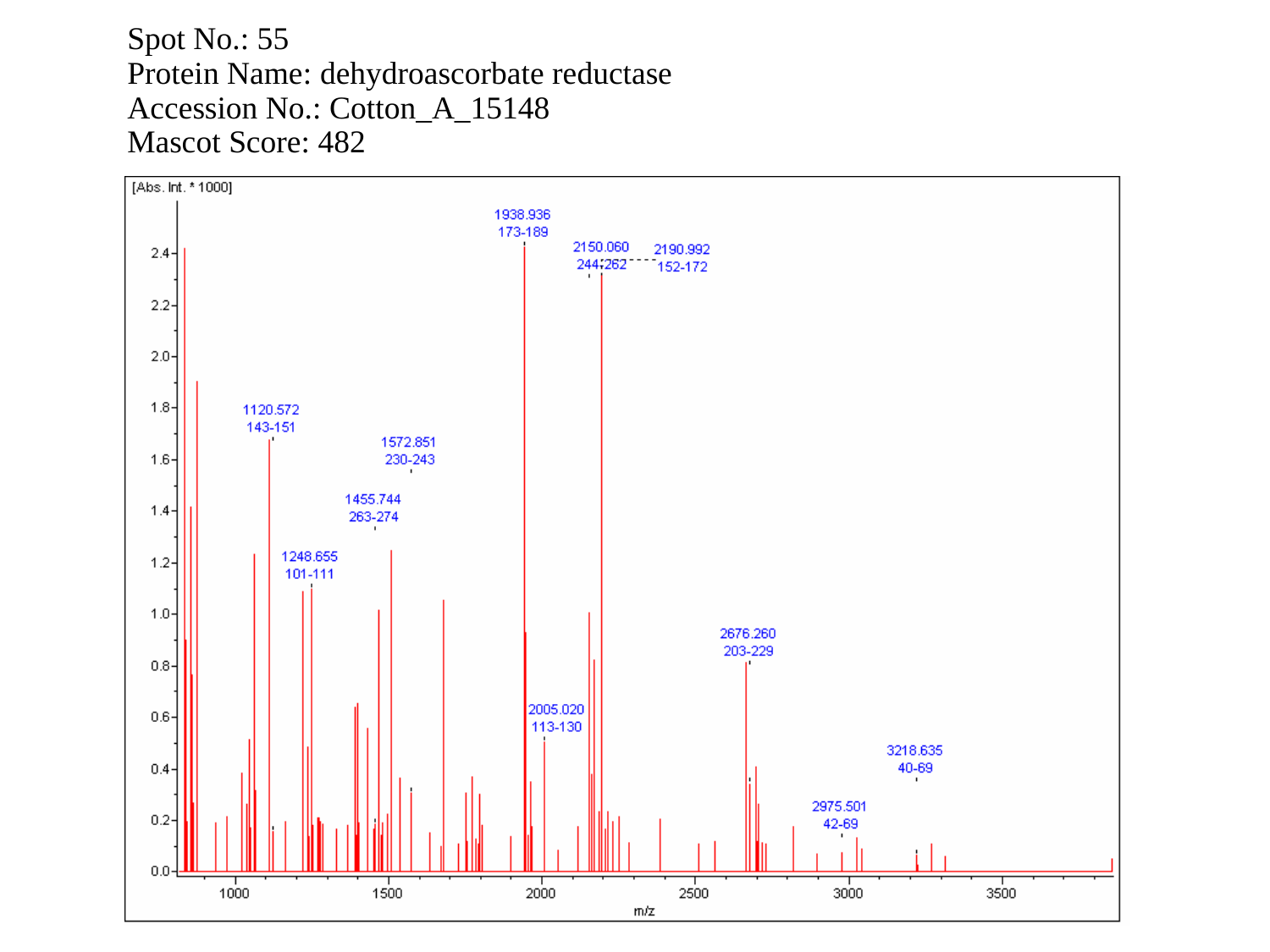

Spot No.: 55
Protein Name: dehydroascorbate reductase
Accession No.: Cotton_A_15148
Mascot Score: 482
